# Advances in Oncohematology Immunotherapy: Monoclonal Antibodies and CAR T-Cell Therapies

**DOI:** 10.3390/cells15161439

**Published:** 2026-08-10

**Authors:** Fanny Mathias, Cyril Fersing, Véronique Bourgarel, Jean Gabert, Florence Peyron

**Affiliations:** 1 CNRS, Institut de Chimie Radicalaire ICR, UMR 7273, Equipe de Pharmaco-Chimie Radicalaire, Aix-Marseille University, 13005 Marseille, France; 2 Pharmacy Department, Marseille Nord University Hospital Pharmacy, Assistance Publique-Hôpitaux de Marseille, 13015 Marseille, France; 3 Nuclear Medicine Department, Institut Régional du Cancer de Montpellier, Université de Montpellier, 34090 Montpellier, France; 4 IBMM, CNRS, ENSCM, Université de Montpellier, 34090 Montpellier, France; 5 Innovation and Clinical Research Department, Assistance Publique Hôpitaux de Marseille (AP-HM), 13005 Marseille, France; 6 Cancer Research Center of Marseille (CRCM), INSERM UMR1068, CNRS UMR7258, Aix-Marseille University, 13005 Marseille, France; 7 Biochemistry and Molecular Biology Laboratory, North University Hospital, 13015 Marseille, France; 8 CNRS, INSERM, CIML, Aix-Marseille University, 13005 Marseille, France

**Keywords:** immunotherapy, oncohematology, CAR T-cells, monoclonal antibodies

## Abstract

The role of immunotherapy in oncohematology is well established. Monoclonal antibodies have undergone substantial development in recent years, targeting a wide range of molecules (including surface antigens, immune checkpoints, and cytokines) and have demonstrated clinical utility across various hematologic malignancies, including leukemias, lymphomas, and myelomas. The emergence of advanced therapy medicinal products (ATMPs), particularly chimeric antigen receptor (CAR) T cells, has provided an additional and promising therapeutic option. This rapidly evolving field continues to expand therapeutic possibilities. The objective of this literature review is to summarize novel immunotherapeutic strategies in oncohematology, including monoclonal antibody- and CAR T-cell-based approaches, and to review the key findings from recent clinical trials.

## 1. Introduction

Hematologic malignancies comprise a heterogeneous group of diseases, including leukemias (acute lymphoblastic leukemia [ALL], chronic lymphocytic leukemia [CLL], acute myeloid leukemia [AML], and chronic myeloid leukemia [CML]), lymphomas (including Hodgkin lymphoma [HL], non-Hodgkin lymphomas [NHLs], follicular lymphoma [FL], and diffuse large B-cell lymphoma [DLBCL]), and plasma cell neoplasms such as multiple myeloma (MM). Despite major therapeutic advances, including intensive chemotherapy, allogeneic hematopoietic stem cell transplantation (HSCT), and the development of targeted therapies, these diseases continue to be associated with substantial morbidity and mortality. Recent epidemiological data from the United States estimate that both the incidence and mortality of hematologic malignancies remain high (Table 1), highlighting the persistent need for innovative therapeutic strategies [1,2].

Preventing relapse or disease progression in hematologic malignancies remains a major clinical challenge, and research in this field continues to be highly active. Hematologic malignancies have some unique characteristics as accessibility of malignant cells (circulating or marrow-based) and well-characterized antigenic targets (e.g., CD19, CD20, BCMA) that make these cancers well-suited as targets for immunotherapy [3]. Over the past two decades, immunotherapy has undergone remarkable development in oncohematology, with the emergence of numerous approaches including monospecific (mAbs), bispecific (bsAbs), and trispecific (tsAbs) monoclonal antibodies; antibody–drug conjugates (ADCs); adoptive cell therapies such as chimeric antigen receptor (CAR) T-cell therapy; immune checkpoint inhibitors; and cancer vaccines [4].

Among these strategies, mAbs and CAR T-cell therapies have profoundly transformed the therapeutic landscape of several hematologic malignancies and continue to drive innovation in the field. In this context, the aim of this review is to provide an overview of mAb- and CAR T-cell-based therapies developed in recent years and to discuss their current clinical applications as well as future perspectives in oncohematology. Approved agents and those currently under development will be reviewed through an analysis of the major published clinical trials, with clarification of their respective stages of development and their integration into clinical practice. Ongoing clinical trials with robust methodologies—including large patient populations and comparisons against the standard of care, will also be discussed—even if their results are not yet available. Regarding CAR T-cell therapies, the novelty of this review is to provide a comprehensive overview of clinical studies investigating anti-AML CAR T therapies and identifying the most extensively studied targets. All CAR-based approaches have been covered, including CAR-NK cells and in vivo CAR therapies.

## 2. Monoclonal Antibodies

**Figure 1:**
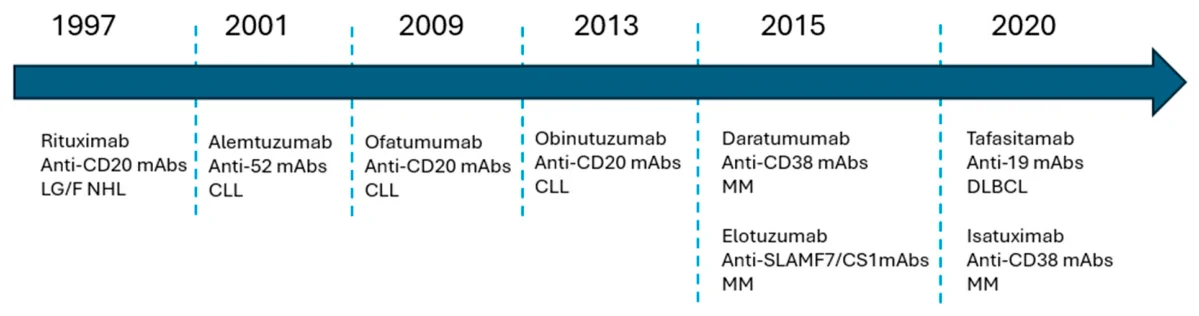
History of FDA-approved mAbs.

Monoclonal antibody therapy emerged as a major therapeutic strategy in oncohematology during the 1990s, marked by the U.S. Food and Drug Administration (FDA) approval of the first anti-CD20 mAb, rituximab, in 1997 [5]. Over the following decades, both the number of therapeutic targets and the spectrum of clinical indications expanded considerably, alongside major advances in antibody engineering, including the development of humanized and fully human mAbs. Many of these agents have since received FDA approval and are now integral components of first- and second-line treatment regimens for a wide range of hematologic malignancies, including NHL, CLL, MM, and DLBCL (Figure 1). The lack of commercialization of new monoclonal antibodies since 2020 can be explained by the emergence and widespread adoption of bispecific antibodies and antibody–drug conjugates (ADCs) [6].

Clinical trials in oncohematology continue to expand rapidly across multiple therapeutic targets, reflecting the increasing complexity and diversification of immunotherapeutic approaches. Accordingly, recent advances can be usefully discussed according to their principal molecular targets and mechanisms of action.

### 2.1. Anti-CD20 mAbs

Since its FDA approval in 1997 for relapsed or refractory (R/R) CD20-positive B-cell low-grade or follicular NHL [5], rituximab has become a cornerstone of therapy for several B-cell malignancies. It is currently used as first-line therapy in combination with chemotherapy for NHL and as monotherapy in selected patients with relapsed disease. In CLL, rituximab is routinely administered in combination with chemotherapy [7].

Rituximab is also increasingly used as a pretreatment prior to the administration of bispecific antibodies (bsAbs), such as epcoritamab, to reduce the risk of cytokine release syndrome (CRS) and improve treatment tolerability [8].

Numerous second-generation anti-CD20 mAbs, including veltuzumab (NCT00547066) [9], ocrelizumab (NCT02723071), and ocaratuzumab (NCT00354926) [10], have been developed. However, none progressed beyond phase I/II clinical trials in hematologic malignancies. Ublituximab advanced to phase III clinical study (NCT02612311), but its development in oncohematology was ultimately discontinued because of the emergence of alternative therapeutic strategies, particularly CAR T-cell therapies and bsAbs [11]. Of these agents, only two have received FDA approval and remain in routine clinical use: ofatumumab, a fully human mAb; and obinutuzumab, a humanized mAb [12,13]. Initially approved for refractory CLL, ofatumumab subsequently received additional indications in 2014, including first-line treatment in combination with chlorambucil and monotherapy for CLL refractory to both fludarabine and alemtuzumab [14]. Compared with first-generation rituximab, obinutuzumab exhibits enhanced antibody-dependent cellular cytotoxicity (ADCC), which contributes to its improved antitumor activity [15]. The improvement in progression-free survival observed with obinutuzumab compared with rituximab was first demonstrated in a large randomized phase III clinical trial involving 781 previously untreated patients with chronic lymphocytic leukemia (CLL). Patients receiving obinutuzumab plus chlorambucil achieved significantly longer progression-free survival than those treated with rituximab plus chlorambucil (*p* < 0.001), as well as higher complete response rates (20.7% vs. 7.0%) [16]. Similar findings were reported in a large randomized phase III trial that enrolled 1202 patients with follicular lymphoma. During the induction phase, patients received either obinutuzumab plus chemotherapy or rituximab plus chemotherapy, followed by maintenance therapy with the corresponding anti-CD20 antibody. Patients treated with obinutuzumab-based chemotherapy achieved significantly higher 3-year progression-free survival rates than those receiving rituximab-based chemotherapy (80.0% vs. 73.3%; *p* = 0.001) [17]. The safety profile of obinutuzumab was found to be comparable to that of rituximab, including in real-world clinical practice settings [18].

More recently, CD20 × CD3 bsAbs have emerged as a novel therapeutic class and are currently being evaluated against rituximab-based strategies in randomized clinical trials (NCT04408638, NCT06337318, NCT04663347, NCT06097364) [19,20,21,22].

### 2.2. Anti-CD52 mAbs

Alemtuzumab is currently the only commercially available anti-CD52 mAb. Following its FDA approval in 2001 for the treatment of patients with B-cell chronic lymphocytic leukemia (B-CLL) previously treated with alkylating agents and refractory to fludarabine therapy [23], alemtuzumab subsequently received an expanded indication in 2007 as monotherapy for B-CLL [24]. It has also received FDA Orphan Drug Designation for use as a lymphodepleting agent prior to treatment with genetically modified allogeneic T-cell product UCART22 [25].

Alemtuzumab also represents a promising therapeutic option for the management of T-cell prolymphocytic leukemia, and a phase Ib clinical trial evaluating its combination with the JAK1 inhibitor itacitinib is currently ongoing (NCT03989466) [26]. Current treatment guidelines recommend the use of alemtuzumab as a first-line therapy for T-cell prolymphocytic leukemia (T-PLL) [27,28].

Nevertheless, the clinical use of alemtuzumab remains limited by its toxicity profile, particularly the profound and prolonged lymphocyte depletion it induces, which increases the risk of opportunistic infections and necessitates appropriate anti-infective prophylaxis [29].

### 2.3. Anti-CD38 mAbs

Daratumumab was the first anti-CD38 mAb to receive FDA approval, initially being authorized in 2015 for the treatment of patients with MM who had received at least three prior lines of therapy (Figure 1) [30].

More recently, several phase III studies have demonstrated the benefits of daratumumab-containing quadruplet regimens as first-line therapy for newly diagnosed MM. The randomized phase III CEPHEUS trial (NCT03652064) demonstrated that the addition of daratumumab to bortezomib, lenalidomide, and dexamethasone (D-VRd) significantly improved the minimal residual disease (MRD) negativity rate compared with VRd alone (60.9% vs. 39.4%; *p* < 0.0001). Complete response rates (CR; 81.2% vs. 61.6%; *p* < 0.0001) and sustained MRD negativity for at least 12 months (48.7% vs. 26.3%; *p* < 0.0001) were also significantly increased [31].

Similarly, the randomized phase III CASSIOPEIA study (NCT02541383) showed that the addition of daratumumab to bortezomib, thalidomide, and dexamethasone significantly prolonged progression-free survival (PFS) in transplant-eligible patients with newly diagnosed MM (median not reached vs. 45.8 months; *p* < 0.0001) [32].

The therapeutic potential of daratumumab is also being explored in high-risk smoldering MM. The phase III AQUILA trial (NCT03301220) demonstrated that subcutaneous daratumumab prolonged PFS compared with active monitoring (63.1% vs. 40.8%) and was associated with improved overall survival (OS; 93.0% vs. 86.9%) [33].

Beyond its use in conventional combination regimens, daratumumab is investigated in combination with bsAbs. The phase III clinical trial NCT05083169 demonstrated that the combination of teclistamab and daratumumab significantly improved the estimated 36-month PFS compared with daratumumab alone (83.4% vs. 29.7%; *p* < 0.001). Complete response rates (81.8% vs. 32.1%), overall response rates (ORR; 89.0% vs. 75.3%), and MRD negativity rates (58.4% vs. 17.1%) were also significantly higher (*p* < 0.001) for all comparisons) [34].

The ongoing phase I TRIMM-2 study (NCT04108195) is evaluating the combination of talquetamab and daratumumab in R/R MM. Preliminary results have been encouraging, with ORR of 71.4% in the weekly (QW) cohort and 82.4% in the every-two-week (Q2W) cohort, with a median PFS of 23.3 and 21.2 months, respectively [35].

In parallel, additional anti-CD38 mAbs have been developed. Isatuximab became the second anti-CD38 mAb to receive FDA approval when it was authorized in 2020 in combination with pomalidomide and dexamethasone for patients with MM who had received at least two prior lines of therapy, including lenalidomide and a proteasome inhibitor [36]. In 2024, its indication was expanded to include first-line treatment of adults with newly diagnosed MM who are ineligible for autologous stem cell transplantation, following the results of the phase III study NCT03319667 [37]. This trial demonstrated significantly higher estimated 60-month PFS (63.2% vs. 45.2%; *p* < 0.001) and complete response rates (74.7% vs. 64.1%; *p* = 0.01) in the isatuximab-VRd group compared with the VRd group [38].

### 2.4. GPRC5D mAbs

SAR446523 is an investigational anti-GPRC5D mAb currently being evaluated in a phase I clinical trial (NCT06630806). Despite its early clinical development, it was granted FDA Orphan Drug Designation in 2025 for R/R MM [39].

### 2.5. Anti-CS1

Elotuzumab is a humanized immunostimulatory mAb that received FDA approval in 2015 for use in combination with lenalidomide and dexamethasone (ERd) in patients with MM who had received one to three prior lines of therapy. This approval was based on the results of the phase III ELOQUENT-2 trial, which demonstrated a 30% reduction in the risk of disease progression or death with ERd compared with lenalidomide and dexamethasone alone (*p* = 0.0004) [40,41].

In contrast to CD38 mAbs, there has been no expansion of indications into the first-line setting.

### 2.6. Anti-CD19 mAbs

Tafasitamab remains the only anti-CD19 humanized mAb to receive FDA approval, having been authorized in 2020 for the treatment of adults with R/R DLBCL [42]. Its indication has subsequently been expanded to include adults with R/R FL [43]. In addition, a phase III trial (NCT04824092) evaluating tafasitamab plus lenalidomide and R-CHOP compared with R-CHOP alone in patients with newly diagnosed high–intermediate- or high-risk DLBCL demonstrated improved PFS in the tafasitamab arm [44].

Inebilizumab (MEDI-551) is an anti-CD19 mAb engineered to enhance ADCC. However, its development in oncohematology was discontinued following phase I/II clinical evaluation in R/R advanced B-cell malignancies (NCT00983619) and an early phase I study in MM (NCT01861340) related to modest responses [45].

### 2.7. Anti-CD80 mAbs

To date, no anti-CD80 mAb has received FDA approval. Galiximab was evaluated in a phase II clinical trial (NCT00516217) involving patients with R/R HL, which was completed in 2015. It was also investigated in combination with rituximab in a phase II study of patients with stage II, III, or IV NHL. The study reported encouraging efficacy, with an ORR of 72.1% (95% confidence interval [CI], 59.2–82.9%), including a complete or unconfirmed complete response (CR/CRu) rate of 47.6% and a partial response rate of 24.6%, with a median PFS of 2.9 years [46]. Despite the initiation of two phase III clinical trials evaluating galiximab in combination with rituximab for follicular NHL (NCT00384150 and NCT00363636 completed in 2010), galiximab ultimately failed to achieve regulatory approval and was not further developed.

### 2.8. Anti-CD30 mAbs

To date, no unconjugated anti-CD30 mAb has received FDA approval. MDX-060 is the only agent to have undergone phase I/II clinical evaluation in patients with R/R CD30-positive lymphoma (NCT00059995 and NCT00284804). However, its activity as a monotherapy proved insufficient for further clinical development, with objective clinical responses observed in only six of the 72 treated patients [47].

Subsequent development efforts shifted toward the anti-CD30 ADC brentuximab vedotin, which ultimately achieved regulatory approval.

### 2.9. Anti-CD40 mAbs

To date, no anti-CD40 mAb has received FDA approval for use in oncohematology. Dacetuzumab provided initial clinical proof of concept for CD40-targeted therapy; however, its development was ultimately discontinued because of limited efficacy and dose-limiting toxicities (hepatotoxicity, CRS, thrombocytopenia, general hyperimmune stimulation, and tumor angiogenesis in response to endothelial cell activation) [48].

### 2.10. Anti-CD74 mAbs

The anti-CD74 mAb milatuzumab received FDA Orphan Drug Designation for the treatment of MM in 2008 and was subsequently evaluated in a phase I/II clinical trial involving frail patients with refractory CLL. However, its clinical development was ultimately discontinued due to lack of efficacy (NCT01101594) [49,50].

### 2.11. Anti-CD33 mAbs

The only anti-CD33 mAb currently under clinical investigation in AML (NCT02575963) and MM (NCT02998047) is lintuzumab radiolabeled with actinium-225. Results from a phase I study evaluating [^225^Ac]Ac-lintuzumab combined with CLAG-M (cladribine + cytarabine + G-CSF + mitoxantrone) salvage therapy in R/R AML showed a composite complete remission (CRc) rate (CR/CRi) of 56.6% overall, with MRD negativity achieved in 8 of 12 responders [51].

### 2.12. Anti-CD22 mAb

Only one anti-CD22 mAb (epratuzumab) has progressed to phase II clinical development, intended for use in combination with rituximab (NCT00553501) [52]. However, it showed limited clinical efficacy, and its development was subsequently discontinued [53].

### 2.13. Monoclonal Antibody Overview

The first anti-CD20 monoclonal antibody, rituximab, remains the standard of care for many CD20-positive B-cell lymphomas, including diffuse large B-cell lymphoma (DLBCL) and follicular lymphoma (FL) [54]. In contrast, its use in chronic lymphocytic leukemia (CLL) has declined with the emergence of tyrosine kinase inhibitors [55]. Numerous other anti-CD20 monoclonal antibodies have entered clinical development; however, obinutuzumab is the only agent that has demonstrated significantly superior clinical activity compared with rituximab [56].

Among the targets explored for the management of multiple myeloma (MM), CD38 has been the most successful, leading to the commercialization of daratumumab, the first anti-CD38 monoclonal antibody. Daratumumab demonstrated a significant improvement in complete response rates when combined with chemotherapy in MM. More recent studies have reported comparable outcomes with chemotherapy-free approaches based on combinations including bispecific antibodies such as teclistamab and talquetamab. Daratumumab is now incorporated into international clinical practice guidelines for multiple myeloma, including frontline treatment regimens. Isatuximab has also shown favorable clinical results but is generally used after daratumumab in routine practice, with daratumumab remaining the preferred anti-CD38 monoclonal antibody [57]. Monoclonal antibodies targeting GPRC5D, CS1, CD74, and CD33 in MM have not demonstrated sufficiently convincing efficacy in the clinical trials discussed above and are therefore not included in current treatment recommendations.

Alemtuzumab, the only commercially available anti-CD52 monoclonal antibody, is rarely used in current clinical practice and is mainly reserved for selected cases of T-cell prolymphocytic leukemia (T-PLL) [28].

Targets other than CD20 have also been explored in lymphoma, with therapeutic strategies varying according to the lymphoma subtype.

In DLBCL, tafasitamab—an anti-CD19 monoclonal antibody—has been incorporated into clinical practice guidelines as a preferred treatment option, in combination with lenalidomide, for patients with relapsed or refractory DLBCL who are not eligible for autologous stem cell transplantation [58]. Recent guidelines continue to support the use of tafasitamab, particularly in the management of B-cell lymphomas [59]. Despite proof-of-concept activity of the anti-CD40 monoclonal antibody dacetuzumab in DLBCL, its clinical development was discontinued because of an unfavorable benefit–risk profile [48].

In non-Hodgkin lymphoma (NHL), galiximab—a monoclonal antibody targeting CD80—initially appeared promising; however, its development was discontinued during Phase III trials because of recruitment difficulties associated with evolving standards of care [60]. Similarly, despite reaching Phase II clinical development, an anti-CD22 monoclonal antibody failed to demonstrate sufficient efficacy, leading to the discontinuation of its development [53].

## 3. Bispecific Antibodies and Bispecific T-Cell Engagers

**Figure 2:**
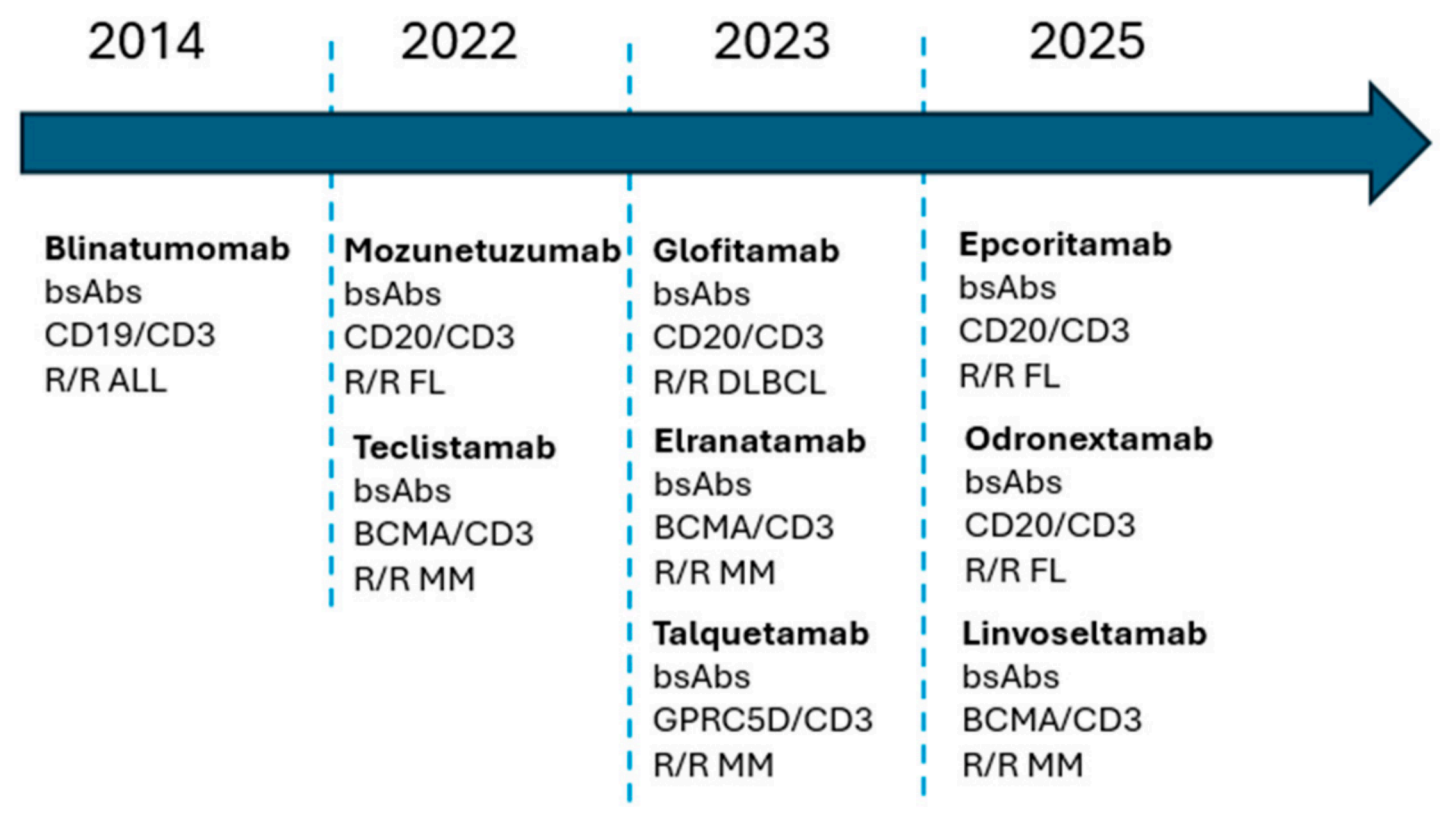
History of FDA-approved bsAbs.

Bispecific T-cell engagers used in oncohematology are designed to redirect T lymphocytes toward tumor cells, primarily activating them via CD3 engagement. They typically target CD3 on T cells and a tumor-associated antigen specific to the underlying hematologic malignancy, such as B-cell maturation antigen (BCMA) in MM, CD19 in precursor B-cell acute lymphoblastic leukemia (B-ALL), CD20 in lymphomas, or CD123 in AML. Over the past decade, nine bsAbs have received FDA approval, mainly for the treatment of MM, FL, and ALL (Figure 2).

### 3.1. CD19 × CD3 bsAbs

Blinatumomab is a CD19 × CD3 bsAb that received FDA approval in 2014 for the treatment of Philadelphia chromosome-negative R/R precursor B-ALL [61]. In 2024, the FDA expanded its indication to include adult and pediatric patients aged one month and older with CD19-positive, Philadelphia chromosome-negative precursor B-ALL during the consolidation phase of multiphase chemotherapy [62]. This expanded indication was based on the results of four phase III clinical trials including a large cohort of adult patients (Table 2). Overall survival was significantly improved when blinatumomab was combined with consolidation chemotherapy compared with consolidation chemotherapy alone (*p* = 0.002) in a large cohort of 488 adult patients with BCR::ABL1-negative BCP-ALL who had MRD-negative remission after induction and intensification chemotherapy [63]. In pediatric patients aged 28 days to 18 years, blinatumomab demonstrated a significant improvement in PFS compared with chemotherapy administered during the third consolidation cycle following induction and two cycles of consolidation chemotherapy in patients with high-risk, first-relapse, Philadelphia chromosome-negative (Ph–) B-cell precursor ALL [64]. Similarly, favorable results have been reported in patients presenting with bone marrow and/or extramedullary relapse [65].

More recently, several studies have explored the combination of blinatumomab with tyrosine kinase inhibitors (TKIs) as part of chemotherapy-free treatment strategies (Table 2). The combination of dasatinib and blinatumomab as an induction/consolidation regimen induced durable hematologic responses in adults with Philadelphia chromosome-positive ALL (NCT02744768) [66]. Likewise, the ponatinib–blinatumomab combination demonstrated favorable efficacy and safety, reducing the need for intensive chemotherapy and HSCT during first remission in most patients (NCT03263572) [67]. These two-phase II clinical trials were conducted without a comparator in a cohort of 63 and 60 patients. However, the Blinatumomab + Ponatinib combination achieved superior outcomes compared with conventional TKI-plus-chemotherapy regimens in a phase III clinical trial conducted in a cohort of 158 patients (Table 2) [68].

Although most studies have focused on B-ALL, a phase I clinical trial (NCT02568553) also evaluated the combination of blinatumomab and lenalidomide in R/R B-cell non-Hodgkin lymphoma (B-NHL), with the aim of mitigating blinatumomab-induced T-cell exhaustion (Table 2) [69].

AFM11, another CD19 × CD3 bsAb, also entered phase I clinical evaluation. However, neurotoxicity and limited efficacy observed in two phase I trials (NCT02106091 and NCT02848911) ultimately halted its clinical development [70].

**Table 2:** Summary of recent clinical trial for CD19 × CD3 bsAbs in B-ALL and R/R B-NHL.

Agent	Target	Year (Estimated Completion Date)	Phase	Cases	OS Rate	CR/CRi (%)	Median Survival (Months)	Clinical Trial	Ref.
B-ALL									
Blinatumomab + chemotherapy vs. chemotherapy	CD19 × CD3	2027	III	488	85% vs. 68% *p* = 0.002	NA	NA	NCT02003222	[63]
Blinatumomab vs. chemotherapy	CD19 × CD3	2022	III	108	85.2% vs. 70.4%	NA	22.4 months EFS: 69% vs. 43% *p* < 0.001	NCT02393859	[64,71]
Blinatumomab + chemotherapy vs. chemotherapy	CD19 × CD3	2026	III	255	97.1% vs. 84.8% *p* = 0.020 ^(1)^	NA	NA	NCT02101853	[65]
	CD19 × CD3	2027	III	1440	3-year disease-free survival 96.0 vs. 87.9% *p* < 0.001	NA	NA	NCT03914625	[72]
Blinatumomab + dasatinib	CD19 × CD3 + TKI	2021	II	63	80.7%	93.1%	NA	NCT02744768	[66]
Blinatumomab + Ponatinib	CD19 × CD3 + TKI	2027	II	60	3-year OS rate: 91%	83%	NA	NCT03263572	[67]
Blinatumomab + Ponatinib vs. imatinib	CD19 × CD3 + TKI	2027	III	158	NA	94.4% vs. 79.4% *p* = 0.001	18 months EFS ^(2)^: 89.9% vs. 76.8% *p* = 0.011	NCT04722848	[68]
R/R B-NHL									
Blinatumomab + lenalidomide	CD19 × CD3 + Chemotherapy	2025	I	35	NA	70%	NA	NCT02568553	[69]

^(1)^ 4-year OS For bone marrow (BM) ± extramedullary (EM) relapses; ^(2)^ event-free survival (EFS).

### 3.2. CD20 × CD3 bsAbs

#### 3.2.1. Mosunetuzumab

Mosunetuzumab became the first bsAb to receive FDA approval for the treatment of FL in 2022 [73]. Long-term follow-up from the pivotal phase I/II study (NCT02500407) confirmed the durability of its clinical activity, with sustained responses observed in patients with R/R FL after at least two prior lines of therapy (Table 2) [74].

Subcutaneous administration demonstrated efficacy comparable to the intravenous formulation while reducing both the incidence (29.8% vs. 44.4%) and severity (grade ≥ 2: 9.6% vs. 18.9%) of CRS, thereby improving treatment convenience and facilitating outpatient management [75].

Beyond FL, mosunetuzumab has also demonstrated durable clinical activity in other B-NHL, extending its potential therapeutic utility (Table 3) [76]. The main limitation of these studies is that they were conducted without a comparator arm. Phase III clinical trials with comparator arms are currently underway, including studies comparing rituximab vs. mosunetuzumab (NCT06337318) and mosunetuzumab plus lenalidomide vs. rituximab plus lenalidomide (NCT04712097); however, the results have not yet been published. In clinical practice, it is recommended in combination with polatuzumab vedotin as a second-line therapy option for patients with relapsed FL occurring more than 12 months after first-line therapy who are not candidates for transplantation, according to NCCN guidelines [59].

#### 3.2.2. Glofitamab

Glofitamab received FDA approval in 2023 for the treatment of R/R DLBCL. In 2025, its indication was expanded to include use in combination with gemcitabine and oxaliplatin (GemOx) for selected patients with R/R DLBCL, following the results of the STARGLO study (NCT04408638) including transplant-ineligible patients (aged ≥18 years) with histologically confirmed R/R DLBCL after one or more previous therapies which demonstrated significantly improved OS with glofitamab plus GemOx compared with Rituximab plus GemOx (*p* = 0.011) (Table 4) [19].

Glofitamab is also being investigated in combination with ADCs for the treatment of aggressive lymphomas. The combination of glofitamab and loncastuximab tesirine, a CD19-directed ADC (NCT04970901), has shown encouraging preliminary activity in patients with R/R aggressive B-NHL (aged ≥18 years) (Table 4) [77]. This study comprises multiple treatment arms designed to assess loncastuximab in combination with various antibodies, including polatuzumab vedotin, glofitamab, mosunetuzumab, and obinutuzumab. To date, only preliminary results have been reported.

Similarly, the combination of glofitamab and polatuzumab vedotin (NCT04914741; NCT03533283) in adult patients (Age ≥ 18 years) with high-risk (HR) LBCL in phase I/II trials with no comparator arm demonstrated high response rates and durable remissions while maintaining a manageable safety profile in heavily pretreated patients [78,79].

In addition, glofitamab is explored as a treatment option for R/R lymphomas from 1 month after CAR T-cell therapy in adult patients (aged ≥18 years). Preliminary results in a phase II clinical trial without a comparator in a cohort of 46 patients suggest favorable OS outcomes together with an acceptable safety profile in this setting (NCT04703686) (Table 4) [80]. In clinical practice, glofitamab is recommended for R/R DLBCL in combination with gemcitabine and oxaliplatin in patients with early progression of disease (POD) who are unsuitable for CAR T therapy, or in those with late POD who are unsuitable for platinum-based reinduction therapy and autologous stem cell transplantation (ASCT). Due to the increased risk of adverse events and cytokine release syndrome (CRS), all patients must receive premedication with obinutuzumab [81,82].

**Table 4:** Summary of recent clinical trial for CD20 × CD3 bsAbs glofitamab in R/R DLBCL and LBCL.

Agent	Target	Year (Estimated Completion Date)	Phase	Cases	OS Rate	CR/CRi (%)	Median Survival (Months)	Clinical Trial	Ref.
DLBCL									
Glofitamab + gemOx vs. rituximab + gemOx	CD20 × CD3 + Chemotherapy	2028	III	274	13.8 months vs. 9.0 months (*p* = 0.011)	NA	NA	NCT04408638	[19]
Glofitamab+ obinutuzumab	CD20 × CD3 + CD20	2029	I/II	155	NA	39%	NA	NCT03075696 [82]	
Glofitamab + Loncastuximab Tesirine	CD20 × CD3 + ADC CD19	2027	Ib	31	NA	64.5%	NA	NCT04970901	[77]
Glofitamab After failing CAR T-cell Therapy	CD20 × CD3	2025	II	46	OS: 14.7 months	NA	NA	NCT04703686	[80]
LBCL									
Glofitamab + R-CHOP or Glofitamab + polatuzumab vedotin + RCHP	CD20 × CD3 + CD20 mAbs + chemotherapy or ADC CD79b + CD20 mAbs + chemotherapy	2025	I/II	80	86% vs. 92%	100% vs. 98%	NA	NCT04914741	[79]
Glofitamab + polatuzumab vedotin	CD20 × CD3 + ADC CD79b	2026	I/II	129	OS: 33.8 months	59.7%	NA	NCT03533283	[78]

#### 3.2.3. Epcoritamab

Epcoritamab was approved by the FDA in 2025, in combination with lenalidomide and rituximab for R/R FL [83], following the results of the phase III EPCORE FL-1 study (NCT05409066) conducted in adult patients with a comparator arm that demonstrated a significantly higher complete response (CR) rate in the epcoritamab + lenalidomide + rituximab arm compared with the control arm—rituximab plus lenalidomide (Table 5) [84]. In clinical practice, it is recommended for relapsed/refractory (R/R) DLBCL and follicular lymphoma (FL) after ≥2 prior systemic therapy regimens [81].

#### 3.2.4. Odronextamab

Odronextamab received FDA approval in 2025 for the treatment of R/R FL after two or more lines of systemic therapy. It has also been approved by the European Medicines Agency (EMA) for the treatment of R/R FL and DLBCL [85]. Clinical activity has been demonstrated both with odronextamab monotherapy (NCT03888105) and in combination with CHOP chemotherapy [86]. In the latter setting, odronextamab plus CHOP was superior to the standard R-CHOP regimen in patients with previously untreated DLBCL (NCT06091865) (Table 6) [87].

As with other CD20 × CD3 bsAbs, odronextamab has also been investigated in patients with R/R DLBCL who progressed after CAR T-cell therapy. A phase I study (NCT02290951) reported encouraging clinical activity together with an acceptable safety profile (Table 6) [88]. A phase III randomized clinical trial is currently ongoing with a comparator arm to evaluate the efficacy and safety of odronextamab plus lenalidomide compared with rituximab plus lenalidomide in patients with NHL (NCT06149286). Odronextamab has received FDA and EMA regulatory approvals but has not yet been incorporated into the B-Cell Lymphomas NCCN Clinical Practice Guidelines.

#### 3.2.5. Plamotamab

The clinical development of plamotamab was initiated in a phase I clinical trial (NCT02924402) evaluating subcutaneous administration in heavily pretreated patients with R/R NHL who had previously received CAR T-cell therapy, as well as in a phase II trial in combination with tafasitamab (NCT05328102). However, its development in oncohematology currently appears to have been discontinued due to a business decision [89,90].

### 3.3. BCMA × CD3

#### 3.3.1. Teclistamab

Teclistamab received FDA approval in 2022 for the treatment of adults with R/R MM who had received at least four prior lines of therapy [91]. Clinical development is now focusing on earlier treatment settings. In March 2026, the FDA expanded its indication to include use in combination with subcutaneous daratumumab (daratumumab and hyaluronidase-fihj) for adults with R/R MM who had received at least one prior line of therapy, including a proteasome inhibitor and an immunomodulatory agent [92]. An application has also been submitted to the EMA seeking approval of teclistamab monotherapy for patients with R/R MM after at least one prior line of therapy [93]. In the phase III trial NCT05083169, the combination of teclistamab and subcutaneous daratumumab significantly improved PFS compared with standard daratumumab-based therapy in patients with R/R MM (Table 7) [34].

Teclistamab is also being evaluated in combination with lenalidomide, with preliminary results demonstrating deep and durable responses together with high rates of MRD negativity (Table 7) [94]. It is listed as an emerging option in the MM NCCN 2025 Version 2 Guidelines, in addition to quadruplet therapies for the management of MM [95].

#### 3.3.2. Linvoseltamab

Following the success of teclistamab, linvoseltamab received regulatory approval from both the FDA and the EMA in 2025 as monotherapy for adults with R/R MM who had received at least three prior lines of therapy. This approval was supported by the results of the pivotal LINKER-MM1 study (NCT03761108) (Table 8) [96,97,98]. Ongoing phase III studies are now investigating linvoseltamab, both in combination with elotuzumab (NCT05730036) and in earlier lines of therapy (NCT07222761). Several phase III clinical trials with active comparator arms are currently ongoing, including studies comparing linvoseltamab with elotuzumab plus pomalidomide and dexamethasone (EPd) (NCT05730036) [99], or daratumumab, lenalidomide, and dexamethasone plus linvoseltamab vs. daratumumab, lenalidomide, and dexamethasone (NCT06932562). However, the results have not yet been published. Linvoseltamab is now being incorporated into clinical practice guidelines for the treatment of relapsed/refractory multiple myeloma (R/R MM) in patients who have received at least four prior lines of therapy [100].

#### 3.3.3. Elranatamab

In 2023, elranatamab received regulatory approval from both the FDA and the EMA for the treatment of adults with R/R MM who had received at least three prior lines of therapy, based on the results of the phase II MagnetisMM-3 study (NCT04649359) (Table 9) [101,102,103].

An ongoing phase II trial is evaluating elranatamab earlier in the treatment course for patients with R/R MM who have received one to three prior lines of therapy (NCT06711705). Two phase III trials are further exploring elranatamab-based combinations: one with daratumumab (NCT05020236; MagnetisMM-5) and another with daratumumab plus lenalidomide (NCT05623020; MagnetisMM-6). A phase III clinical trial with a comparator arm is currently underway to confirm the results of the phase II single-arm study. The trial is evaluating elranatamab monotherapy vs. elranatamab plus daratumumab (NCT05020236). Elranatamab is now included in international clinical practice guidelines, together with linvoseltamab and teclistamab, as a treatment option for patients with relapsed/refractory multiple myeloma after four or more prior lines of therapy [100].

#### 3.3.4. Alnuctamab

Alnuctamab demonstrated promising efficacy in a phase I clinical study (NCT03486067), with an ORR of 58.9% among all R/R MM patients treated with subcutaneous alnuctamab, and a MRD negativity rate of 49.5% [104]. A phase III clinical trial was initiated in May 2024 but subsequently withdrawn for change in Business objectives (NCT06232707) [105].

#### 3.3.5. Etentamig

Etentamig (ABBV-383) has been evaluated in a phase I study involving patients with R/R MM (NCT03933735) (Table 10). Initial results demonstrated encouraging clinical activity, warranting further investigation [106]. Clinical development remains ongoing, with several phase I/II studies (NCT05259839; NCT06223516; NCT05650632; NCT06892522) and a phase III trial (NCT06158841) currently evaluating ABBV-383 monotherapy compared with standard-of-care treatment in patients with R/R MM.

### 3.4. CD38/CD3

No CD38 × CD3 bsAb has currently been approved by the FDA. Their potential has been demonstrated in preclinical studies [107]; however, only one compound, ISB-1342, entered phase I clinical development in previously treated MM patients (NCT0330911) [108]. No further clinical progress has been reported since 2023.

### 3.5. GPRC5D × CD3

Talquetamab was approved by both the FDA and the EMA in 2023 for the treatment of adults with R/R MM who had received at least four prior lines of therapy, based on the results of the phase II MonumenTAL-1 study (NCT04634552) [109,110]. A phase I/II study evaluated the combination of talquetamab and teclistamab (NCT04586426). Although preliminary efficacy was observed, the regimen was associated with a high incidence of grade 3 or 4 adverse events, which occurred in 96% of treated patients [111]. The phase II TRIMM-2 study (NCT04108195) is also investigating talquetamab in combination with daratumumab for the treatment of R/R MM (Table 11) [35]. The results of ongoing phase III randomized clinical trials with active comparator arms are awaited to confirm these findings (NCT06208150, NCT05461209). It appears in the latest ASCO 2026 guidelines as a salvage treatment option for patients with MM at the fourth or later relapse [100].

### 3.6. FcRH5 × CD3

Cevostamab demonstrated encouraging clinical activity in a phase I study involving patients with heavily pretreated R/R MM (Table 12). The safety profile was considered manageable, with immune effector cell-associated neurotoxicity syndrome (ICANS), infections, and CRS occurring in 13.2%, 53.9%, and 74.3% of patients, respectively (NCT03275103) [112]. These encouraging early-phase results have supported the continued clinical development of cevostamab. A phase III trial (CEVOLUTION; NCT07555938) is currently evaluating cevostamab in combination with pomalidomide and dexamethasone compared with standard-of-care treatment in patients with previously treated MM. Furthermore, cevostamab has been recognized as a promising therapeutic agent in MM, as highlighted at the 2024 ASH Annual Meeting [113].

### 3.7. CD123 × CD3

Flotetuzumab entered phase I/II clinical development as salvage immunotherapy for refractory AML, including patients with primary induction failure or early relapse (<6 months) (NCT02152956) (Table 13). Early results suggested clinical activity with a manageable safety profile, with the most frequent adverse events being grade 1–2 CRS [115]. A phase I study is currently ongoing in pediatric patients with R/R AML (NCT04158739).

Vibecotamab (XmAb14045) was assessed in a phase I study in patients with R/R AML (Table 13) [116]. A phase II trial is currently ongoing in patients with MRD-positive AML and myelodysplastic syndromes (MDSs) following hypomethylating agent failure; however, results have not yet been published (NCT05285813).

JNJ-63709178 was evaluated in a phase I study in patients with R/R AML (NCT02715011). However, its clinical development was discontinued due to limited efficacy and tolerability concerns [117].

### 3.8. Other bsAb Targets for AML

One CD33 × CD3 (vixtimotamab) and one CLEC12A × CD3 (MCLA-117) bispecific antibody entered phase I clinical development (NCT03144245 and NCT05817058, respectively); however, no additional studies are currently ongoing. A BCMA × CD16A-targeting compound (AFM28) demonstrated preclinical efficacy; however, its development was discontinued during phase I clinical evaluation (NCT05817058) [118].

### 3.9. Overview of bsAbs

Blinatumomab is the only bispecific CD19 × CD3 monoclonal antibody that has demonstrated a significant improvement in OS and MS in R/R ALL. The positive results of the Phase III study evaluating the combination of blinatumomab and ponatinib support a chemotherapy-free targeted immunotherapeutic strategy for adults with Philadelphia chromosome-positive (Ph+) ALL [68]. It is incorporated into current treatment guidelines for B-cell acute lymphoblastic leukemia (B-ALL) [119,120].

In the management of MM, four bispecific antibodies are currently included in clinical practice guidelines for the treatment of R/R MM after at least four prior lines of therapy. Three target BCMA × CD3 (teclistamab, linvoseltamab, and elranatamab), while one targets GPRC5D × CD3 (talquetamab). Among these agents, only teclistamab has been evaluated in a randomized phase III clinical trial with an active comparator arm (teclistamab plus daratumumab vs. daratumumab plus dexamethasone). Published results demonstrated a significant improvement in PFS. The other BCMA × CD3 bispecific antibodies, including alnuctamab and etentamig, are still undergoing clinical evaluation and are not currently included in MM treatment guidelines.

Although the results of the phase III trial evaluating cevostamab vs. standard-of-care treatment have not yet been published, cevostamab represents the first promising FcRH5 × CD3 bispecific antibody in the treatment of multiple myeloma.

In lymphoma, the bispecific antibodies that have been successfully developed all target CD20 × CD3. Mosunetuzumab and epcoritamab have received regulatory approval and are included in clinical practice guidelines for the management of FL. However, to date, only epcoritamab has demonstrated a statistically significant improvement in ORR in a phase III trial with a comparator arm (epcoritamab plus lenalidomide and rituximab vs. lenalidomide and rituximab).

In DLBCL, two CD20 × CD3 bispecific antibodies have been developed: glofitamab and odronextamab. However, only glofitamab has demonstrated an improvement in PFS in a comparative clinical trial when combined with gemcitabine, compared with rituximab plus gemcitabine. Following regulatory approval in 2023, glofitamab has been incorporated into current treatment guidelines for R/R DLBCL in combination with gemcitabine and oxaliplatin. Odronextamab is a more recent agent, has not yet demonstrated significant efficacy vs. a comparator arm, and is therefore not currently included in clinical practice guidelines.

In AML, only two CD123 × CD3-targeting bispecific antibodies, flotetuzumab and vibecotamab, have shown potential clinical interest. However, their ORRs remain modest (approximately 30% and 9%, respectively). Clinical development is still at an early stage (Phase I/II), and neither agent has yet been evaluated against a standard-of-care regimen or within a randomized controlled trial with a comparator arm.

## 4. Trispecific Antibodies

The development of mAbs has evolved to include trispecific antibodies (TsAbs), which are designed to simultaneously target an activating receptor on immune effector cells (CD3 for T-cell recruitment or CD16a on natural killer [NK] cells), a tumor-associated antigen (e.g., CD38 in MM), and a third target. This third component may consist either of an additional tumor-associated antigen—to increase tumor specificity and improve clinical efficacy—or of a costimulatory receptor expressed on immune cells, such as CD28, to further enhance effector functions [121].

### 4.1. TsAb

SAR442257 (SAR257) is a TsAb targeting CD38, a validated target in MM and NHL; together with CD3, a component of the T-cell receptor complex; and CD28, a T-cell costimulatory receptor. It was evaluated in a phase I study involving patients with R/R MM and NHL (NCT04401020) (Table 14) [122]. Treatment-emergent adverse events occurred in 68% of patients, including grade ≥ 3 events in 21.3%. Therefore, the study was discontinued during dose escalation because of an unfavorable benefit–risk profile [122].

Ramantamig (JNJ-79635322) is a TsAb targeting BCMA and GPRC5D, two validated targets in MM, together with CD3. Preclinical studies demonstrated potent T-cell-mediated cytotoxicity against MM cells expressing BCMA, GPRC5D, or both—findings that were subsequently confirmed in vivo using xenograft models [123]. Two phase I clinical trials are currently ongoing (NCT05652335 and NCT06768489) (Table 14).

IBI3003—another TsAb targeting BCMA, GPRC5D, and CD3—is currently being evaluated in a phase I study in patients with R/R MM. Preliminary results suggest encouraging clinical activity together with a manageable safety profile, with adverse events occurring in 48.7% of patients, including grade ≥ 3 events in 28.2% (Table 14) [124].

JNJ-80948543 is a TsAb targeting CD79b and CD20—two antigens expressed on lymphoma cells—together with CD3. Preclinical studies demonstrated potent T-cell-mediated cytotoxicity against CD79b^+^ and/or CD20^+^ cells, findings that were subsequently confirmed in xenograft models [125]. Two phase I clinical trials are currently ongoing in patients with NHL (NCT05424822, NCT06139406).

### 4.2. Trispecific Killer Engager (TriKE)

Additional trispecific NK-cell engagers have been developed to harness the cytotoxic activity of NK cells through CD16-mediated activation [121]. Proof of concept was established with the first-generation TriKE 161533 (GTB-3550), which simultaneously targets CD16, an activating receptor expressed on mature NK cells; interleukin-15 (IL-15) to enhance NK-cell proliferation and function; and CD33, a validated target in AML. Preclinical studies demonstrated greater NK-cell expansion and enhanced antitumor activity compared with the first-generation bispecific NK-cell engager 1633BiKE (CD16 × CD33). GTB-3550 subsequently entered phase I clinical development for the treatment of R/R AML and high-risk MDS (NCT03214666) (Table 15) [126].

GTB-3650 is a second-generation TriKE that also targets CD16, CD33, and IL-15 and was engineered to improve the pharmacokinetic profile and antitumor efficacy of GTB-3550. Preliminary results from an ongoing phase I study (NCT06594445) showed that two of the first four enrolled patients with AML achieved stable disease, with no treatment-emergent adverse events or dose-limiting toxicities reported [127].

**Table 15:** Summary of recent clinical trial for TriKE in AML and MDS.

Agent	Target	Year (Estimated Completion Date)	Phase	Indication	Cases	OS Rate (%)	CR/CRi (%)	Median Survival (Months)	Clinical Trial	Ref.
GTB-3550	CD16, IL-15, CD33	2021	I/II	AML, MDS	11	OS: 45.4%	NA	NA	NCT03214666	[128]

## 5. Antibody-Drug Conjugates

**Figure 3:**
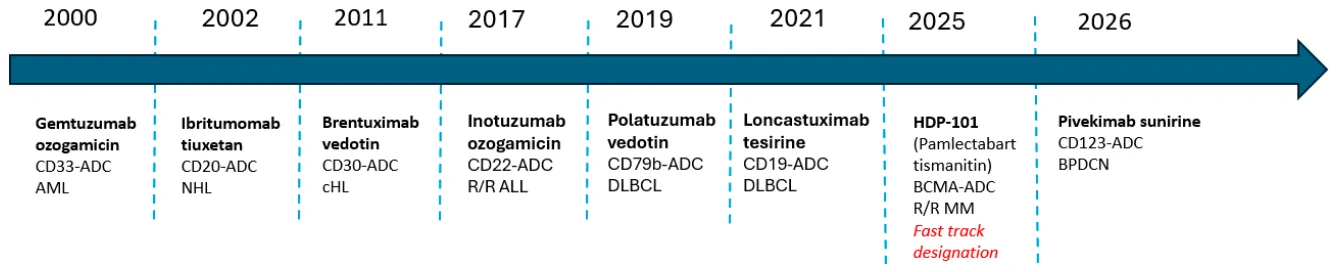
History of FDA-approved ADCs.

ADCs are composed of three parts: an mAb directed against a tumor-associated antigen, a cytotoxic payload, and a linker connecting the two. This design allows selective delivery of highly potent cytotoxic agents to malignant cells while limiting systemic toxicity. Three major classes of antibody conjugates have been developed, depending on the nature of the payload: cytotoxic drugs, radionuclides, or toxins [129]. The development of ADCs in oncohematology has expanded considerably over the past two decades and has profoundly transformed the therapeutic landscape of hematologic malignancies (Figure 3). The following section reviews currently approved ADCs as well as emerging therapeutic strategies.

### 5.1. Anti-CD33 ADCs

Gemtuzumab ozogamicin (GO) is an ADC composed of the anti-CD33 mAb gemtuzumab linked to the cytotoxic agent N-acetyl-γ-calicheamicin. It was initially approved by the FDA in 2000 for the treatment of patients aged 60 years or older with CD33-positive AML in first relapse who were not considered candidates for intensive chemotherapy [130]. However, this approval was voluntarily withdrawn in 2010 following the results of the SWOG S0106 study (NCT00085709), which showed no significant clinical benefit from the addition of GO to standard induction therapy with daunorubicin and cytarabine. Complete remission rates were similar between treatment groups (69% vs. 70%; *p* = 0.559), whereas grade 4 nonhematologic adverse events occurred more frequently in the GO arm (21% vs. 12%) (Table 16) [131]. Nevertheless, GO was reapproved by the FDA in 2017 using a fractionated dosing schedule for the treatment of R/R CD33-positive AML in patients aged 2 years and older, following the results of the ALFA-0701 study (NCT00927498), which demonstrated a favorable benefit–risk profile when GO was combined with daunorubicin and cytarabine [130,132]. In June 2020, the FDA further expanded the indication to include newly diagnosed CD33-positive AML in pediatric patients aged 1 month and older, based on the results of study NCT01407757 [133,134]. GO has since become an important therapeutic option in the management of AML, particularly for selected high-risk patient populations [133,135].

IMGN779 is an ADC composed of an anti-CD33 mAb conjugated to the DNA-alkylating payload DGN462. Preclinical studies using multiple AML xenograft models demonstrated significant tumor regression and prolonged survival, supporting its subsequent evaluation in a phase I clinical trial involving adults with R/R CD33-positive AML (NCT02674763) [136]. According to the NCCN Guidelines, gemtuzumab ozogamicin (GO) may be incorporated into conventional induction chemotherapy regimens in several treatment settings, including intensive remission induction therapy for patients with favorable-risk cytogenetics and certain patients with intermediate- or poor-risk acute myeloid leukemia (AML) [137].

### 5.2. Anti-CD20 ADCs

Ibritumomab tiuxetan is a radioimmunoconjugate composed of ibritumomab, an anti-CD20 mAb, linked to the chelator tiuxetan, which requires extemporaneous radiolabeling with yttrium-90 [138]. It was approved by the FDA in 2002 for the treatment of NHL and subsequently by the EMA in 2004. However, its use has become limited because of the logistical constraints associated with radiolabeling and the emergence of alternative therapeutic options for NHL. It was ultimately withdrawn from the European market in 2024 and is no longer part of current clinical practice [139,140].

### 5.3. Anti-CD30 ADCs

Brentuximab vedotin is an ADC composed of brentuximab, an anti-CD30 mAb, conjugated to the microtubule-disrupting agent monomethyl auristatin (MMA) E. Since its initial FDA approval in 2011 for the treatment of relapsed classical HL [141], its indications have expanded considerably. In 2015, it was approved as consolidation therapy following autologous HSCT for patients with HL at high risk of relapse or progression, based on the results of the AETHERA study which demonstrated a significant improvement in 5-year progression-free survival compared with placebo ([HR], 0.521; 95% CI, 0.379–0.717) (NCT01100502) [142,143]. In 2018, approval was extended to first-line treatment of anaplastic large-cell lymphoma and other CD30-positive peripheral T-cell lymphomas in combination with chemotherapy based on the study NCT01777152, which demonstrated a significant improvement in 5-year progression-free survival compared with placebo (HR = 0.68; 95% CI, 0.53 to 0.86) [144,145,146]. More recently, in 2025, the FDA further expanded its indication to include combination therapy with lenalidomide and rituximab for adults with R/R DLBCL who had received at least two prior lines of systemic therapy and were not eligible for autologous HSCT or CAR T-cell therapy based on the study NCT04404283, which demonstrated a significant improvement of median OS, ORR and Median PFS vs. placebo arm (Table 17) [147,148]. Brentuximab vedotin was also approved in 2022 in combination with chemotherapy as first-line treatment for pediatric patients aged 2 years or older with high-risk HL based on the study NCT02166463, which demonstrated a significant improvement of 3-year EFS vs. standard of care [149,150].

Several clinical trials are currently investigating brentuximab vedotin in combination with chemotherapy or immune checkpoint inhibitors such as nivolumab, reflecting the continuing expansion of its therapeutic applications (NCT01716806 [151], NCT03907488 [152], NCT03113500 [153]). Current guidelines include brentuximab vedotin plus AVD (BV + AVD) for patients with stage I–II Hodgkin lymphoma without risk factors, as well as a therapeutic option for patients with stage I–II unfavorable disease and stage III–IV disease [154].

**Table 17:** Summary of recent clinical trial for Brentuximab vedotin in HL, CD30-positive Mature T-cell Lymphomas and DLBCL.

Agent	Target	Year (Estimated Completion Date)	Phase	Indication	Cases	OS Rate (%)	CR/CRi (%)	Median Survival (Months)	Clinical Trial	Ref.
HL										
Brentuximab vedotin vs. placebo plus best supportive care alone	CD30	2020	III	R/R HL consolidation after auto-HSCT	329	NA	NA	The 5-year PFS rate: 59% vs. 41% HR = 0.521; 95% CI, 0.379–0.717	NCT01100502	[143]
Brentuximab vedotin (BV) + AVD vs. ABVD ^(1)^	CD30 + chemotherapy	2026	III	Stage III or IV HL	1334	6-year OS 93.9% and 89.4% HR = 0.68; 95% CI, 0.53 to 0.86	NA	NA	NCT01712490	[146]
BV + doxorubicin + vincristine + etoposide + cyclophosphamide + prednisone vs. standard-care	CD30 + chemotherapy	2026	III	Pediatric HR HL	587	NA	NA	3-year EFS: 92.1% vs. 82.5% *p* < 0.001	NCT02166463	[150]
CD30-positive peripheral T-cell lymphomas										
Brentuximab vedotin + CHP vs. CHOP	CD30 + chemotherapy	2020	III	untreated CD30-positive peripheral T-cell lymphomas	601	NA	NA	Median PFS: 48.2 months vs. 20.8 months (*p* = 0.0110)	NCT01777152	[155]
DLBCL										
Brentuximab vedotin + lenalidomide (len) + rituximab (R) vs. placebo + len + R	CD30 + CD20 + chemotherapy	2026	III	R/R DLBCL in association	230	median OS was 13.8 months vs. 8.5 (*p* = 0.009)	ORR 64% vs. 42% (*p* < 0.001) CR 40% vs. 19%	Median PFS: 4.2 months vs. 2.6 (*p* < 0.001)	NCT04404283	[148]

^(1)^ AVD = doxorubicin, vinblastine and dacarbazine; ABVD = doxorubicin, bleomycin, vinblastine and dacarbazine.

### 5.4. Anti-CD22 ADCs

Inotuzumab ozogamicin is an ADC composed of the anti-CD22 mAb inotuzumab linked to the cytotoxic agent calicheamicin. It was approved by the FDA in 2017 and by the EMA as a monotherapy for the treatment of adults with R/R CD22-positive B-cell precursor ALL [156]. In the phase III INO-VATE ALL trial (NCT01564784), inotuzumab ozogamicin demonstrated superior efficacy compared with standard of care in the overall study population and across prespecified subgroups, including high-risk disease (*p* = 0.0076) [157]. It was subsequently approved for pediatric patients with ALL (NCT02981628) [158]. Ongoing and recent clinical studies have evaluated its use in the first-line setting, either as monotherapy (NCT03460522), in combination with low-intensity chemotherapy (NCT03249870), or in combination with blinatumomab (NCT03739814) (Table 18) [159,160]. Inotuzumab ozogamicin is included in current treatment guidelines for frail older adults with acute lymphoblastic leukemia (ALL) who have multiple comorbidities and for whom conventional chemotherapy is not considered suitable because of its associated toxicity [161].

Moxetumomab pasudotox is an antibody–toxin fusion protein composed of the anti-CD22 mAb moxetumomab fused to a 38 kDa fragment of *Pseudomonas aeruginosa* exotoxin A. It was approved in 2018 (Lumoxiti^®^) for the treatment of R/R hairy cell leukemia after at least two prior lines of therapy [162]. However, its marketing authorization was withdrawn in Europe in 2021, followed by global discontinuation of commercialization in 2023 for commercial reasons [163,164].

**Table 18:** Summary of recent clinical trial for Inotuzumab in ALL.

Agent	Target	Year (Estimated Completion Date)	Phase	Indication	Cases	OS Rate (%)	CR/CRi (%)	Median Survival (Months)	Clinical Trial	Ref.
Inotuzumab ozogamicin vs. standard-of-care chemotherapy	CD22	2017	III	R/R ALL	326	NA	85.7% vs. 0%; *p* = 0.0076	NA	NCT01564784	[157]
Inotuzumab ozogamicin		2026	II	R/R ALL	48	NA	58.3%	NA	NCT02981628	[165]
Inotuzumab ozogamicin		2025	II	ALL	43	One-year OS 91% Three-year OS 73%	100%	NA	NCT03460522	[159]
Inotuzumab ozogamicin		2024	II	ALL	131	One-year OS 73.2%	NA	NA	NCT03249870	[166]
Inotuzumab ozogamicin induction then blinatumomab consolidation		2027	II	ALL	33	NA	97%	NA	NCT03739814	[167]

### 5.5. Anti-CD79 ADCs

Polatuzumab vedotin is an ADC targeting CD79b. It consists of the anti-CD79b mAb polatuzumab covalently linked to the antimitotic agent MMA E via a protease-cleavable linker. It was approved by the FDA in 2019, in combination with bendamustine and rituximab, for the treatment of adults with R/R DLBCL after at least two prior therapies [168]. In 2023, it was approved in the first-line setting for adult patients with previously untreated DLBCL, in combination with rituximab, cyclophosphamide, doxorubicin, and prednisone [169].

The phase III POLARIX trial (NCT03274492) demonstrated the benefit of adding polatuzumab to rituximab-based chemotherapy in the first-line treatment of DLBCL, showing improved 5-year PFS with significant results vs. Rituximab + CHOP chemotherapy (Table 19) [170]. Its combination with bsAbs such as mosunetuzumab has also shown promising outcomes in R/R DLBCL compared with the standard rituximab-based R-GemOx regimen, with a significant improvement in ORR and PFS—supporting the development of chemotherapy-sparing treatment strategies [171]. Polatuzumab is incorporated into the NCCN guidelines for the management of DLBCL as a second-line therapy (relapsed disease <12 months or primary refractory disease) [59].

### 5.6. Anti-CD19 ADCs

Loncastuximab tesirine is an ADC targeting CD19, consisting of the anti-CD19 mAb loncastuximab covalently linked to a pyrrolobenzodiazepine DNA-alkylating payload via a cleavable linker. It was approved by the FDA in 2021 for the treatment of R/R DLBCL [172], following the results of the phase II LOTIS-2 study (NCT03589469) (Table 20) [173]. Updated analyses of the LOTIS-2 study single arm in R/R DLBCL have confirmed durable efficacy and survival benefit, with an ORR of 48.3% and a median OS of 9.5 months. Among patients achieving a CR, median OS and PFS were not reached [174]. It is currently being investigated in additional indications, including R/R NHL (NCT02669017), FL (NCT04998669), and ALL (NCT02669264) [175].

A potential synergistic effect has been observed when loncastuximab tesirine is combined with rituximab in patients with R/R FL (study NCT04998669), with an acceptable safety profile, including 10% serious treatment-emergent adverse events [176]. To date, no clinical trial with a comparator arm has demonstrated the superiority of loncastuximab tesirine as a monotherapy in relapsed or refractory DLBCL. Nevertheless, it remains included in the NCCN Guidelines in combination with rituximab for the treatment of R/R FL and for DBLCL after ≥2 lines of systemic therapy [59,177].

### 5.7. Anti-BCMA ADCs

Belantamab mafodotin is an ADC composed of the anti-BCMA mAb belantamab linked to the microtubule inhibitor MMA F. Data from the phase II DREAMM-2 study supported its initial FDA approval in 2020 for R/R MM (Table 21) [178,179]. However, the lack of a statistically significant PFS benefit compared with pomalidomide plus dexamethasone in the phase III DREAMM-3 study led to the withdrawal of its authorization in the United States in 2022 [180,181]. The phase III DREAMM-8 study (NCT04484623) subsequently demonstrated a significantly improved 12-month PFS with belantamab mafodotin, pomalidomide, and dexamethasone (BPd) compared with pomalidomide, bortezomib, and dexamethasone (PVd) in lenalidomide-exposed patients with R/R MM after at least one prior line of therapy. Belantamab mafodotin was later approved in China in 2026 [182,183].

HDP-101 (pamlectabart tismanitin) is an ADC targeting BCMA and conjugated to the α-amanitin toxin, which inhibits RNA polymerase II. It is currently under clinical development for R/R MM. Preclinical studies demonstrated superior antitumor activity compared with belantamab mafodotin in vitro and in a del(17p) xenograft model [184]. These findings supported its progression into clinical development, with two ongoing studies: a phase I trial (NCT07529782) and a phase I/II trial (NCT04879043) [185,186]. Based on preclinical evidence and early clinical development data, the FDA granted Fast Track designation in 2025 for the treatment of R/R MM [187].

### 5.8. Anti-CD138 ADCs

Indatuximab ravtansine is an ADC composed of the anti-CD138 mAb indatuximab linked to a tubulin-binding maytansinoid payload. It has demonstrated synergistic activity in combination with lenalidomide in preclinical MM models and in a phase I/II clinical trial without a comparator or standard-of-care group (Table 22) [188,189,190]. However, its clinical development has not advanced significantly since completion of the phase I/II study in 2018.

### 5.9. Anti-CD25 ADCs

Camidanlumab tesirine is an ADC associating the anti-CD25 mAb camidanlumab with the DNA-alkylating payload tesirine via a cleavable linker. Its clinical development is most advanced in HL, although it has not yet received FDA approval. Early results from a phase I study (NCT02432235) demonstrated a manageable safety profile, with the maximum tolerated dose not being reached (Table 23) [191]. A subsequent phase II study (NCT04052997) in patients with R/R HL after brentuximab vedotin showed encouraging CRR, although treatment discontinuation due to adverse events occurred in 28.2% of patients, mainly due to skin toxicities and immune-mediated neurologic events (Table 23) [192]. Overall, camidanlumab tesirine has shown promising activity in R/R HL, particularly after prior brentuximab vedotin exposure; however, its clinical development has been limited by toxicity concerns.

### 5.10. Anti-CD123-ADCs

Pivekimab sunirine (IMGN632) is an ADC composed of an mAb targeting CD123 linked to a novel indolinobenzodiazepine payload. It has been investigated in blastic plasmacytoid dendritic cell neoplasm (BPDCN), where early clinical results from study NCT03386513 demonstrated high complete response rates and encouraging survival outcomes in patients with de novo disease (Table 24) [193,194]. Although not yet approved by regulatory authorities, pivekimab sunirine is currently under investigation in AML. A phase I study exploring its use in combination with fludarabine, cytarabin and idarubicin has helped define dosing strategies for further clinical development (NCT06034470). A phase I study is also ongoing in pediatric patients with R/R AML. A Phase II/III trial comparing pivekimab plus venetoclast plus azacytidine to the standard of care is currently underway to confirm its clinical efficacy (NCT07581002).

**Table 23:** Summary of recent clinical trial for Camidanlumab in R/R HL, NHL and MM.

Agent	Target	Year (Estimated Completion Date)	Phase	Indication	Cases	OS Rate (%)	CR/CRi (%)	Median Survival (Months)	Clinical Trial	Ref.
Camidanlumab tesirine	CD25	2023	II	R/R HL	117	median OS not reached	ORR 70.1%	Median PFS: 9.13 months	NCT04052997	[192]
Camidanlumab tesirine	CD25	2019	I	R/R HL and NHL	133	NA	ORR 71%	NA	NCT02432235	[194]

**Table 24:** Summary of recent clinical trial for Pivekimab in AML and BPDCN.

Agent	Target	Year (Estimated Completion Date)	Phase	Indication	Cases	OS Rate (%)	CR/CRi (%)	Median Survival (Months)	Clinical Trial	Ref.
Pivekimab sunirine (IMGN632)	CD123	2026	I/II	R/R AML	91	NA	ORR 21%	NA	NCT03386513	[195]
				R/R BPDCN	84	OS 16.6 months	CCR 75%	NA	NCT03386513	[194]

### 5.11. ADCs Targeting FMS-like Tyrosine Kinase 3

ASP1235 is an ADC combining a human mAb targeting FLT3 and a proprietary microtubule-disrupting agent (AGD-0182) via an oxime linker. It was evaluated as a monotherapy in patients with R/R AML in a phase I dose-escalation and expansion study (NCT02864290). However, its clinical development was discontinued during early clinical testing. Grade ≥ 3 adverse events occurred in 93% of patients, while efficacy data were considered insufficient to draw definitive conclusions (Table 25) [196].

### 5.12. ADC Overview

In AML management, the CD33-targeting ADC gemtuzumab ozogamicin has become a major therapeutic option. Although its initial use was limited by toxicity and the lack of superiority over standard of care in the first phase III clinical trial [130], the development of a new fractionated dosing regimen and the significant improvement in event-free survival (EFS) demonstrated in a second phase III study compared with standard chemotherapy enabled its broader adoption, including in newly diagnosed patients and in pediatric settings [133]. Another CD123-targeting ADC, pivekimab sunirine, achieved an ORR of 21% in a phase I/II study involving a limited cohort of 91 patients [195], supporting its continued development in a phase II/III trial vs. the standard-of-care combination of venetoclax and azacitidine to determine the optimal dose and assess clinical superiority (NCT06034470).

In ALL, only anti-CD22 ADCs have been developed, and the only one currently used in clinical practice is inotuzumab ozogamicin, which demonstrated significantly superior efficacy compared with standard of care in a phase III clinical trial involving a large cohort of 326 patients [157].

In MM, no ADC has obtained regulatory approval, with the exception of the BCMA-targeting pamlectabart tismanitin, which has held Fast Track Designation status since 2025.

In NHL, no ADC is currently marketed, as ibritumomab tiuxetan was withdrawn from the European market in 2024. In contrast, in HL, the CD30-targeting ADC brentuximab vedotin has been widely incorporated into clinical practice, including in pediatric patients, since its approval in 2011. This adoption has been supported by numerous clinical trials, including a phase III study vs. standard of care that demonstrated a significant PFS benefit in a large cohort of 260 patients [155]. In DLBCL, two ADCs have been approved by regulatory authorities: the CD19-targeting loncastuximab tesirine and the CD79-targeting polatuzumab vedotin. Of the two, only polatuzumab has demonstrated a significant improvement in ORR and MS when combined with mosunetuzumab vs. R-GemOx [171]; however, both ADCs are included in NCCN guidelines for the management of DLBCL as second-line therapy.

Despite their clear clinical benefit, the use of monoclonal antibodies remains limited by treatment-related adverse effects, including cytokine release syndrome (CRS), immune effector cell-associated neurotoxicity syndrome (ICANS), on-target off-tumor toxicity, and resistance mechanisms such as antigen escape (antigen loss and epitope masking), thereby driving the development of innovative immunotherapies [197].

## 6. Adoptive Cell Therapy: CAR T Cells

### 6.1. Definition

CAR T-cell therapy has revolutionized the management of hematological malignancies, particularly lymphomas and leukemias, following the development of anti-CD19 CAR T cells and their clinical implementation in the early 2010s. The principle of CAR T-cell therapy is to engineer T cells with a synthetic receptor that combines the antigen specificity of an antibody-derived binding domain with intracellular T-cell receptor signaling domains, thereby enabling antigen-specific cytotoxic activity [198].

Despite clinical success of CAR T-cell therapy, several challenges have emerged. Biological limitations include antigen escape, on-target/off-tumor toxicity, intrinsic CAR T-cell dysfunction, and an immunosuppressive tumor microenvironment. Manufacturing-related challenges include high cost, limited accessibility, production delays, and variability in patient-specific products. To address these limitations, next-generation, allogeneic and in vivo CAR T-cell approaches have been developed [1,199]. Next-generation CAR constructs incorporate additional signaling domains, including CD3-ζ for T-cell activation and costimulatory domains such as CD28 or 4-1BB, and may include additional modules designed to enhance proliferation, persistence, and cytokine signaling. Allogeneic CAR T cells are designed to provide standardized “off-the-shelf” products with improved availability and reduced manufacturing time compared with autologous approaches. However, they introduce additional risks, including immune rejection and graft-versus-host (GVH) disease. CAR T-cell research remains a rapidly evolving field aimed at improving efficacy, safety, manufacturing processes, and expanding clinical indications.

### 6.2. Approved CAR T Cells for Hematologic Malignancies

As of 2026, seven CAR T-cell therapies have been approved by the FDA and EMA, two target BCMA and are indicated for R/R MM, and five target CD19 and are indicated for R/R B-cell ALL or lymphomas (Table 26) [200,201].

Currently approved CAR T-cell therapies are primarily indicated for patients with R/R disease after failure of prior treatment lines. For example, the ELIANA trial demonstrated the remarkable efficacy of tisagenlecleucel, with an overall remission rate of 82% and a 12-month relapse-free survival rate of 59% in pediatric and young adult patients with R/R B-ALL [202]. Earlier use of CAR T-cell therapy is now being explored. The ongoing phase II CASSIOPEIA study is evaluating the efficacy and safety of tisagenlecleucel as a first-line treatment in high-risk pediatric and young adult patients with B-ALL who remain MRD-positive at the end of consolidation therapy. The trial is currently recruiting and plans to enroll 122 participants [203].

The major limitations of anti-CD19 CAR T-cell therapy continue to be relapse and treatment-related toxicity. Current research focuses on optimizing manufacturing processes, including CD34 selection to generate purer CAR T-cell products and reduce toxicity, as well as developing metabolically optimized CAR T cells with a Th1/Th17 hybrid phenotype that combines enhanced effector function with improved persistence, thereby increasing antitumor activity [204].

### 6.3. Development of CAR T-Cell Therapies in AML

To date, no CAR T-cell therapy has been approved by the FDA for the treatment of AML [205]. The development of CAR T cells for AML is particularly challenging because of the difficulty in identifying AML-specific target antigens; the aggressive nature of the disease; the detrimental effects of prior therapies on T-cell fitness; manufacturing constraints; and the highly immunosuppressive AML microenvironment, which limits CAR T-cell expansion and persistence [206,207].

The ideal CAR T-cell target is a surface antigen that is highly expressed on malignant cells while being absent from normal tissues, thereby minimizing on-target toxicity.

A phase II study evaluating CD19-directed CAR T cells in AML is currently ongoing [208]. Preliminary results showed complete remission in 10 treated patients, with 60% achieving MRD-negative complete remission. Median OS and leukemia-free survival were 11.6 and 3.8 months, respectively, with an acceptable safety profile (grade 1–2 CRS: 8/10 patients; grade 3 CRS: 1/10 patients) [208].

However, the principal antigens currently under investigation in AML are CD33, the interleukin-3 receptor α-chain (CD123) [209], and C-type lectin-like molecule-1 (CLL-1) [210]. However, both CD33 and CD123 are also expressed on normal hematopoietic cells, creating a risk of myelotoxicity, although extensive clinical experience with CD33-targeting ADCs such as gemtuzumab ozogamicin suggests that acceptable safety can be achieved through appropriate dose optimization [207,211]. Table 26 summarizes the main published clinical studies targeting these antigens. Among them, CLL-1 appears particularly attractive because it is expressed in 32.4% to 99.8% of newly diagnosed AML cases while being largely absent from normal hematopoietic tissues and other healthy organs [212]. Preliminary clinical data evaluating anti-CLL-1 CAR T cells are encouraging, especially considering that most enrolled patients had received multiple prior lines of therapy (Table 27).

PD-1 signaling is also recognized as an important mechanism limiting CAR T-cell efficacy by suppressing T-cell activation and proliferation. Accordingly, combining CAR T-cell therapy with PD-1 blockade has demonstrated enhanced antileukemic activity (including in patients with previous anti-CD38 CAR T-cell therapy) and reduced toxicity in preclinical models, findings that are beginning to be confirmed in early-phase clinical studies (Table 28) [212].

Finally, dual-targeting CAR T-strategies are being developed to reduce the risk of relapse associated with antigen escape. One clinical trial evaluated a CD33/CLL-1 dual-targeting CAR T construct for this purpose (NCT03795779) (Table 29) [225,226].

**Figure 4:**
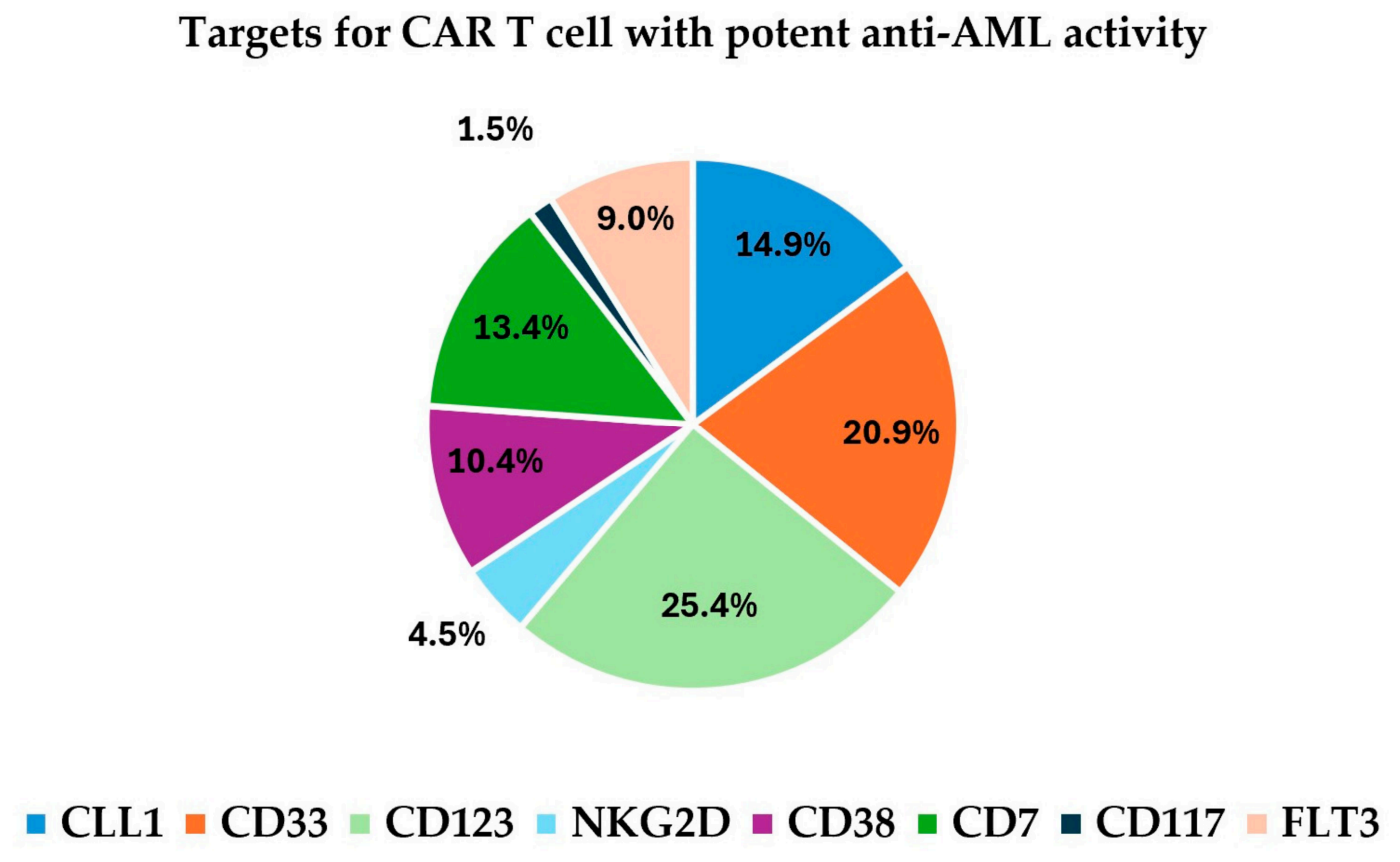
Targets for CAR T cells with potent anti-AML activity.

We expanded our review to include CAR T-cell strategies targeting additional antigens under investigation for the treatment of AML, including CD7, CD38, CD117 (c-KIT), FLT3, and NKG2D [205,228]. Ongoing clinical trials without published results were identified through a search of the ClinicalTrials.gov database (accessed 9 January 2026) using combinations of the keywords “CAR-T”, “AML”, and each respective target (NKG2D, CD38, CD7, CD117, and FLT3). The complete list of identified studies is provided in Supplementary Data, which summarizes the CAR T-cell targets currently under investigation for R/R AML. Figure 4 illustrates the relative distribution of these targets among ongoing clinical studies. CD123 remains the most extensively investigated target in AML [229], followed by CD33, CLL-1, CD7, CD38, FLT3, NKG2D, and CD117 [230].

CD7 is expressed on approximately 30% of myeloblasts in patients with AML. In a phase I clinical trial, 10 patients with AML received CD7-directed CAR T cells, of whom seven achieved complete remission with an acceptable safety profile; CRS was predominantly moderate in severity (80% of patients) [231].

CD38 is expressed on the majority of AML cells; however, its expression on immature hematopoietic cells, activated T cells, B cells, dendritic cells, and natural killer cells raises concerns regarding hematopoietic toxicity [232]. A case report involving two patients treated with CD38-directed CAR T cells described complete remission in both patients, with only grade 2 CRS being observed [233].

FLT3-directed UniCAR T-cell therapy has demonstrated encouraging antileukemic activity in both in vitro and in vivo models, although confirmation from ongoing clinical trials is still awaited [234].

The natural killer group 2D receptor (NKG2D) is an activating receptor that stimulates antitumor immunity through recognition of NKG2D ligands expressed on AML cells [235]. Targeting the NKG2D/NKG2D-L axis therefore represents an attractive therapeutic strategy. A phase I clinical trial evaluated an autologous CAR T-cell product based on NKG2D, which recognizes eight ligands overexpressed across several hematologic malignancies, including myeloid leukemias, MDS, and MM, while showing minimal expression on normal tissues. Preliminary results, with a median follow-up of 118 days, showed an objective response rate of 25% in patients with R/R AML or MDS, together with an acceptable safety profile (grade 3–4 treatment-related adverse events: 44%; grade 3–4 CRS: 31%, including one dose-limiting toxicity) [236].

Additional targets are also being explored for the development of novel CAR T-cell therapies for AML, including CD84, CD96, and Siglec-15. Preclinical studies of CD84-directed CAR T cells have demonstrated potent cytotoxic activity against AML cells while largely sparing HSPCs [237,238]. A phase I/II clinical trial evaluating CD84-directed CAR T-cell therapy in patients with R/R AML and T-cell ALL is currently ongoing [239].

CD96 is expressed on leukemic stem cells and blasts in approximately 48.3% of AML cases but is largely absent from normal HSPCs. In preclinical AML models, CD96-directed CAR T cells prolonged survival and effectively eliminated leukemic cells. Furthermore, combining CD96 CAR T cells with a CD33-targeted chimeric costimulatory receptor improved antitumor activity in CD96-low AML models, suggesting that this strategy may offer effective disease control while preserving normal hematopoiesis [240].

Siglec-15, a sialic acid-binding immunoglobulin-like lectin, is highly expressed on primary AML blasts and AML cell lines while showing minimal expression on HSPCs. Preclinical studies have demonstrated that anti-Siglec-15 CAR T cells effectively eliminate leukemic cells while preserving normal hematopoiesis [241].

Additionally, CAR T cells targeting IL-1RAP on the surface of leukemic stem cells have been developed for AML, and their pre-clinical production processes have been validated [242]. This has laid a solid foundation for their entry into phase I clinical trials, holding promise to bring new therapeutic hope to AML patients [243].

Finally, CAR natural killer (CAR NK) cells represent an attractive alternative to CAR T cells because of their potentially lower toxicity and improved selectivity. Their clinical application, however, remains limited by insufficient in vivo expansion and long-term persistence [244,245]. Early results from a phase I clinical trial evaluating CD33-directed CAR NK cells have shown encouraging efficacy and tolerability, although longer follow-up is required to confirm these findings (Table 30) [246].

Conventional CAR T cells rely on autologous cells manufactured ex vivo, which entails complex logistics, lengthy production times and high costs. Progress in in vivo gene delivery, highlighted by COVID-19 mRNA–LNP vaccines, has accelerated the exploration of in vivo CAR T-cell approaches [247]. In vivo CAR T-cell approaches are based on the direct delivery of vectors encoding chimeric antigen receptors into patients, enabling T-cell reprogramming in situ. Two major systems are used: viral vectors (e.g., AAV and lentivirus), and lipid nanoparticles (LNPs). A recent preclinical study in AML has demonstrated that in vivo CAR T-cell generation lipid nanoparticle-based mRNA delivery can induce transient CAR expression, leading to effective antileukemic activity. In situ engineering of CD8+ T cells (CD8 nanobody-targeted lipid nanoparticles encapsulating CD13 nanoCAR circRNAs) generated cytotoxic CAR T cells targeting AML [248]. To date, in vivo approaches are currently being evaluated in early-phase trials in B-cell malignancies and multiple myeloma, and are expected to expand to AML.

## 7. Conclusions and Perspectives

This review highlights the remarkable evolution of immunotherapy in oncohematology over the past three decades. Since the introduction of the first therapeutic mAbs—exemplified by rituximab, the first anti-CD20 antibody approved for the treatment of NHL—immunotherapeutic strategies have progressively transformed the management of hematologic malignancies. More recently, the focus of therapeutic innovation has shifted from conventional mAbs toward more sophisticated platforms, including bsAbs, ADCs, and adoptive cell therapies—particularly CAR T-cell therapies. Immunotherapy has helped reduce the risk of relapse, improve overall survival in some patients, and provide an alternative to intensive chemotherapy. The combination of immunotherapy and chemotherapy is now included in treatment guidelines for many hematologic malignancies to improve patient outcomes, including, for example, non-Hodgkin lymphoma (CHOP + rituximab), multiple myeloma (D-VRd + daratumumab), and acute myeloid leukemia (daunorubicin + cytarabine + gemtuzumab ozogamicin). According to the latest epidemiological data from the American Cancer Society, AML, NHL, and MM are expected to remain among the deadliest hematologic malignancies in 2026 [249]. In AML, approximately half of patients either fail induction therapy or experience relapse within six months [115], highlighting the urgent need for novel therapeutic approaches.

AML remains one of the hematologic malignancies for which immunotherapeutic development is least advanced. Current strategies include CD123/CD3 bsAbs such as flotetuzumab and vibecotamab, TsAbs targeting CD16/CD33/IL-15, and novel CD123-directed agents such as pivekimab [195]. In the field of adoptive cell therapies, no CAR T-cell therapy has yet received FDA approval for AML, although numerous clinical studies are investigating CAR T cells targeting CD123, CLL-1, and CD33. Nevertheless, most available evidence derives from early-phase clinical trials involving limited patient populations; additional studies are needed to establish their long-term efficacy and safety.

By contrast, immunotherapy has become an integral component of NHL management. Rituximab remains a cornerstone of first-line treatment in combination with chemotherapy, while newer generations of bsAbs, including blinatumomab and mosunetuzumab, are expanding the therapeutic arsenal. CAR T-cell therapies have also emerged as an established treatment option for several R/R B-cell lymphomas, illustrating the major progress achieved in this field.

MM has experienced the greatest diversification of immunotherapeutic strategies, with the approval of numerous mAbs, bsAbs, ADCs, and CAR T-cell therapies. BCMA has become the principal therapeutic target, although additional targets such as GPRC5D and FcRH5 are rapidly emerging. In particular, the FcRH5-directed bsAb cevostamab has demonstrated encouraging clinical activity and was highlighted as a promising therapeutic candidate during the 2024 ASH Annual Meeting [113].

Beyond currently approved therapies, several innovative approaches are under active investigation. Simultaneous multiple interaction T-cell engagers (SMITEs), which combine two complementary bsAbs, aim to enhance T-cell activation and overcome inhibitory mechanisms such as PD-L1-mediated immune suppression [250].

Similarly, NK-cell-based strategies, including TriKEs and CAR-engineered NK cells, seek to combine potent antitumor activity with improved safety profiles [128,245]. Although early clinical results are encouraging, limited in vivo persistence and insufficient cellular expansion remain important challenges for these emerging approaches.

Overall, the rapid diversification of immunotherapeutic modalities is reshaping the treatment landscape of hematologic malignancies. However, recent studies have shown that older individuals respond less effectively to immunotherapies because of age-related physiological alterations, including cellular senescence, chronic inflammation, and immunosenescence [251]. Immunosenescence particularly affects the qualitative functions of T lymphocytes, leading to reduced T-cell receptor diversity, impaired proliferative capacity, decreased cytotoxic activity, and altered antigen presentation—all within an unfavorable pro-inflammatory environment [252]. The CRR observed in younger patients with B-cell acute B-ALL (approximately 90%) is higher than that reported in older patients with multiple myeloma (33–73%). Senescence is one of the factors that may contribute to the reduced efficacy of CAR T-cell therapies in elderly patients [253]. As hematological malignancies predominantly affect older adults, the development of immunotherapeutic strategies targeting senescence has emerged as a major challenge. In this context, immune checkpoint inhibitors targeting Programmed Death-1 (PD-1) and Cytotoxic T-Lymphocyte-Associated Protein 4 (CTLA-4) appear to exert beneficial effects by restoring antitumor immunity and are currently being investigated for their potential to enhance immune responses in aging populations [254]. This rationale underlies the combination of immune checkpoint inhibitors with other immunotherapies, as illustrated by anti-CLL-1 CAR T-cell approaches in acute myeloid leukemia (AML), as well as their combination with antibody–drug conjugates (ADCs) such as brentuximab vedotin [255].

Overall, the rapid diversification of immunotherapeutic modalities is reshaping the treatment landscape of hematologic malignancies. Future research will likely focus on optimizing combination strategies, identifying novel targets, improving manufacturing processes, and extending these therapies to earlier lines of treatment, with the ultimate goal of achieving more durable responses while minimizing toxicity.

## Figures and Tables

**Table 1:** Estimated major hematologic malignancies’ new cases and deaths for 2026 [2].

Hematologic Malignancies	Leukemia				Lymphoma		Myeloma
	ALL	CLL	AML	CML	HL	NHL	MM
New cases in 2026	6250	22,760	22,720	9650	8920	79,320	36,000
Deaths in 2026	1600	4350	11,500	1170	1100	19,970	10,850

These stat facts focus on population statistics that are based on the U.S. population. Estimates of new cases and deaths for 2026 are projections made by the American Cancer Society (ACS), based on earlier reported data.

**Table 3:** Summary of recent clinical trial for CD20 × CD3 bsAbs Mosunetuzumab in R/R FL and R/R B-NHL.

Agent	Target	Year (Estimated Completion Date)	Phase	Indication	Cases	OS Rate	CR/CRi (%)	Median Survival (Months)	Clinical Trial	Ref.
Mosunetuzumab	CD20 × CD3	2025	I/II	R/R FL	90	82.4%	60%	35.9	NCT02500407	[74]
Mosunetuzumab	CD20 × CD3	2025	I/II	R/R NHL	229	NA	65.7% for indolent NHL 36.4% for agressive NHL	DoR 23.2 months	NCT02500407	[76]

**Table 5:** Summary of recent clinical trial for CD20/CD3 bsAbs epcoritamab in R/R FL.

Agent	Target	Year (Estimated Completion Date)	Phase	Indication	Cases	OS Rate	CR/CRi (%)	Median Survival (Months)	Clinical Trial	Ref.
Epcoritamab + lenalidomide (lena) + rituximab (ritux) vs. lena + ritux	CD20 × CD3	2029	III	R/R FL	488	NA	ORR: 95% vs. 79% *p* < 0.0001	NA	NCT05409066	[84]

**Table 6:** Summary of recent clinical trial for CD20/CD3 bsAbs Odronextamab in R/R FL and R/R DLBCL.

Agent	Target	Year (Estimated Completion Date)	Phase	Indication	Cases	OS Rate	CR/CRi (%)	Median Survival (Months)	Clinical Trial	Ref.
Odronextamab	CD20 × CD3	2028	II	R/R FL	128	NA	73.4%	20.7 months	NCT03888105	[86]
Odronextamab + CHOP vs. Rituximab + CHOP	CD20 × CD3	2029	III	Untreated CD20+ DLBCL	22	NA	100%	NA	NCT06091865	[87]
Odronextamab	CD20 × CD3	2025	I	R/R DLBCL after CAR T-cell therapy	60	NA	31.7%	NA	NCT02290951	[88]

**Table 7:** Summary of recent clinical trial for BCMA × CD3 bsAbs teclistamab in MM.

Agent	Target	Year (Estimated Completion Date)	Phase	Indication	Cases	OS Rate	CR/CRi (%)	Median Survival (Months)	Clinical Trial	Ref.
Teclistamab + daratumumab vs. daratumumab + dxm	BCMA × CD3	2028	III	R/R MM	587	NA	81.8% vs. 32.1%	36-month PFS: 83.4% vs. 29.7% *p* < 0.001	NCT05083169	[34]
Teclistamab + lenalidomide	BCMA × CD3	2032	III	Newly Diagnosed MM	94	NA	Among MRD + pts at study entry 100% converted to MRD -	NA	NCT05243797	[94]

**Table 8:** Summary of recent clinical trial for BCMA × CD3 bsAbs linvoseltamab in MM.

Agent	Target	Year (Estimated Completion Date)	Phase	Indication	Cases	OS Rate	CR/CRi (%)	Median Survival (Months)	Clinical Trial	Ref.
Linvoseltamab	BCMA × CD3	2033	I/II	R/R MM	117	NA	52%	Not reached after median follow-up of 29.4 months	NCT03761108	[98]
Linvoseltamab	BCMA × CD3	2033	III	R/R MM	NA	NA	NA	NA	NCT05730036	[99]

**Table 9:** Summary of recent clinical trial for BCMA × CD3 bsAbs elranatamab in MM.

Agent	Target	Year (Estimated Completion Date)	Phase	Indication	Cases	OS Rate	CR/CRi (%)	Median Survival (Months)	Clinical Trial	Ref.
Elranatamab	BCMA × CD3	2026	II	R/R MM	123	56.7% at 15 months	35%	NA	NCT04649359	[101]

**Table 10:** Summary of recent clinical trial for BCMA × CD3 bsAbs etentamig in MM.

Agent	Target	Year (Estimated Completion Date)	Phase	Indication	Cases	OS Rate	CR/CRi (%)	Median Survival (Months)	Clinical Trial	Ref.
ABBV-383 (etentamig)	BCMA × CD3	2026	I/II	R/R MM	124	ORR: 68% at doses ≥40 mg	NA	NA	NCT03933735	[106]

**Table 11:** Summary of recent clinical trial for GPRC5D × CD3 bsAbs talquetamab in MM.

Agent	Target	Year (Estimated Completion Date)	Phase	Indication	Cases	OS Rate	CR/CRi (%)	Median Survival (Months)	Clinical Trial	Ref.
Talquetamab	GPRC5D × CD3	2029	II	R/R MM	375	ORR: 74%	NA	NA	NCT04634552	[110]
Talquetamab + daratumumab	GPRC5D × CD3 + CD38 mAbs	2027	I	R/R MM	65	ORR: 82.4% (Q2W cohort)	NA	NA	NCT04108195	[35]
Talquetamab + teclistamab	GPRC5D × CD3 + BCMA × CD3	2026	I/II	R/R MM	94	ORR: 78%	NA	NA	NCT04586426	[111]

**Table 12:** Summary of recent clinical trial for FcRH5 × CD3 bsAbs Cevostamab in MM.

Agent	Target	Year (Estimated Completion Date)	Phase	Indication	Cases	OS Rate	CR/CRi (%)	Median Survival (Months)	Clinical Trial	Ref.
Cevostamab	FcRH5 × CD3	2027	I/II	R/R MM	21	ORR: 10%	NA	NA	NCT05535244	[114]
Cevostamab	FcRH5 × CD3	2026	I	R/R MM	167	ORR: 43.1%	13.2% (CR), 12.6% (VGPR) ^(1)^, and 17.4% (PR)	NA	NCT03275103	[112]

^(1)^ very good partial response (VGPR).

**Table 13:** Summary of recent clinical trial for CD123 × CD3 bsAbs in AML.

Agent	Target	Year (Estimated Completion Date)	Phase	Indication	Cases	OS Rate	CR/CRi (%)	Median Survival (Months)	Clinical Trial	Ref.
Flotetuzumab	CD123 × CD3	2022	I/II	AML	88	ORR: 30%	CR: 26.7%	Median OS: 10.2 months	NCT02152956	[115]
Vibecotamab	CD123 × CD3	2021	1	AML	120	ORR: 9%	NA	NA	NCT02730312	[116]

**Table 14:** Summary of recent clinical trial for tsAbs in MM and NHL.

Agent	Target	Year (Estimated Completion Date)	Phase	Indication	Cases	OS Rate (%)	CR/CRi (%)	Median Survival (Months)	Clinical Trial	Ref.
SAR442257	CD38, CD3 and CD28	2026	I	R/R MM R/R NHL	47	NA	ORR (MM): 5% ORR (NHL): 14.3%	NA	NCT04401020	[104]
Ramantamig JNJ-79635322	BCMA, GPRC5D, and CD3	2028	I	R/R MM	NA	NA	NA	NA	NCT05652335 NCT06768489	[123]
IBI3003	BCMA, GPRC5D, and CD3	2027	I/II	R/R MM	39	NA	ORR: 83.3% (median follow-up of 3.25 months)	NA	NCT06083207	[124]

**Table 16:** Summary of recent clinical trial for Gemtuzumab ozogamicin (GO) in AML.

Agent	Target	Year (Estimated Completion Date)	Phase	Indication	Cases	OS Rate (%)	CR/CRi (%)	Median Survival (Months)	Clinical Trial	Ref.
Gemtuzumab Ozogamicin (GO) + cytarabine + daunorubicin vs. daunorubicin + cytarabine	CD33 + chemotherapy	2013	III	2 years of age and older *de novo* AML	271	OS 27.5 months vs. 21.8 months (*p* = 0.16)	CR 70.4% vs. 69.9%	NA	NCT00927498	[130]
GO + anthracycline and cytarabine -based chemotherapy vs. conventional chemotherapy	CD33 + chemotherapy	2016	III	pediatric patients with *KMT2A*-r (AML)	1022	OS 63% vs. 53%, *p* = 0.054	NA	EFS: 48% with GO vs. 29% without, *p* = 0.003	NCT01407757	[133]

**Table 19:** Summary of recent clinical trial for Polatuzumab in LBCL and DLBCL.

Agent	Target	Year (Estimated Completion Date)	Phase	Indication	Cases	OS Rate (%)	CR/CRi (%)	Median Survival (Months)	Clinical Trial	Ref.
Polatuzumab vedotin + mosunetuzumab vs. R-GemOx ^(1)^	CD79b + bsAbs CD20/CD3	2027	III	R/R LBCL	208	NA	ORR 70% vs. 40% *p* < 0.0001	Median PFS: 11.5 months vs. 3.8 (*p* < 0.0001)	NCT05171647	[171]
Polatuzumab vedotin + Rituximab + CHP ^(2)^ chemotherapy vs. Rituximab + CHOP ^(3)^ chemotherapy	CD79b	2026	III	DLBCL	879	significant 5-year PFS benefit over R-CHOP (64.9% vs. 59.1% HR = 0.77 [95% CI, 0.62 to 0.97]	NA	NA	NCT03274492	[170]

^(1)^ R-GemOx = rituximab, gemcitabine, and oxaliplatin; ^(2)^ CHP = cyclophosphamide, doxorubicin, and prednisone; ^(3)^ CHOP = cyclophosphamide, doxorubicin, vincristine, and prednisone.

**Table 20:** Summary of recent clinical trial for Loncastuximab in FL and DLBCL.

Agent	Target	Year (Estimated Completion Date)	Phase	Indication	Cases	OS Rate (%)	CR/CRi (%)	Median Survival (Months)	Clinical Trial	Ref.
Loncastuximab tesirine + Rituximab	CD19 + mAbs CD20	2030	II	R/R FL	39	NA	67%	NA	NCT04998669	[176]
Loncastuximab tesirine	CD19	2022	II	R/R DLBCL	184	NA	ORR 48.3% (CR + PR)	NA	NCT03589469	[173]

**Table 21:** Summary of recent clinical trial for Belantamab in R/R MM.

Agent	Target	Year (Estimated Completion Date)	Phase	Indication	Cases	OS Rate (%)	CR/CRi (%)	Median Survival (Months)	Clinical Trial	Ref.
Belantamab mafodotin	BCMA	2024	II	R/R MM	293	NA	ORR 65% across all cohort	NA	NCT03525678	[178]
Belantamab mafodotin vs. pomalidomide + dxm	BCMA	2027	III	R/R MM	325	NA	NA	Median PFS: 11.2 months vs. 7.0; *p* = 0.56	NCT04162210	[181]
Belantamab mafodotin + pomalidomide + dexamethasone vs. Pvd	BCMA	2029	III	R/R MM	302	NA	40% (BPd) vs. 16% (PVd)	12-month estimated PFS: 71% (BPd) vs. 51% (PVd) *p* < 0.001)	NCT04484623	[183]

**Table 22:** Summary of recent clinical trial for Indatuximab in R/R MM.

Agent	Target	Year (Estimated Completion Date)	Phase	Indication	Cases	OS Rate (%)	CR/CRi (%)	Median Survival (Months)	Clinical Trial	Ref.
Indatuximab ravtansine + lenalidomide or Indatuximab ravtansine + pomalidomide	CD138	2018	I/II	R/R MM	64	NA	ORR 71·7% and 70.6%	NA	NCT01638936	[188]

**Table 25:** Summary of recent clinical trial for ASP1235 in AML.

Agent	Target	Year (Estimated Completion Date)	Phase	Indication	Cases	OS Rate (%)	CR/CRi (%)	Median Survival (Months)	Clinical Trial	Ref.
ASP1235	FLT3R	2020	I	R/R AML	43	NA	CR: 6/45	NA	NCT02864290	[196]

**Table 26:** Current status of available and approved CAR T-cell therapies.

Generic Name	Brand Name	Target	Indication FDA	First Approval Year
Tisagenlecleucel	Kymriah	CD19	B-ALL (pediatric/young adult) DLBCL FL	2017
Axicabtagene ciloleucel	Yescarta	CD19	DLBCL FL	2017
Idecabtagene vicleucel	Abecma	BCMA	MM	2021
Lisocabtagene maraleucel	Breyanzi	CD19	FL DLBCL Mantle cell lymphoma CLL	2021
Bexucabtagene autoleucel	Tecartus	CD19	B-cell ALL (adult) Mantle cell lymphoma	2021
Ciltacabtagene autoleucel	Carvykti	BCMA	MM	2022
Obecabtagene autoleucel	Aucatzyl	CD19	B-ALL (adult)	2024

**Table 27:** Summary of the clinical trial results of CD123, CLL-1, and CD33 CAR therapy.

Agent	Target	Estimated Completion Date	Phase	Indication	Enrollment	Results: Survival Rate (%); Complete Remission (CR/CRi) (%); Median PFS; OS (Month); ORR; MRD; CR; Event/Free Survival (EFS); Partial Remission (PR); Morphologic Leukemic-Free State (MLFS)	Side Effects	Clinical Trial Identifier (Reference)
CD123 CAR T								
CD123 CAR T	CD123	2026-08-06	1	R/R AML (cohort 1)	6	CR: 50% (3/6)	CRS * (4 grade 1; 1 grade 2) Adenoviral pneumonia: 1 Rash: 1	NCT02159495 [209]
CD123 CAR T	CD123	2016-11-18	Early 1	R/R AML	5	Disease progression before d28	CRS: 50% grade 3; 8% grade 4	NCT02623582 [213]
CD123 CAR T	CD123	2026-08-06	1	R/R AML	7	MLFS d28 (1/7) CR (2/7)	No grade 3 or above CRS	NCT02159495 [214]
CD123 CAR T	CD123	2033-12-03	1	R/R AML	12	CR at d28: 2/12 MRD-/CR+ with incomplete count recovery (CRi): 1/12 Stable or progressive disease: 3/12 MRD-: 3/12	Death related to CAR T toxicity (grade 5 CRS) (2/12) CRS (10/12)	NCT03766126 [215]
CD123 CAR T	CD123	2030-07-29	1	R/R AML	6	anti-AML activity as evidenced by responses (2/6)	High-grade fevers within 3 h of infusion (100%) CRS grade 2 (2/6) CRS grade 4/5 (3/6)	NCT04318678 [216]
CD123 CAR T	CD123	2025-04-11	1	R/R AML	12	CRi (2/12) MRD positive CR converted to level of negativity (1/12) PR (4/12)	CRS grade 1/2 (100%)	NCT04230265 [217]
CD123 CAR T	CD123	2025-12	1	R/R AML	16	MLFS (1/16) SD (2/16) MRD-negative CR (1/16)	CRS (15/16): grade 3 (1/16) grade 4 (2/16) ICANS grade 3 (1/16)	NCT03190278 [218]
CLL-1 CAR T								
CLL-1 CAR T	CLL-1	Registration date: 2020-12-17	1	R/R AML	38	ORR: 73.7% (28/38) MRD-CR: 42.1% PFS: 9 months (95%CI 3.03–14.47) OS: 12.2 months (95%CI 3.0–24.9)	CRS (17 grades 3/4) ICANS ** (1 grade 4)	ChiCTR2000041054 [219]
CLL-1 CAR T low dose	CLL-1	2024-05-18	1	R/R AML	3	OS: 2.6 months (1.1 to 7.5) EFS: 26 days (15 to 28)	Febrile neutropenia (3/3) Sepsis (3/3)	NCT04789408 [220]
CLL-1 CAR T higher dose					3	OS: 4.7 months (2.9 to 12.3) EFS: 23 days (9 to 42)	Cardiac disorders (3/3) Respiratory failure (1/3)	
CLL-1 CAR T highest dose					6	OS: 2.6 months (0.9 to 6.3) EFS: 21.5 days (14 to 48)	CRS grade 3/4 or ICANS grade 4: 20%	
CLL-1 CAR T	CLL-1			R/R AML in children	7	CR: 5/7 1-Year survival: 57.1% 6/7 patients died from relapse or progression disease after 23 months post-CAR T-cell infusion	CRS grade 1/2 (7/7) ICANS (1 patient) Impairment of liver function (3/7) WBC count decrease grade 3/4 (7/7) Anemia grade 3/4 (5/7) Thrombocytopenia grade 3/4 (6/7)	ChiCTR1900027684 [221]
CLL-1 CAR T	CLL-1	2020-12-31	1/2	R/R AML in children	4	MRD- and CR (3/4) 3/4 patients died after 24 months post-CAR-T infusion	CRS (3 grade 1/2) ICANS (1 grade 1/2) Neutropenia grade 4 (3/4) Anemia grade 3 (4/4)	NCT03222674 [222]
CD33 CAR-T								
CD33 CAR T	CD33	2019-10-10	1	R/R AML	3	Death from disease progression (3/3)	CRS grade 2/3 (2/10) ICANS grade 2 (1/10)	NCT03126864 [223]
CD33 CAR T bone marrow blasts ≥ 5% Arm A or <5% Arm B	CD33	2025-05-28	1/2	R/R AML	15	ORR: 20% CR (2/15) with incomplete count recovery in Arm A and 1 patient achieved MRD clearance in Arm B	CRS < grade 3 (93.3%) ICANS grade 3 (1/15) GVH *** grade 3 (1/15)	NCT05984199 [224]

* Cytokine release syndrome (CRS); ** Immune effector cell-associated neurotoxicity syndrome (ICANS); *** GVH: graft-versus-host disease.

**Table 28:** Summary of the clinical trial results of CLL-1 PD-1 KD CAR therapy.

Agent	Target	Estimated Completion Date	Phase	Indication	Enrollment	Results: Survival Rate (%); Complete Remission (CR/CRi) (%); Median PFS; OS (Month); ORR; MRD; CR; Event/Free Survival (EFS); Partial Remission (PR); Morphologic Leukemic-Free State (MLFS)	Side Effects	Clinical Trial Identifier (Reference)
PD-1 KD CLL-1 CAR T	CLL-1	2024-12-31	1/2	R/R AML	2	MRD-: 8 months (1 patient); 28 d (1 patient)	CRS (1 patient grade 1; 1 patient grade 2)	NCT04884984 [212]

**Table 29:** Summary of the clinical trial results of CD33/CLL1 CAR therapy.

Agent	Target	Estimated Completion Date	Phase	Indication	Enrollment	Results: Survival Rate (%); Complete Remission (CR/CRi) (%); Median PFS; OS (Month); ORR; MRD; CR; Event/Free Survival (EFS); Partial Remission (PR); Morphologic Leukemic-Free State (MLFS)	Side Effects	Clinical Trial Identifier (Reference)
CD33/ CLL1 CAR T	CD33/ CLL1	2022-09-30	Early phase I	R/R AML	9	MRD—(7/9)	CRS * (3 grade 1; 3 grade 2; 2 grade 3) CRES ** (1 grade 2; 2 grade 3; 1 grade 4)	NCT03795779 [227]

* Cytokine release syndrome (CRS); ** CAR T-cell-related encephalopathy (CRES).

**Table 30:** Summary of the clinical trial results of anti-CD33 CAR NK.

Agent	Target	Estimated Completion Date	Phase	Indication	Enrollment	Results: Survival Rate (%); Complete Remission (CR/CRi) (%); Median PFS; OS (Month); ORR; MRD; CR; Event/Free Survival (EFS); Partial Remission (PR); Morphologic leukemic-Free State (MLFS)	Side Effects	Clinical Trial Identifier (Reference)
Anti-CD33 CAR NK	CD33	2023-12-01	1	R/R AML	10	MRD-negative complete remission at d28: 6/10	CRS grade 2 (1/10)	NCT05008575 [246]

## Data Availability

No new data were created or analyzed in this study.

## Supplementary Materials

The following supporting information can be downloaded at: https://www.mdpi.com/article/10.3390/cells15161439/s1, Table S1: Table summarizes the targets investigated in research on CAR-T cell targeting AML.

## Abbreviations

The following abbreviations are used in this manuscript:

ADC	Antibody-drug conjugate
ADCC	Antibody-dependent cellular cytotoxicity
ALL	Acute lymphoblastic leukemia
AML	Acute myeloid leukemia
ASCT	Autologous Stem Cell Transplant
B-ALL	B-cell acute lymphoblastic leukemia
B-CLL	B-cell chronic lymphocytic leukemia
BCMA	B-cell maturation antigen
B-NHL	B-cell non-Hodgkin lymphoma
BPDCN	Blastic plasmacytoid dendritic cell neoplasm
bsAb	Bispecific antibody
CAR	Chimeric antigen receptor
CAR NK	Chimeric antigen receptor natural killer
CHP	Cyclophosphamide, doxorubicin, prednisone
CHOP	cyclophosphamide, doxorubicin, vincristine, and prednisone
CLL	Chronic lymphocytic leukemia
CLL-1	C-type lectin-like molecule-1
CML	Chronic myeloid leukemia
CRc	Composite complete remission
CRS	Cytokine release syndrome
DoR	Duration of response
DXM	Dexamethasone
DLBCL	Diffuse large B-cell lymphoma
EFS	Event-free survival
EMA	European Medicines Agency
FDA	U.S. Food and drug administration
FL	Follicular lymphoma
GO	Gemtuzumab ozogamicin
GVH	Graft-versus-host
HL	Hodgkin lymphoma
HSCT	Hematopoietic stem cell transplantation
ICANS	Immune effector cell-associated neurotoxicity syndrome
mAbs	Monoclonal antibody
MDS	Myelodysplastic syndrome
MM	Multiple myeloma
MMA	Monomethyl auristatin
MRD	Minimal residual disease
MS	Median survival
NHLs	Non-Hodgkin lymphomas
NK	Natural killer
NKG2D	Natural killer group 2D receptor
ORR	Overall response rate
OS	Overall survival
PFS	Progression-free survival
POD	Progression of disease
Pvd	Pomalidomide, bortezomib and dexamethasone
R-GemOx	Rituximab, gemcitabine, and oxaliplatin
R/R	Relapsed or refractory
SMITE	Simultaneous multiple interaction T-cell engager
TKI	Tyrosine kinase inhibitor
TriKE	Trispecific killer engager
TsAb	Trispecific antibody
VGPR	Very good partial response

## References

[1] Tang L., Huang Z., Mei H., Hu Y. (2023). Immunotherapy in Hematologic Malignancies: Achievements, Challenges and Future Prospects. Signal Transduct. Target. Ther..

[2] SEER Cancer Stat Facts. https://seer.cancer.gov/statfacts/index.html.

[3] Bachireddy P., Burkhardt U.E., Rajasagi M., Wu C.J. (2015). Hematologic Malignancies: At the Forefront of Immunotherapeutic Innovation. Nat. Rev. Cancer.

[4] Jureczek J., Kałwak K., Dzięgiel P. (2024). Antibody-Based Immunotherapies for the Treatment of Hematologic Malignancies. Cancers.

[5] Grillo-López A.J., White C.A., Dallaire B.K., Varns C.L., Shen C.D., Wei A., Leonard J.E., McClure A., Weaver R., Cairelli S. (2000). Rituximab: The First Monoclonal Antibody Approved for the Treatment of Lymphoma. Curr. Pharm. Biotechnol..

[6] Biopharma Dealmakers. https://www.nature.com/biopharmdeal.

[7] RITUXAN^®^ (Rituximab) Medication for RA, NHL, CLL, GPA, MPA & PV. https://www.rituxan.com.

[8] Chemo-Free Regimens Show Promise, Could Redefine Care for B-Cell Lymphomas—Dana-Farber Cancer Institute. https://www.dana-farber.org/newsroom/news-releases/2025/chemo-free-regimens-show-promise-could-redefine-care-for-b-cell-lymphomas.

[9] Liebman H.A., Saleh M.N., Bussel J.B., Negrea O.G., Horne H., Wegener W.A., Goldenberg D.M. (2016). Comparison of Two Dosing Schedules for Subcutaneous Injections of Low-Dose Anti-CD20 Veltuzumab in Relapsed Immune Thrombocytopenia. Haematologica.

[10] Ganjoo K.N., de Vos S., Pohlman B.L., Flinn I.W., Forero-Torres A., Enas N.H., Cronier D.M., Dang N.H., Foon K.A., Carpenter S.P. (2015). Phase 1/2 Study of Ocaratuzumab, an Fc-Engineered Humanized Anti-CD20 Monoclonal Antibody, in Low-Affinity FcγRIIIa Patients with Previously Treated Follicular Lymphoma. Leuk. Lymphoma.

[11] Jacobs R., Jurczak W., Flinn I.W., Grosicki S., Giannopoulos K., Wróbel T., Zafar S.F., Cultrera J.L., Kambhampati S., Danilov A.V. (2021). Efficacy and Safety of Ublituximab in Combination with Umbralisib (U2) in Patients with Chronic Lymphocytic Leukemia (CLL) By Treatment Status: A Sub-Analysis of the Phase 3 Unity-CLL Study. Blood.

[12] Lee H.-Z., Miller B.W., Kwitkowski V.E., Ricci S., DelValle P., Saber H., Grillo J., Bullock J., Florian J., Mehrotra N. (2014). U.S. Food and Drug Administration Approval: Obinutuzumab in Combination with Chlorambucil for the Treatment of Previously Untreated Chronic Lymphocytic Leukemia. Clin. Cancer Res..

[13] Lemery S.J., Zhang J., Rothmann M.D., Yang J., Earp J., Zhao H., McDougal A., Pilaro A., Chiang R., Gootenberg J.E. (2010). U.S. Food and Drug Administration Approval: Ofatumumab for the Treatment of Patients with Chronic Lymphocytic Leukemia Refractory to Fludarabine and Alemtuzumab. Clin. Cancer Res..

[14] Inman S. FDA Approves First-Line Ofatumumab for Fludarabine-Ineligible Patients with CLL—OncLive. https://www.onclive.com/view/fda-approves-first-line-ofatumumab-for-chemotherapy-ineligble-patients-with-cll.

[15] American Association for Cancer Research (2014). Obinutuzumab Breaks through to FDA Approval. Cancer Discov..

[16] Goede V., Fischer K., Busch R., Engelke A., Eichhorst B., Wendtner C.M., Chagorova T., de la Serna J., Dilhuydy M.-S., Illmer T. (2014). Obinutuzumab plus Chlorambucil in Patients with CLL and Coexisting Conditions. N. Engl. J. Med..

[17] Marcus R., Davies A., Ando K., Klapper W., Opat S., Owen C., Phillips E., Sangha R., Schlag R., Seymour J.F. (2017). Obinutuzumab for the First-Line Treatment of Follicular Lymphoma. N. Engl. J. Med..

[18] Robinson R., Berger T., Shochat T., Aumann S., Nachmias B., Goldschmidt N., Horesh N., Harel R., Aviv A., Shimony S. (2026). Obinutuzumab Maintenance in Follicular Lymphoma Is Associated with Better Progression Free Survival and Comparable Toxicity. Eur. J. Haematol..

[19] Abramson J.S., Ku M., Hertzberg M., Huang H.-Q., Fox C.P., Zhang H., Yoon D.H., Kim W.-S., Abdulhaq H., Townsend W. (2024). Glofitamab plus Gemcitabine and Oxaliplatin (GemOx) versus Rituximab-GemOx for Relapsed or Refractory Diffuse Large B-Cell Lymphoma (STARGLO): A Global Phase 3, Randomised, Open-Label Trial. Lancet.

[20] Ghosh N., Bellasea S., Li H., Olszewski A.J., Bryan L., Danilov A., Smith S., LeBlanc M., Friedberg J.W. (2025). SWOG 2308: Randomized Phase III Study of Mosunetuzumab Versus Rituximab for Low-Tumor Burden Follicular Lymphoma. JCO Oncol. Adv..

[21] Munoz J., Rosenthal A., Ip A., Darrah J.M., Wang T., Zhang G., Mutebi A., Branchcomb T., Ding Z., Chhibber A. (2026). Epcoritamab Plus Gemcitabine and Oxaliplatin Versus Rituximab Plus Gemcitabine and Oxaliplatin in Transplant-Ineligible Relapsed/Refractory Diffuse Large B-Cell Lymphoma: A Match-Adjusted Comparative Analysis. Clin. Lymphoma Myeloma Leuk..

[22] Hardin C., Gray C., Novelli S., Hacibekiroglu T., Yağcı M., Birhiray R.E., Risal A., Namuduri M., Cai J., Ufkin M. (2024). Phase 3 Trial Evaluating the Efficacy and Safety of Odronextamab plus Chemotherapy versus Rituximab plus Chemotherapy in Previously Untreated Follicular Lymphoma (OLYMPIA-2). J. Clin. Oncol..

[23] CBER—Alemtuzumab (Campath), Millennium and ILEX Partners, LP. https://www.accessdata.fda.gov/drugsatfda_docs/appletter/2001/alemmil050701L.htm.

[24] Demko S., Summers J., Keegan P., Pazdur R. (2008). FDA Drug Approval Summary: Alemtuzumab as Single-Agent Treatment for B-Cell Chronic Lymphocytic Leukemia. Oncologist.

[25] Serani S. Alemtuzumab Wins FDA Orphan Drug Designation as Part of ALL CAR T Therapy—Targeted Oncology—Immunotherapy, Biomarkers, and Cancer Pathways. https://www.targetedonc.com/view/alemtuzumab-wins-fda-orphan-drug-designation-as-part-of-all-car-t-therapy.

[26] Kadia T.M., Jain A., Rausch C.R., Bataller A., Ravandi F., Jabbour E., Qiao W., Borthakur G., Short N., Montalban-Bravo G. (2026). Phase 1B Pilot Study of Itacitinib with Alemtuzumab in Patients with T-Cell Prolymphocytic Leukemia. Blood Neoplasia.

[27] Staber P.B., Herling M., Bellido M., Jacobsen E.D., Davids M.S., Kadia T.M., Shustov A., Tournilhac O., Bachy E., Zaja F. (2019). Consensus Criteria for Diagnosis, Staging, and Treatment Response Assessment of T-Cell Prolymphocytic Leukemia. Blood.

[28] d’Amore F., Federico M., de Leval L., Ellin F., Hermine O., Kim W.S., Lemonnier F., Vermaat J.S.P., Wulf G., Buske C. (2025). Peripheral T- and Natural Killer-Cell Lymphomas: ESMO-EHA Clinical Practice Guideline for Diagnosis, Treatment and Follow-Up. Ann. Oncol..

[29] Liste des Spécialités en Accès Dérogatoire—Campath. https://ansm.sante.fr/tableau-acces-derogatoire/campath.

[30] Raedler L.A. (2016). Darzalex (Daratumumab): First Anti-CD38 Monoclonal Antibody Approved for Patients with Relapsed Multiple Myeloma. Am. Health Drug Benefits.

[31] Usmani S.Z., Facon T., Hungria V., Bahlis N.J., Venner C.P., Braunstein M., Pour L., Martí J.M., Basu S., Cohen Y.C. (2025). Daratumumab plus Bortezomib, Lenalidomide and Dexamethasone for Transplant-Ineligible or Transplant-Deferred Newly Diagnosed Multiple Myeloma: The Randomized Phase 3 CEPHEUS Trial. Nat. Med..

[32] Moreau P., Hulin C., Perrot A., Arnulf B., Belhadj K., Benboubker L., Zweegman S., Caillon H., Caillot D., Avet-Loiseau H. (2024). Bortezomib, Thalidomide, and Dexamethasone with or without Daratumumab and Followed by Daratumumab Maintenance or Observation in Transplant-Eligible Newly Diagnosed Multiple Myeloma: Long-Term Follow-up of the CASSIOPEIA Randomised Controlled Phase 3 Trial. Lancet Oncol..

[33] Dimopoulos M.A., Voorhees P.M., Schjesvold F., Cohen Y.C., Hungria V., Sandhu I., Lindsay J., Baker R.I., Suzuki K., Kosugi H. (2025). Daratumumab or Active Monitoring for High-Risk Smoldering Multiple Myeloma. N. Engl. J. Med..

[34] Costa L.J., Bahlis N.J., Perrot A., Nooka A.K., Lu J., Pawlyn C., Mina R., Caeiro G., Kentos A., Hungria V. (2026). Teclistamab plus Daratumumab in Relapsed or Refractory Multiple Myeloma. N. Engl. J. Med..

[35] Chari A., van de Donk N.W.C.J., Dholaria B., Weisel K., Mateos M.-V., Goldschmidt H., Martin T.G., Morillo D., Reece D., Rodríguez-Otero P. (2025). Talquetamab plus Daratumumab for the Treatment of Relapsed or Refractory Multiple Myeloma in the TRIMM-2 Study. Blood.

[36] Dhillon S. (2020). Correction to: Isatuximab: First Approval. Drugs.

[37] Center for Drug Evaluation and Research (2024). FDA Approves Isatuximab-Irfc with Bortezomib, Lenalidomide, and Dexamethasone for Newly Diagnosed Multiple Myeloma.

[38] Facon T., Dimopoulos M.-A., Leleu X.P., Beksac M., Pour L., Hájek R., Liu Z., Minarik J., Moreau P., Romejko-Jarosinska J. (2024). Isatuximab, Bortezomib, Lenalidomide, and Dexamethasone for Multiple Myeloma. N. Engl. J. Med..

[39] FDA Grants Orphan Drug Designation to SAR446523 for the Treatment of RRMM. https://multiplemyelomahub.com/medical-information/fda-grants-orphan-drug-designation-to-sar446523-for-the-treatment-of-rrmm.

[40] Bristol-Myers Squibb and AbbVie Receive FDA Approval of Empliciti^TM^ (Elotuzumab) for the Treatment of Patients with Multiple Myeloma Who Have Received One to Three Prior Therapies. https://news.bms.com/news/details/2015/Bristol-Myers-Squibb-and-AbbVie-Receive-FDA-Approval-of-Empliciti-elotuzumab-for-the-Treatment-of-Patients-with-Multiple-Myeloma-Who-Have-Received-One-to-Three-Prior-Therapies/default.aspx.

[41] Dimopoulos M.A., Dytfeld D., Grosicki S., Moreau P., Takezako N., Hori M., Leleu X., LeBlanc R., Suzuki K., Raab M.S. (2023). Elotuzumab Plus Pomalidomide and Dexamethasone for Relapsed/Refractory Multiple Myeloma: Final Overall Survival Analysis from the Randomized Phase II ELOQUENT-3 Trial. J. Clin. Oncol..

[42] Hoy S.M. (2020). Tafasitamab: First Approval. Drugs.

[43] Center for Drug Evaluation and Research (2025). FDA Approves Tafasitamab-Cxix for Relapsed or Refractory Follicular Lymphoma.

[44] Lenz G., Trněný M., Burke J.M., Nowakowski G.S., Fox C.P., Chiappella A., Dull J., Jeon Y., Cheah C., Westin J. (2026). frontMIND: Phase 3 Study of Tafasitamab (Tafa) plus Lenalidomide (Len) and R-CHOP for Patients (Pts) with Newly Diagnosed Diffuse Large B-Cell Lymphoma (DLBCL). J. Clin. Oncol..

[45] Cuesta-Mateos C., Alcaraz-Serna A., Somovilla-Crespo B., Muñoz-Calleja C. (2018). Monoclonal Antibody Therapies for Hematological Malignancies: Not Just Lineage-Specific Targets. Front. Immunol..

[46] Czuczman M.S., Leonard J.P., Jung S., Johnson J.L., Hsi E.D., Byrd J.C., Cheson B.D. (2012). Phase II Trial of Galiximab (Anti-CD80 Monoclonal Antibody) plus Rituximab (CALGB 50402): Follicular Lymphoma International Prognostic Index (FLIPI) Score Is Predictive of Upfront Immunotherapy Responsiveness. Ann. Oncol..

[47] Ansell S.M., Horwitz S.M., Engert A., Khan K.D., Lin T., Strair R., Keler T., Graziano R., Blanset D., Yellin M. (2007). Phase I/II Study of an Anti-CD30 Monoclonal Antibody (MDX-060) in Hodgkin’s Lymphoma and Anaplastic Large-Cell Lymphoma. J. Clin. Oncol..

[48] Salomon R., Dahan R. (2022). Next Generation CD40 Agonistic Antibodies for Cancer Immunotherapy. Front. Immunol..

[49] Haran M., Mirkin V., Braester A., Harpaz N., Shevetz O., Shtreiter M., Greenberg S., Mordich O., Amram O., Binsky-Ehrenreich I. (2018). A Phase I-II Clinical Trial of the Anti-CD74 Monoclonal Antibody Milatuzumab in Frail Patients with Refractory Chronic Lymphocytic Leukaemia: A Patient Based Approach. Br. J. Haematol..

[50] Read, 5 Min Immunomedics, Inc.’s Milatuzumab Receives FDA Orphan Drug Designation for Therapy of Multiple Myeloma. https://www.biospace.com/immunomedics-inc-s-milatuzumab-receives-fda-orphan-drug-designation-for-therapy-of-multiple-myeloma.

[51] Abedin S.M., Guru Murthy G.S., Hamadani M., Michaelis L.C., Carlson K.-S., Runaas L., Gauger K., Desai A.G., Chen M.M., Li K.L. (2025). Phase 1 Study of Lintuzumab-Ac225 Combined with CLAG-M Salvage Therapy in Relapsed/Refractory Acute Myeloid Leukemia. Leukemia.

[52] Grant B.W., Jung S.-H., Johnson J.L., Kostakoglu L., His E., Byrd J.C., Jones J., Leonard J.P., Martin S.E., Cheson B.D. (2013). A Phase II Trial of Extended Induction Epratuzumab and Rituximab for Previously Untreated Follicular Lymphoma: CALGB 50701. Cancer.

[53] Immunomedics Announces Termination of Epratuzumab Licensing Agreement with UCB—FirstWord Pharma. https://firstwordpharma.com/story/3586256.

[54] Zelenetz A.D., Gordon L.I., Abramson J.S., Advani R.H., Andreadis B., Bartlett N.L., Budde L.E., Caimi P.F., Chang J.E., Christian B. (2023). NCCN Guidelines^®^ Insights: B-Cell Lymphomas, Version 6.2023: Featured Updates to the NCCN Guidelines. J. Natl. Compr. Cancer Netw..

[55] Wierda W.G., Brown J., Abramson J.S., Awan F., Bociek G., Boyer D., Cortese M., Cripe L., D’Angelo C., Darnell E. (2026). NCCN Guidelines^®^ Insights: Chronic Lymphocytic Leukemia/Small Lymphocytic Lymphoma, Version 2.2026: Featured Updates to the NCCN Guidelines. J. Natl. Compr. Cancer Netw..

[56] Chaudhry M., Cheson B.D. (2015). What Is the Status of Novel Anti-CD20 Antibodies for Chronic Lymphocytic Leukemia and Are They Set to Leave Rituximab in the Shadows?. Expert Rev. Hematol..

[57] Kumar S.K., Callander N.S., Adekola K., Anderson L.D., Baljevic M., Baz R., Campagnaro E., Costello C., D’Angelo C., Derman B. (2025). NCCN Guidelines^®^ Insights: Multiple Myeloma, Version 1.2025. J. Natl. Compr. Cancer Netw..

[58] Nedved A., Maddocks K., Nowakowski G.S. (2023). Clinical Treatment Guidelines for Tafasitamab Plus Lenalidomide in Patients with Relapsed or Refractory Diffuse Large B-Cell Lymphoma. Oncologist.

[59] Zelenetz A.D., Gordon L.I., Abramson J.S., Advani R.H., Andreadis B., Bartlett N.L., Budde L.E., Caimi P.F., Chang J.E., Christian B. (2025). NCCN Guidelines^®^ Insights: B-Cell Lymphomas 3.2025. J. Natl. Compr. Cancer Netw..

[60] Biogen (2015). A Phase III, Randomized, Double-Blind Study of Galiximab in Combination with Rituximab Compared with Rituximab in Combination with Placebo for the Treatment of Subjects with Relapsed or Refractory, Follicular Non-Hodgkin’s Lymphoma.

[61] Przepiorka D., Ko C.-W., Deisseroth A., Yancey C.L., Candau-Chacon R., Chiu H.-J., Gehrke B.J., Gomez-Broughton C., Kane R.C., Kirshner S. (2015). FDA Approval: Blinatumomab. Clin. Cancer Res..

[62] Center for Drug Evaluation and Research (2024). FDA Approves Blinatumomab as Consolidation for CD19-Positive Philadelphia Chromosome-Negative B-Cell Precursor Acute Lymphoblastic Leukemia.

[63] Litzow M.R., Sun Z., Mattison R.J., Paietta E.M., Roberts K.G., Zhang Y., Racevskis J., Lazarus H.M., Rowe J.M., Arber D.A. (2024). Blinatumomab for MRD-Negative Acute Lymphoblastic Leukemia in Adults. N. Engl. J. Med..

[64] Locatelli F., Zugmaier G., Rizzari C., Morris J.D., Gruhn B., Klingebiel T., Parasole R., Linderkamp C., Flotho C., Petit A. (2021). Effect of Blinatumomab vs Chemotherapy on Event-Free Survival Among Children with High-Risk First-Relapse B-Cell Acute Lymphoblastic Leukemia: A Randomized Clinical Trial. JAMA.

[65] Hogan L.E., Brown P.A., Ji L., Xu X., Devidas M., Bhatla T., Borowitz M.J., Raetz E.A., Carroll A., Heerema N.A. (2023). Children’s Oncology Group AALL1331: Phase III Trial of Blinatumomab in Children, Adolescents, and Young Adults with Low-Risk B-Cell ALL in First Relapse. J. Clin. Oncol..

[66] Foà R., Bassan R., Elia L., Piciocchi A., Soddu S., Messina M., Ferrara F., Lunghi M., Mulè A., Bonifacio M. (2024). Long-Term Results of the Dasatinib-Blinatumomab Protocol for Adult Philadelphia-Positive ALL. J. Clin. Oncol..

[67] Kantarjian H., Short N.J., Haddad F.G., Jain N., Huang X., Montalban-Bravo G., Kanagal-Shamanna R., Kadia T.M., Daver N., Chien K. (2024). Results of the Simultaneous Combination of Ponatinib and Blinatumomab in Philadelphia Chromosome-Positive ALL. J. Clin. Oncol..

[68] Chiaretti S., Di Trani M., Skert C., Elia L., Almici G., Della Starza I., Cardinali D., Bellomarino V., Soddu S., Messina M. (2025). First Results of the Phase III GIMEMA ALL2820 Trial Comparing Ponatinib plus Blinatumomab to Imatinib and Chemotherapy for Newly Diagnosed Adult Ph+ Acute Lymphoblastic Leukemia Patients. Blood.

[69] Tuscano J.M., Othman T., Frankel P., Ruel C., Poh C., Herbert S., Maverakis E., Merleev A.A., Luxardi G., Abedi M. (2026). A Phase 1 Study of Blinatumomab/Lenalidomide in Relapsed/Refractory B-Cell Lymphoma: Toxicity, Efficacy, and Correlative Analysis. Blood Adv..

[70] Topp M., Dlugosz-Danecka M., Skotnicki A.B., Salogub G., Viardot A., Klein A.K., Hess G., Michel C.S., Grosicki S., Gural A. (2023). Safety of AFM11 in the Treatment of Patients with B-Cell Malignancies: Findings from Two Phase 1 Studies. Trials.

[71] BLINCYTO^®^ (Blinatumomab) Clinical Data—Pediatric & AYA 20120215 Study. https://www.blincytohcp.com/clinical-data/pediatric-aya/20120215-study.

[72] Gupta S., Rau R.E., Kairalla J.A., Rabin K.R., Wang C., Angiolillo A.L., Alexander S., Carroll A.J., Conway S., Gore L. (2025). Blinatumomab in Standard-Risk B-Cell Acute Lymphoblastic Leukemia in Children. N. Engl. J. Med..

[73] Kang C. (2022). Mosunetuzumab: First Approval. Drugs.

[74] Sehn L.H., Bartlett N.L., Matasar M.J., Schuster S.J., Assouline S.E., Giri P., Kuruvilla J., Shadman M., Cheah C.Y., Dietrich S. (2025). Long-Term 3-Year Follow-up of Mosunetuzumab in Relapsed or Refractory Follicular Lymphoma after ≥2 Prior Therapies. Blood.

[75] Bartlett N.L., Sehn L.H., Assouline S., Giri P., Kuruvilla J., Schuster S.J., Yoon S.-S., Fay K., Hess G., Dreyling M. (2026). Fixed-Duration Subcutaneous Mosunetuzumab in Relapsed/Refractory Follicular Lymphoma: Pivotal Phase 2 Primary Analysis. Am. J. Hematol..

[76] Budde L.E., Assouline S., Sehn L.H., Schuster S.J., Yoon S.-S., Yoon D.H., Matasar M.J., Bosch F., Kim W.S., Nastoupil L.J. (2024). Durable Responses with Mosunetuzumab in Relapsed/Refractory Indolent and Aggressive B-Cell Non-Hodgkin Lymphomas: Extended Follow-Up of a Phase I/II Study. J. Clin. Oncol..

[77] Alderuccio J.P., Okada C., Crochet G., Ayers E.C., Hu M., Ferrari S., Caimi P.F., Hamadani M., Depaus J., Derenzini E. (2025). ABCL-777: Initial Results from LOTIS-7: A Phase 1b Study of Loncastuximab Tesirine Plus Glofitamab in Patients with Relapsed/Refractory (R/R) Diffuse Large B-Cell Lymphoma (DLBCL). Clin. Lymphoma Myeloma Leuk..

[78] Hutchings M., Sureda A., Bosch F., Larsen T.S., Corradini P., Avigdor A., Terol M.J., Dominguez A.R., Pinto A., Skarbnik A. (2025). Efficacy and Safety of Glofitamab Plus Polatuzumab Vedotin in Relapsed/Refractory Large B-Cell Lymphoma Including High-Grade B-Cell Lymphoma: Results from a Phase Ib/II Trial. J. Clin. Oncol..

[79] Minson A., Verner E., Giri P., Butler J., Janowski W., Cheah C.Y., Ratnasingam S., Wong S.M., Ku M., Hertzberg M. (2025). Glofitamab Combined with Pola-R-CHP or R-CHOP as First Therapy in Younger Patients with High-Risk Large B-Cell Lymphoma: Results from the COALITION Study. J. Clin. Oncol..

[80] Cartron G., Houot R., Al Tabaa Y., Le Bras F., Ysebaert L., Choquet S., Jardin F., Bay J.-O., Gros F.-X., Morschhauser F. (2025). Glofitamab in Refractory or Relapsed Diffuse Large B Cell Lymphoma after Failing CAR-T Cell Therapy: A Phase 2 LYSA Study. Nat. Cancer.

[81] Eyre T.A., Cwynarski K., d’Amore F., Leval L.d., Dreyling M., Eichenauer D.A., Ferreri A.J.M., Giné E., Kersten M.J., Ladetto M. (2025). Lymphomas: ESMO Clinical Practice Guideline for Diagnosis, Treatment and Follow-Up. Ann. Oncol..

[82] Dickinson M.J., Carlo-Stella C., Morschhauser F., Bachy E., Corradini P., Iacoboni G., Khan C., Wróbel T., Offner F., Trněný M. (2022). Glofitamab for Relapsed or Refractory Diffuse Large B-Cell Lymphoma. N. Engl. J. Med..

[83] Center for Drug Evaluation and Research (2025). FDA Approves Epcoritamab-Bysp for Follicular Lymphoma Indications.

[84] Falchi L., Nijland M., Huang H., Linton K.M., Seymour J.F., Tao R., Kwiatek M., Costa A., Vassilakopoulos T.P., Greil R. (2026). Epcoritamab, Lenalidomide, and Rituximab versus Lenalidomide and Rituximab for Relapsed or Refractory Follicular Lymphoma (EPCORE FL-1): A Global, Open-Label, Randomised, Phase 3 Trial. Lancet.

[85] Odronextamab BLA Accepted for FDA Review for the Treatment of Relapsed/Refractory Follicular Lymphoma—Regeneron Pharmaceuticals Inc. https://investor.regeneron.com/news-releases/news-release-details/odronextamab-bla-accepted-fda-review-treatment/.

[86] Kim T.M., Taszner M., Novelli S., Cho S.-G., Villasboas J.C., Merli M., Jiménez-Ubieto A., Tessoulin B., Poon L.M., Tucker D. (2024). Safety and Efficacy of Odronextamab in Patients with Relapsed or Refractory Follicular Lymphoma. Ann. Oncol..

[87] Michot J.-M., Yagci M., Kargus K., Mohamed H., Chen J., Brouwer-Visser J., Chaudhry A., Iqbal N., Matasar M. (2025). Odronextamab plus Chemotherapy in Patients with Previously Untreated Diffuse Large B-Cell Lymphoma (DLBCL): First Results from Part 1 of the Phase 3 Olympia-3 Study. Blood.

[88] Topp M.S., Matasar M., Allan J.N., Ansell S.M., Barnes J.A., Arnason J.E., Michot J.-M., Goldschmidt N., O’Brien S.M., Abadi U. (2025). Odronextamab Monotherapy in R/R DLBCL after Progression with CAR T-Cell Therapy: Primary Analysis of the ELM-1 Study. Blood.

[89] Riedell P.A., Patel K., Dunleavy V., Karimi Y.H., Shah N.N., Ribrag V., Ysebaert L., Noel R., Brisou G., Kline J. (2024). Plamotamab: First Presentation of Subcutaneous Administration in a Phase 1 Dose Escalation Study in Heavily Pretreated R/R NHL Patients Who Had Prior CAR-T Cell Therapy. Blood.

[90] Xencor, Inc. (2024). A Phase 2 Randomized, Open-Label, Multicenter Study to Evaluate the Efficacy and Safety of XmAb13676 (Plamotamab) Combined with Tafasitamab Plus Lenalidomide Versus Tafasitamab Plus Lenalidomide in Subjects with Relapsed or Refractory Diffuse Large B-Cell Lymphoma.

[91] Baines A.C., Kanapuru B., Zhao J., Price L.S.L., Zheng N., Konicki R., Manning M.L., Gehrke B.J., Theoret M.R., Gormley N.J. (2024). FDA Approval Summary: Teclistamab–A Bispecific CD3 T-Cell Engager for Patients with Relapsed or Refractory Multiple Myeloma. Clin. Cancer Res..

[92] Center for Drug Evaluation and Research (2026). FDA Approves Teclistamab in Combination with Daratumumab Hyaluronidase-Fihj for Relapsed or Refractory Multiple Myeloma.

[93] TECVAYLI^®^▼ (Teclistamab) Monotherapy Application Submitted to the EMA for Relapsed/Refractory Multiple Myeloma After at Least One Prior Therapy. https://www.jnj.com/media-center/press-releases/tecvayli-teclistamab-monotherapy-application-submitted-to-the-ema-for-relapsed-refractory-multiple-myeloma-after-at-least-one-prior-therapy.

[94] Zamagni E., Silzle T., Špička I., Tahri S., Lonergan S., Nijhof I.S., Falcone A.P., Terpos E., Radocha J., Mina R. (2024). Phase 3 Study of Teclistamab (Tec) in Combination with Lenalidomide (Len) and Tec Alone Versus Len Alone in Newly Diagnosed Multiple Myeloma (NDMM) As Maintenance Therapy Following Autologous Stem Cell Transplantation (ASCT): Safety Run-in (SRI) Results from the Majestec-4/EMN30 Trial. Blood.

[95] Chen W., Cai Z., Chim J.C., Chng W.J., Du J., Fu C., Hanamura I., Hou J., Huang J.S.-Y., Ishida T. (2025). Consensus Guidelines and Recommendations for The CD38 Monoclonal Antibody-Based Quadruplet Therapy and Management in Clinical Practice for Newly Diagnosed Multiple Myeloma: From the Pan-Pacific Multiple Myeloma Working Group. Clin. Hematol. Int..

[96] Center for Drug Evaluation and Research (2025). FDA Grants Accelerated Approval to Linvoseltamab-Gcpt for Relapsed or Refractory Multiple Myeloma.

[97] Lee A. (2025). Linvoseltamab: First Approval. Drugs.

[98] Lee H.C., Zonder J.A., Dhodapkar M.V., Jagannath S., Hoffman J.E., Suvannasankha A., Shah M.R., Lentzsch S., Baz R., Maly J.J. (2026). Linvoseltamab in Patients with Relapsed/Refractory Multiple Myeloma in the LINKER-MM1 Study: Longer Follow-Up and Subgroup Analyses. Clin. Lymphoma Myeloma Leuk..

[99] Weisel K., Hungria V., Quach H., Yoon S.-S., Rodríguez-Otero P., Kroog G., Sinha A., Boyapati A., Lyon C., Lorenc K.R. (2023). PB2133: Trial in Progress: Linker-Mm3, a Phase 3, Open-Label, Randomized Study of Linvoseltamab Versus Elotuzumab, Pomalidomide, and Dexamethasone (EPD) in Relapsed/Refractory Multiple Myeloma (RRMM). Hemasphere.

[100] Treatment of Multiple Myeloma: ASCO Living Guideline—ASCO Publications. https://ascopubs.org/guidelines/treatment-multiple-myeloma-asco-living-guideline?doi=10.5555%2Fguidelines&publicationCode=guidelines.

[101] Lesokhin A.M., Tomasson M.H., Arnulf B., Bahlis N.J., Miles Prince H., Niesvizky R., Rodrίguez-Otero P., Martinez-Lopez J., Koehne G., Touzeau C. (2023). Elranatamab in Relapsed or Refractory Multiple Myeloma: Phase 2 MagnetisMM-3 Trial Results. Nat. Med..

[102] Dhillon S. (2023). Elranatamab: First Approval. Drugs.

[103] Center for Drug Evaluation and Research (2023). FDA Grants Accelerated Approval to Elranatamab-Bcmm for Multiple Myeloma.

[104] Bar N., Martin T., Hofmeister C.C., Mateos M.-V., Hansson M., Paris L., Namburi S., Ribas P., Santoro A., Rodriguez-Otero P. (2026). Alnuctamab, a Bivalent B-Cell Maturation Antigen-Targeting T Cell Engager for Patients with Relapsed or Refractory Multiple Myeloma: Results from a Phase 1, First-in-Human Study. Leukemia.

[105] Celgene (2026). A Phase 1b/2a, Multicenter, Open-Label Study to Determine the Recommended Dose and Schedule, and Evaluate the Safety and Preliminary Efficacy of Alnuctamab in Combination with Mezigdomide in Participants with Relapsed and/or Refractory Multiple Myeloma.

[106] D’Souza A., Shah N., Rodriguez C., Voorhees P.M., Weisel K., Bueno O.F., Pothacamury R.K., Freise K.J., Yue S., Ross J.A. (2022). A Phase I First-in-Human Study of ABBV-383, a B-Cell Maturation Antigen × CD3 Bispecific T-Cell Redirecting Antibody, in Patients with Relapsed/Refractory Multiple Myeloma. J. Clin. Oncol..

[107] Pouleau B., Estoppey C., Suere P., Nallet E., Laurendon A., Monney T., Pais Ferreira D., Drake A., Carretero-Iglesia L., Macoin J. (2023). Preclinical Characterization of ISB 1342, a CD38 × CD3 T-Cell Engager for Relapsed/Refractory Multiple Myeloma. Blood.

[108] Kapoor P., Stevens D.A., Leleu X., Belhadj Merzoug K., Karlin L., Manier S., Chalopin T., Hulin C., Perrot A., Berdeja J.G. (2023). Dose Escalation of ISB 1342, a Novel CD38 × CD3 Bispecific Antibody, in Patients with Relapsed/Refractory Multiple Myeloma (RRMM). Blood.

[109] Keam S.J. (2023). Talquetamab: First Approval. Drugs.

[110] Chari A., Touzeau C., Schinke C., Minnema M.C., Berdeja J.G., Oriol A., van de Donk N.W.C.J., Rodríguez-Otero P., Morillo D., Martinez-Chamorro C. (2025). Safety and Activity of Talquetamab in Patients with Relapsed or Refractory Multiple Myeloma (MonumenTAL-1): A Multicentre, Open-Label, Phase 1–2 Study. Lancet Haematol..

[111] Cohen Y.C., Magen H., Gatt M., Sebag M., Kim K., Min C.-K., Ocio E.M., Yoon S.-S., Chu M.P., Rodríguez-Otero P. (2025). Talquetamab plus Teclistamab in Relapsed or Refractory Multiple Myeloma. N. Engl. J. Med..

[112] Richter J., Thomas S.K., Krishnan A.Y., Laubach J.P., Cohen A.D., Trudel S., Costa L.J., Bahlis N.J., Forsberg P.A., Kaedbey R. (2024). Cevostamab in Patients with Heavily Pretreated Relapsed/Refractory Multiple Myeloma (RRMM): Updated Results from an Ongoing Phase I Study Demonstrate Clinically Meaningful Activity and Manageable Safety and Inform the Doses and Regimen for Combination Studies. Blood.

[113] Zhang Q., Song Y., Zhou K., Lin Q. (2025). Emerging Therapeutic Agents in Multiple Myeloma: Highlights from the 2024 ASH Annual Meeting. Biomark. Res..

[114] Delforge M., Richter J., Cohen Y.C., Corradini P., Sborov D.W., Lesokhin A.M., Berdeja J.G., Gatt M.E., Paris L., Rosinol Dachs L. (2024). Biomarker Correlates and Clinical Activity of Cevostamab in Patients (Pts) with Triple-Class Refractory Multiple Myeloma (MM) Who Have Received ≥1 Prior B-Cell Maturation Antigen (BCMA)-Targeted Bispecific Antibody (BsAb): Results from the Phase I/II CAMMA 2 Study. Blood.

[115] Uy G.L., Aldoss I., Foster M.C., Sayre P.H., Wieduwilt M.J., Advani A.S., Godwin J.E., Arellano M.L., Sweet K.L., Emadi A. (2021). Flotetuzumab as Salvage Immunotherapy for Refractory Acute Myeloid Leukemia. Blood.

[116] Ravandi F., Bashey A., Foran J., Stock W., Mawad R., Short N., Yilmaz M., Kantarjian H., Odenike O., Patel A. (2023). Phase 1 Study of Vibecotamab Identifies an Optimized Dose for Treatment of Relapsed/Refractory Acute Myeloid Leukemia. Blood Adv..

[117] Boyiadzis M., Desai P., Daskalakis N., Donnellan W., Ferrante L., Goldberg J.D., Grunwald M.R., Guttke C., Li X., Perez-Simon J.A. (2023). First-in-Human Study of JNJ-63709178, a CD123/CD3 Targeting Antibody, in Relapsed/Refractory Acute Myeloid Leukemia. Clin. Transl. Sci..

[118] Schmitt N., Siegler J.-J., Beck A., Müller T., Kozlowska I., Sarlang S., Reusch U., Knackmuss S., Medina-Echeverz J., Koch J. (2025). The Bispecific Innate Cell Engager AFM28 Eliminates CD123+ Leukemic Stem and Progenitor Cells in AML and MDS. Nat. Commun..

[119] Gökbuget N., Boissel N., Chiaretti S., Dombret H., Doubek M., Fielding A., Foà R., Giebel S., Hoelzer D., Hunault M. (2024). Management of ALL in Adults: 2024 ELN Recommendations from a European Expert Panel. Blood.

[120] Hoelzer D., Bassan R., Boissel N., Roddie C., Ribera J.M., Jerkeman M. (2024). ESMO Clinical Practice Guideline Interim Update on the Use of Targeted Therapy in Acute Lymphoblastic Leukaemia. Ann. Oncol..

[121] Tapia-Galisteo A., Álvarez-Vallina L., Sanz L. (2023). Bi- and Trispecific Immune Cell Engagers for Immunotherapy of Hematological Malignancies. J. Hematol. Oncol..

[122] Schjesvold F., Jelinek T., Polgarova K., Pour L., Yoon S.-S., Kim W.S., Fosså A., San-Miguel J.F., Canales M., Rodríguez-Otero P. (2024). First-in-Human Phase 1 Study of SAR442257 in Patients with Relapsed/Refractory Multiple Myeloma and Non-Hodgkin Lymphoma. Blood.

[123] Pillarisetti K., Yang D., Luistro L., Yao J., Smith M., Vulfson P., Testa J.S., Ponticiello R., Brodeur S., Heidrich B. (2026). Ramantamig (JNJ-79635322), a Novel T-Cell-Engaging Trispecific Antibody Targeting BCMA, GPRC5D, and CD3, in Multiple Myeloma Models. Blood.

[124] Innovent Biologics ASH 2025 Oral Presentation: Innovent Biologics Announces Initial Results of the First-in-Human Phase 1 Study of Trispecific Antibody IBI3003 in Relapsed or Refractory Multiple Myeloma. https://www.prnewswire.com/news-releases/ash-2025-oral-presentation-innovent-biologics-announces-initial-results-of-the-first-in-human-phase-1-study-of-trispecific-antibody-ibi3003-in-relapsed-or-refractory-multiple-myeloma-302634871.html.

[125] Kuchnio A., Yang D., Vloemans N., Cornelissen I., Amorim R., Perova T., Lowenstein C., Janssen L., Suls T., Bekkers M. (2026). Characterization of JNJ-80948543 a Novel CD79bxCD20xCD3 Trispecific Antibody for B-Cell Non-Hodgkin Lymphoma. Haematologica.

[126] Overview: GT Biopharma, Inc. (GTBP). https://www.gtbiopharma.com/product-pipeline/overview.

[127] Juckett M., Graham C., Grover P., Norton J., Premnath N., Eckfeldt C., Sachs Z., Seichter C., Hoeschen A., McDonald-Hyman C. (2025). Gtb-3650 (Anti-CD16/IL-15/Anti-CD33) Tri-Specific Killer Engager (TriKE^®^) for the Treatment of High Risk Myelodysplastic Syndromes (MDS) and Refractory/Relapsed (R/R) Acute Myeloid Leukemia (AML) (NCT06594445). Blood.

[128] GT Biopharma, Inc. (2022). GTB-3550 (CD16/IL-15/CD33)Tri-Specific Killer Engager (TriKE^®^) for the Treatment of High Risk Myelodysplastic Syndromes, Refractory/Relapsed Acute Myeloid Leukemia and Advanced Systemic Mastocytosis.

[129] Kandari D., Bhatnagar R. (2021). Antibody Engineering and Its Therapeutic Applications. Int. Rev. Immunol..

[130] Lambert J., Pautas C., Terré C., Raffoux E., Turlure P., Caillot D., Legrand O., Thomas X., Gardin C., Gogat-Marchant K. (2019). Gemtuzumab Ozogamicin for de Novo Acute Myeloid Leukemia: Final Efficacy and Safety Updates from the Open-Label, Phase III ALFA-0701 Trial. Haematologica.

[131] Petersdorf S.H., Kopecky K.J., Slovak M., Willman C., Nevill T., Brandwein J., Larson R.A., Erba H.P., Stiff P.J., Stuart R.K. (2013). A Phase 3 Study of Gemtuzumab Ozogamicin during Induction and Postconsolidation Therapy in Younger Patients with Acute Myeloid Leukemia. Blood.

[132] Norsworthy K.J., Ko C., Lee J.E., Liu J., John C.S., Przepiorka D., Farrell A.T., Pazdur R. (2018). FDA Approval Summary: Mylotarg for Treatment of Patients with Relapsed or Refractory CD33-Positive Acute Myeloid Leukemia. Oncologist.

[133] Pollard J.A., Guest E., Alonzo T.A., Gerbing R.B., Loken M.R., Brodersen L.E., Kolb E.A., Aplenc R., Meshinchi S., Raimondi S.C. (2021). Gemtuzumab Ozogamicin Improves Event-Free Survival and Reduces Relapse in Pediatric KMT2A-Rearranged AML: Results from the Phase III Children’s Oncology Group Trial AAML0531. J. Clin. Oncol..

[134] Center for Drug Evaluation and Research (2024). FDA Approves Gemtuzumab Ozogamicin for CD33-Positive AML in Pediatric Patients.

[135] Tomizawa D., Tsujimoto S., Tanaka S., Matsubayashi J., Aoki T., Iwamoto S., Hasegawa D., Nagai K., Nakashima K., Kawaguchi K. (2022). A Phase III Clinical Trial Evaluating Efficacy and Safety of Minimal Residual Disease-Based Risk Stratification for Children with Acute Myeloid Leukemia, Incorporating a Randomized Study of Gemtuzumab Ozogamicin in Combination with Post-Induction Chemotherapy for Non-Low-Risk Patients (JPLSG-AML-20). Jpn. J. Clin. Oncol..

[136] Kovtun Y., Noordhuis P., Whiteman K.R., Watkins K., Jones G.E., Harvey L., Lai K.C., Portwood S., Adams S., Sloss C.M. (2018). IMGN779, a Novel CD33-Targeting Antibody-Drug Conjugate with DNA-Alkylating Activity, Exhibits Potent Antitumor Activity in Models of AML. Mol. Cancer Ther..

[137] Pollyea D.A., Bixby D., Perl A., Bhatt V.R., Altman J.K., Appelbaum F.R., Lima M.d., Fathi A.T., Foran J.M., Gojo I. (2021). NCCN Guidelines Insights: Acute Myeloid Leukemia, Version 2.2021: Featured Updates to the NCCN Guidelines. J. Natl. Compr. Cancer Netw..

[138] Ibritumomab Tiuxetan—An Overview—ScienceDirect Topics. https://www.sciencedirect.com/topics/medicine-and-dentistry/ibritumomab-tiuxetan.

[139] Grillo-López A.J. (2002). Zevalin: The First Radioimmunotherapy Approved for the Treatment of Lymphoma. Expert Rev. Anticancer. Ther..

[140] Zevalin—European Medicines Agency (EMA). https://www.ema.europa.eu/en/medicines/human/EPAR/zevalin.

[141] Stenger M. FDA Approves Brentuximab Vedotin in Two Lymphoma Indications. https://ascopost.com/issues/september-15-2011/fda-approves-brentuximab-vedotin-in-two-lymphoma-indications/.

[142] Stenger M. Brentuximab Vedotin for Consolidation Therapy in High-Risk Hodgkin Lymphoma. https://ascopost.com/issues/september-10-2015/brentuximab-vedotin-for-consolidation-therapy-in-high-risk-hodgkin-lymphoma/.

[143] Moskowitz C.H., Walewski J., Nademanee A., Masszi T., Agura E., Holowiecki J., Abidi M.H., Chen A.I., Stiff P., Viviani S. (2018). Five-Year PFS from the AETHERA Trial of Brentuximab Vedotin for Hodgkin Lymphoma at High Risk of Progression or Relapse. Blood.

[144] Center for Drug Evaluation and Research (2019). FDA Approves Brentuximab Vedotin for Previously Untreated sALCL and CD30-Expressing PTCL.

[145] Horwitz S., O’Connor O.A., Pro B., Illidge T., Fanale M., Advani R., Bartlett N.L., Christensen J.H., Morschhauser F., Domingo-Domenech E. (2019). Department of Error. Lancet.

[146] Ansell S.M., Radford J., Connors J.M., Długosz-Danecka M., Kim W.-S., Gallamini A., Ramchandren R., Friedberg J.W., Advani R., Hutchings M. (2022). Overall Survival with Brentuximab Vedotin in Stage III or IV Hodgkin’s Lymphoma. N. Engl. J. Med..

[147] Center for Drug Evaluation and Research (2025). FDA Approves Brentuximab Vedotin with Lenalidomide and Rituximab for Relapsed or Refractory Large B-Cell Lymphoma.

[148] Bartlett N.L., Hahn U., Kim W.-S., Fleury I., Laribi K., Bergua J.-M., Bouabdallah K., Forward N., Bijou F., MacDonald D. (2025). Brentuximab Vedotin Combination for Relapsed Diffuse Large B-Cell Lymphoma. J. Clin. Oncol..

[149] Center for Drug Evaluation and Research (2024). FDA D.I.S.C.O. Burst Edition: FDA Approval of Adcetris (Brentuximab Vedotin) in Combination with Chemotherapy for Pediatric Patients with Classical Hodgkin Lymphoma.

[150] Castellino S.M., Pei Q., Parsons S.K., Hodgson D., McCarten K., Horton T., Cho S., Wu Y., Punnett A., Dave H. (2022). Brentuximab Vedotin with Chemotherapy in Pediatric High-Risk Hodgkin’s Lymphoma. N. Engl. J. Med..

[151] Friedberg J.W., Bordoni R., Patel-Donnelly D., Larson T., Goldschmidt J., Boccia R., Cline V.J.M., Mamidipalli A., Liu J., Akyol A. (2024). Brentuximab Vedotin with Dacarbazine or Nivolumab as Frontline cHL Therapy for Older Patients Ineligible for Chemotherapy. Blood.

[152] Herrera A.F., LeBlanc M., Castellino S.M., Li H., Rutherford S.C., Evens A.M., Davison K., Punnett A., Parsons S.K., Ahmed S. (2024). Nivolumab+AVD in Advanced-Stage Classic Hodgkin’s Lymphoma. N. Engl. J. Med..

[153] Herrera A.F., Zain J., Savage K.J., Feldman T., Brammer J.E., Chen L., Puverel S., Popplewell L., Budde L.E., Mei M. (2024). Brentuximab Vedotin plus Cyclophosphamide, Doxorubicin, Etoposide, and Prednisone Followed by Brentuximab Vedotin Consolidation in CD30-Positive Peripheral T-Cell Lymphomas: A Multicentre, Single-Arm, Phase 2 Study. Lancet Haematol..

[154] Advani R.H., Kelsey C.R., Armand P., Bello C.M., Benitez C.M., Bond D., Chen W., Cherian S., Czader M., Dabaja B. (2026). Hodgkin Lymphoma, Version 1.2026, NCCN Clinical Practice Guidelines In Oncology. J. Natl. Compr. Cancer Netw..

[155] Horwitz S., O’Connor O.A., Pro B., Illidge T., Fanale M., Advani R., Bartlett N.L., Christensen J.H., Morschhauser F., Domingo-Domenech E. (2019). Brentuximab Vedotin with Chemotherapy for CD30-Positive Peripheral T-Cell Lymphoma (ECHELON-2): A Global, Double-Blind, Randomised, Phase 3 Trial. Lancet.

[156] FDA Approves Inotuzumab Ozogamicin (Besponsa) for ALL. https://www.medscape.com/viewarticle/884434.

[157] Zhao Y., Laird A.D., Roberts K.G., Yafawi R., Kantarjian H., DeAngelo D.J., Stelljes M., Liedtke M., Stock W., Gökbuget N. (2024). Association of Leukemic Molecular Profile with Efficacy of Inotuzumab Ozogamicin in Adults with Relapsed/Refractory ALL. Blood Adv..

[158] Center for Drug Evaluation and Research (2024). FDA Approves Inotuzumab Ozogamicin for Pediatric Patients with Acute Lymphoblastic Leukemia.

[159] Stelljes M., Raffel S., Alakel N., Wäsch R., Kondakci M., Scholl S., Rank A., Hänel M., Spriewald B., Hanoun M. (2024). Inotuzumab Ozogamicin as Induction Therapy for Patients Older Than 55 Years with Philadelphia Chromosome-Negative B-Precursor ALL. J. Clin. Oncol..

[160] Jabbour E.J., Rousselot P., Gokbuget N., Chevallier P., Kantarjian H.M., Stelljes M. (2025). Inotuzumab Ozogamicin as First-Line Therapy in Acute Lymphoblastic Leukemia. Clin. Lymphoma Myeloma Leuk..

[161] Shah B., Mattison R.J., Abboud R., Abdelmessieh P., Aldoss I., Burke P.W., DeAngelo D.J., Dinner S., Fathi A.T., Gauthier J. (2024). Acute Lymphoblastic Leukemia, Version 2.2024, NCCN Clinical Practice Guidelines in Oncology. J. Natl. Compr. Cancer Netw..

[162] Center for Drug Evaluation and Research (2020). FDA D.I.S.C.O.: FDA Approval of Moxetumomab Pasudotox-Tdfk for Relapsed or Refractory Hairy Cell Leukemia.

[163] Moxetumomab Approved for Hairy Cell Leukemia—NCI. https://www.cancer.gov/news-events/cancer-currents-blog/2018/moxetumomab-fda-hairy-cell-leukemia.

[164] Lumoxiti—European Medicines Agency (EMA). https://www.ema.europa.eu/en/medicines/human/EPAR/lumoxiti.

[165] O’Brien M.M., Ji L., Shah N.N., Rheingold S.R., Bhojwani D., Yuan C.M., Xu X., Yi J.S., Harris A.C., Brown P.A. (2022). Phase II Trial of Inotuzumab Ozogamicin in Children and Adolescents with Relapsed or Refractory B-Cell Acute Lymphoblastic Leukemia: Children’s Oncology Group Protocol AALL1621. J. Clin. Oncol..

[166] Chevallier P., Leguay T., Delord M., Salek C., Kim R., Huguet F., Hicheri Y., Wartiovaara-Kautto U., Raffoux E., Cluzeau T. (2024). Inotuzumab Ozogamicin and Low-Intensity Chemotherapy in Older Patients with Newly Diagnosed CD22+ Philadelphia Chromosome–Negative B-Cell Precursor Acute Lymphoblastic Leukemia. J. Clin. Oncol..

[167] Wieduwilt M.J., Yin J., Kour O., Teske R., Stock W., Byrd K., Doucette K., Mangan J., Masters G.A., Mims A.S. (2023). Chemotherapy-Free Treatment with Inotuzumab Ozogamicin and Blinatumomab for Older Adults with Newly Diagnosed, Ph-Negative, CD22-Positive, B-Cell Acute Lymphoblastic Leukemia: Alliance A041703. J. Clin. Oncol..

[168] Deeks E.D. (2019). Polatuzumab Vedotin: First Global Approval. Drugs.

[169] Sarraf Yazdy M., Kasamon Y.L., Gu W., Rodriguez L.R., Jin S., Bhatnagar V., Richardson N.C., Theoret M.R., Pazdur R., Gormley N.J. (2024). FDA Approval Summary: Polatuzumab Vedotin in the First-Line Treatment of Select Large B-Cell Lymphomas. Clin. Cancer Res..

[170] Morschhauser F., Salles G., Sehn L.H., Herrera A.F., Friedberg J.W., Trněný M., Lenz G., Sharman J.P., Herbaux C., Burke J.M. (2026). Erratum: Five-Year Outcomes of the POLARIX Study Comparing Pola-R-CHP and R-CHOP in Patients with Diffuse Large B-Cell Lymphoma. J. Clin. Oncol..

[171] Budde L.E., Zhang H., Kim W.-S., Maruyama D., Rego E.M., Norasetthada L., Hong H., Ozcan M., Jeon Y.-W., Leão Cordeiro de Farias D. (2025). Mosunetuzumab Plus Polatuzumab Vedotin in Transplant-Ineligible Refractory/Relapsed Large B-Cell Lymphoma: Primary Results of the Phase III SUNMO Trial. J. Clin. Oncol..

[172] Lee A. (2021). Loncastuximab Tesirine: First Approval. Drugs.

[173] Caimi P.F., Ai W., Alderuccio J.P., Ardeshna K.M., Hamadani M., Hess B., Kahl B.S., Radford J., Solh M., Stathis A. (2021). Loncastuximab Tesirine in Relapsed or Refractory Diffuse Large B-Cell Lymphoma (LOTIS-2): A Multicentre, Open-Label, Single-Arm, Phase 2 Trial. Lancet Oncol..

[174] Caimi P.F., Ai W.Z., Alderuccio J.P., Ardeshna K.M., Hamadani M., Hess B., Kahl B.S., Radford J., Solh M., Stathis A. (2024). Loncastuximab Tesirine in Relapsed/Refractory Diffuse Large B-Cell Lymphoma: Long-Term Efficacy and Safety from the Phase II LOTIS-2 Study. Haematologica.

[175] Jain N., Stock W., Zeidan A., Atallah E., McCloskey J., Heffner L., Tomlinson B., Bhatnagar B., Feingold J., Ungar D. (2020). Loncastuximab Tesirine, an Anti-CD19 Antibody-Drug Conjugate, in Relapsed/Refractory B-Cell Acute Lymphoblastic Leukemia. Blood Adv..

[176] Alderuccio J.P., Alencar A.J., Schatz J.H., Kuker R.A., Pongas G., Reis I.M., Lekakis L.J., Spiegel J.Y., Sandoval-Sus J., Beitinjaneh A. (2025). Loncastuximab Tesirine with Rituximab in Patients with Relapsed or Refractory Follicular Lymphoma: A Single-Centre, Single-Arm, Phase 2 Trial. Lancet Haematol..

[177] ZYNLONTA^®^ (Loncastuximab Tesirine-Lpyl)—For 3L DLBCL. https://www.zynlontahcp.com/.

[178] Lonial S., Lee H.C., Badros A., Trudel S., Nooka A.K., Chari A., Abdallah A.-O., Callander N., Lendvai N., Sborov D. (2020). Belantamab Mafodotin for Relapsed or Refractory Multiple Myeloma (DREAMM-2): A Two-Arm, Randomised, Open-Label, Phase 2 Study. Lancet Oncol..

[179] Markham A. (2020). Belantamab Mafodotin: First Approval. Drugs.

[180] Center for Drug Evaluation and Research (2024). FDA Granted Accelerated Approval to Belantamab Mafodotin-Blmf for Multiple Myeloma.

[181] Dimopoulos M.A., Hungria V.T.M., Radinoff A., Delimpasi S., Mikala G., Masszi T., Li J., Capra M., Maiolino A., Pappa V. (2023). Efficacy and Safety of Single-Agent Belantamab Mafodotin versus Pomalidomide plus Low-Dose Dexamethasone in Patients with Relapsed or Refractory Multiple Myeloma (DREAMM-3): A Phase 3, Open-Label, Randomised Study. Lancet Haematol..

[182] Blenrep (Belantamab Mafodotin) Approved in China for Treatment of 2L+ Relapsed/Refractory Multiple Myeloma—GSK. https://www.gsk.com/en-gb/media/press-releases/blenrep-belantamab-mafodotin-approved-in-china-for-treatment-of-2lplus-relapsedrefractory-multiple-myeloma/.

[183] Dimopoulos M.A., Beksac M., Pour L., Delimpasi S., Vorobyev V., Quach H., Spicka I., Radocha J., Robak P., Kim K. (2024). Belantamab Mafodotin, Pomalidomide, and Dexamethasone in Multiple Myeloma. N. Engl. J. Med..

[184] Singh R.K., Jones R.J., Shirazi F., Qin L., Zou J., Hong S., Wang H., Lee H.C., Patel K.K., Wan J. (2024). Novel Anti-B-Cell Maturation Antigen Alpha-Amanitin Antibody-Drug Conjugate HDP-101 Shows Superior Activity to Belantamab Mafodotin and Enhanced Efficacy in Deletion 17p Myeloma Models. Research Square.

[185] Heidelberg Pharma AG (2024). A Phase 1/2a, First-in-Human Study to Evaluate the Safety, Tolerability, Pharmacokinetics, and Efficacy of HDP-101 in Patients with Plasma Cell Disorders Including Multiple Myeloma.

[186] Hangzhou Zhongmei Huadong Pharmaceutical Co., Ltd. (2026). A Phase 1 Study to Evaluate the Safety, Tolerability, Pharmacokinetics, and Efficacy of HDP-101 in Chinese Patients with Plasma Cell Disorders Including Multiple Myeloma.

[187] Heidelberg Pharma Receives Fast Track Designation from the US FDA for Its Lead ADC Candidate HDP-101 for the Treatment of Multiple Myeloma—Drugs.Com MedNews. https://www.drugs.com/clinical_trials/heidelberg-pharma-receives-fast-track-designation-us-fda-lead-adc-candidate-hdp-101-multiple-myeloma-22216.html.

[188] Kelly K.R., Ailawadhi S., Siegel D.S., Heffner L.T., Somlo G., Jagannath S., Zimmerman T.M., Munshi N.C., Madan S., Chanan-Khan A. (2021). Indatuximab Ravtansine plus Dexamethasone with Lenalidomide or Pomalidomide in Relapsed or Refractory Multiple Myeloma: A Multicentre, Phase 1/2a Study. Lancet Haematol..

[189] Jagannath S., Heffner L.T., Ailawadhi S., Munshi N.C., Zimmerman T.M., Rosenblatt J., Lonial S., Chanan-Khan A., Ruehle M., Rharbaoui F. (2019). Indatuximab Ravtansine (BT062) Monotherapy in Patients with Relapsed and/or Refractory Multiple Myeloma. Clin. Lymphoma Myeloma Leuk..

[190] Schönfeld K., Zuber C., Pinkas J., Häder T., Bernöster K., Uherek C. (2017). Indatuximab Ravtansine (BT062) Combination Treatment in Multiple Myeloma: Pre-Clinical Studies. J. Hematol. Oncol..

[191] Hamadani M., Collins G.P., Caimi P.F., Samaniego F., Spira A., Davies A., Radford J., Menne T., Karnad A., Zain J.M. (2021). Camidanlumab Tesirine in Patients with Relapsed or Refractory Lymphoma: A Phase 1, Open-Label, Multicentre, Dose-Escalation, Dose-Expansion Study. Lancet Haematol..

[192] Herrera A.F., Ansell S.M., Zinzani P.L., Radford J., Maddocks K., Pinto A., Collins G.P., Bachanova V., Bartlett N.L., Bence-Bruckler I. (2025). Camidanlumab Tesirine for Relapsed or Refractory Classic Hodgkin Lymphoma: A Phase 2 Study. Blood Adv..

[193] The ASCO Post Staff BPDCN: FDA Approves Pivekimab Sunirine-Pvzy. https://ascopost.com/news/may-2026/bpdcn-fda-approves-pivekimab-sunirine-pvzy/.

[194] Pemmaraju N., Marconi G., Montesinos P., Lane A.A., Mazzarella L., Sallman D.A., Ulrickson M.L., Schiller G.J., Erba H.P., Wang E.S. (2026). Pivekimab Sunirine in Blastic Plasmacytoid Dendritic Cell Neoplasm. J. Clin. Oncol..

[195] Daver N.G., Montesinos P., DeAngelo D.J., Wang E.S., Papadantonakis N., Todisco E., Sweet K.L., Pemmaraju N., Lane A.A., Torres-Miñana L. (2024). Pivekimab Sunirine (IMGN632), a Novel CD123-Targeting Antibody-Drug Conjugate, in Relapsed or Refractory Acute Myeloid Leukaemia: A Phase 1/2 Study. Lancet Oncol..

[196] Al Malki M.M., Minden M.D., Rich E.S., Hill J.E., Gill S.C., Fan A., Fredericks C.E., Fathi A.T., Abdul-Hay M. (2025). Safety, Tolerability, and Pharmacokinetics of ASP1235 in Relapsed or Refractory Acute Myeloid Leukemia: A Phase 1 Study. Leuk. Res..

[197] Bergamaschi C., Gaspar M., Ciucci T., Sitnikova S.I., Cayatte C., Pica M., Dovedi S.J. (2025). Innovative Strategies for T Cell Engagers for Cancer Immunotherapy. mAbs.

[198] Majzner R.G., Mackall C.L. (2019). Clinical Lessons Learned from the First Leg of the CAR T Cell Journey. Nat. Med..

[199] Bot A., Scharenberg A., Friedman K., Guey L., Hofmeister R., Andorko J.I., Klichinsky M., Neumann F., Shah J.V., Swayer A.J. (2026). In Vivo Chimeric Antigen Receptor (CAR)-T Cell Therapy. Nat. Rev. Drug Discov..

[200] FDA Label. https://nctr-crs.fda.gov/fdalabel/ui/spl-summaries/criteria/378067.

[201] Ong M.Z., Kimberly S.A., Lee W.-H., Ling M., Lee M., Tan K.-W., Foo J.-B., Yow H.-Y., Sellappans R., Hamzah S. (2024). FDA-Approved CAR T-Cell Therapy: A Decade of Progress and Challenges. Curr. Pharm. Biotechnol..

[202] Laetsch T.W., Maude S.L., Rives S., Hiramatsu H., Bittencourt H., Bader P., Baruchel A., Boyer M., De Moerloose B., Qayed M. (2023). Three-Year Update of Tisagenlecleucel in Pediatric and Young Adult Patients with Relapsed/Refractory Acute Lymphoblastic Leukemia in the ELIANA Trial. J. Clin. Oncol..

[203] Novartis Pharmaceuticals (2026). A Phase II Trial of Tisagenlecleucel in First-Line High-Risk (HR) Pediatric and Young Adult Patients with B-Cell Acute Lymphoblastic Leukemia (B-ALL) Who Are Minimal Residual Disease (MRD) Positive at the End of Consolidation (EOC) Therapy.

[204] Medical University of South Carolina (2025). A Phase 1b Dose Escalation Study of Metabolically Fit CD19 Chimeric Antigen Receptor (CAR) T Cells with CD34 Selection Markers in Adult Patients with Relapsed or Refractory CD19 B-Cell Non-Hodgkin Lymphoma and Chronic Lymphocytic Leukemia/Small Lymphocytic Lymphoma.

[205] Khalifeh M., Hopewell E., Salman H. (2025). CAR-T Cell Therapy for Treatment of Acute Myeloid Leukemia, Advances and Outcomes. Mol. Ther..

[206] Nogami A., Sasaki K. (2022). Therapeutic Advances in Immunotherapies for Hematological Malignancies. Int. J. Mol. Sci..

[207] Naik S., Velasquez M.P., Gottschalk S. (2024). Chimeric Antigen Receptor T-Cell Therapy in Childhood Acute Myeloid Leukemia: How Far Are We from a Clinical Application?. Haematologica.

[208] Yin J., Cui Q.-Y., Dai H.-P., Qu C.-J., Li Z., Kang L.-Q., Cui W., Tian X.-P., Zhu X.-M., Yu L. (2025). CD19 CAR-T in Relapsed t(8;21) AML: A Single-Center Prospective Phase II Clinical Trial. J. Hematol. Oncol..

[209] Budde L., Song J.Y., Kim Y., Blanchard S., Wagner J., Stein A.S., Weng L., Del Real M., Hernandez R., Marcucci E. (2017). Remissions of Acute Myeloid Leukemia and Blastic Plasmacytoid Dendritic Cell Neoplasm Following Treatment with CD123-Specific CAR T Cells: A First-in-Human Clinical Trial. Blood.

[210] Kite, A Gilead Company (2025). A Phase 1 Open-Label, Multicenter Study Evaluating the Safety of KITE-222, an Autologous Anti-CLL-1 CAR T-Cell Therapy, in Subjects with Relapsed/Refractory Acute Myeloid Leukemia.

[211] Vor Biopharma (2025). Phase 1/2 Study of Donor-Derived Anti-CD33 Chimeric Antigen Receptor Expressing T Cells (VCAR33) in Patients with Relapsed or Refractory Acute Myeloid Leukemia After Allogeneic Hematopoietic Cell Transplantation.

[212] Ma Y.-J., Dai H.-P., Cui Q.-Y., Cui W., Zhu W.-J., Qu C.-J., Kang L.-Q., Zhu M.-Q., Zhu X.-M., Liu D.-D. (2022). Successful Application of PD-1 Knockdown CLL-1 CAR-T Therapy in Two AML Patients with Post-Transplant Relapse and Failure of Anti-CD38 CAR-T Cell Treatment. Am. J. Cancer Res..

[213] Bmbs K.D.C. (2017). Treating Relapsed/Refractory (RR) AML with Biodegradable Anti-CD123 CAR Modified T Cells. Blood.

[214] Budde L.E., Song J., Real M.D., Kim Y., Toribio C., Wood B., Wagner J., Marcucci E., Stein A., Marcucci G. (2020). Abstract PR14: CD123CAR Displays Clinical Activity in Relapsed/Refractory (r/r) Acute Myeloid Leukemia (AML) and Blastic Plasmacytoid Dendritic Cell Neoplasm (BPDCN): Safety and Efficacy Results from a Phase 1 Study. Cancer Immunol. Res..

[215] Bhagwat A.S., Torres L., Shestova O., Shestov M., Mellors P.W., Fisher H.R., Farooki S.N., Frost B.F., Loken M.R., Gaymon A.L. (2024). Cytokine-Mediated CAR T Therapy Resistance in AML. Nat. Med..

[216] Naik S., Renee M., Talleur A.C., Epperly R., Lockey T., Bran J., Tian L., Li Y., Keerthi D., Boyd A. (2024). CD123-CAR T Cells Manufactured in the Presence of Dasatinib Induce High Grade CRS and/or IEC-HS without Improving Efficacy in Pediatric Patients with Recurrent/Refractory Leukemia. Blood.

[217] Ehninger G., Kraus S., Sala E., Metzelder S.K., Vucinic V., Fiedler W., Goebeler M.-E., Middeke J.M., von Bonin M., Kreissig C. (2022). Phase 1 Dose Escalation Study of the Rapidly Switchable Universal CAR-T Therapy Unicar-T-CD123 in Relapsed/Refractory AML. Blood.

[218] Sallman D.A., DeAngelo D.J., Pemmaraju N., Dinner S., Gill S., Olin R.L., Wang E.S., Konopleva M., Stark E., Korngold A. (2022). Ameli-01: A Phase I Trial of UCART123v1.2, an Anti-CD123 Allogeneic CAR-T Cell Product, in Adult Patients with Relapsed or Refractory (R/R) CD123+ Acute Myeloid Leukemia (AML). Blood.

[219] Zhang X., Lu W., Zhang W., He X., Zhang Y., Jin X., Zhang M., Wang Y., Guan X., Zhang R. (2025). Updated Data of CLL1 CAR-T Cell Therapy in Adult Patients with Relapsed/Refractory Acute Myeloid Leukemia. J. Hematol. Oncol..

[220] Clinical Study Protocol A Phase 1 Open-Label, Multicenter Study Evaluating the Safety of KITE-222, an Autologous Anti-CLL-1 CAR T-Cell Therapy, in Subjects with Relapsed/Refractory Acute Myeloid Leukemia. https://cdn.clinicaltrials.gov/large-docs/08/NCT04789408/Prot_000.pdf.

[221] Pei K., Xu H., Wang P., Gan W., Hu Z., Su X., Zhang H., He Y. (2023). Anti-CLL1-based CAR T-cells with 4-1-BB or CD28/CD27 Stimulatory Domains in Treating Childhood Refractory/Relapsed Acute Myeloid Leukemia. Cancer Med..

[222] Zhang H., Wang P., Li Z., He Y., Gan W., Jiang H. (2021). Anti-CLL1 Chimeric Antigen Receptor T-Cell Therapy in Children with Relapsed/Refractory Acute Myeloid Leukemia. Clin. Cancer Res..

[223] Tambaro F.P., Singh H., Jones E., Rytting M., Mahadeo K.M., Thompson P., Daver N., DiNardo C., Kadia T., Garcia-Manero G. (2021). Autologous CD33-CAR-T Cells for Treatment of Relapsed/Refractory Acute Myelogenous Leukemia. Leukemia.

[224] Mushtaq M.U., DiPersio J.F., Azzi J., Cooper B.W., Koehne G., Koura D., Maakaron J.E., Magenau J.M., McClune B., Rimando J.C. (2026). A Phase 1/2 Study of Donor-Derived Anti-CD33 CAR T-Cell Therapy (VCAR33) for Relapsed/Refractory AML after Allogeneic HCT. Blood.

[225] Clinical Trial Dual CD33-CLL1-CAR-T Cells in the treatment of Relapsed/Refractory Acute Myeloid Leukemia. https://clinicaltrials.gov/study/NCT05016063.

[226] Wang H., Feng S., Zhu Y., Zhang Y., Zhou Z., Nian Z., Lu X., Peng P., Wu S., Zhou L. (2025). The Tandem CD33-CLL1 CAR-T as an Approach to Treat Acute Myeloid Leukemia. Blood Transfus..

[227] CD33/CLL1 Compound CAR T Cells in Relapsed/Refractory AML. https://aml-hub.com/medical-information/cd33cll1-compound-car-t-cells-in-relapsedrefractory-aml.

[228] Zhujiang Hospital (2021). The Prospective, Multi-Center and Single-Arm Clinical Study of Chimeric Antigen Receptor T(CAR-T) Cells Therapy in Relapsed/Refractory Acute Myeloid Leukemia.

[229] Dreyzin A., Holtzman N.G., Bonifant C.L. (2025). CD123-targeting Immunotherapeutic Approaches in Acute Myeloid Leukaemia. Br. J. Haematol..

[230] Zhao X., Ming X., Wu J., Zhu X., Xiao Y. (2026). Next-Generation CAR-T Therapy for Acute Myeloid Leukemia: Bridging Innovation with Clinical Translation. Ann. Hematol..

[231] Lu P., Zhang X., Yang J., Li J., Qiu L., Gong M., Wang H., Chen J., Liu H., Xiong M. (2025). Nanobody-Based Naturally Selected CD7-Targeted CAR-T Therapy for Acute Myeloid Leukemia. Blood.

[232] Glisovic-Aplenc T., Diorio C., Chukinas J.A., Veliz K., Shestova O., Shen F., Nunez-Cruz S., Vincent T.L., Miao F., Milone M.C. (2023). CD38 as a Pan-Hematologic Target for Chimeric Antigen Receptor T Cells. Blood Adv..

[233] Cui Q., Liang P., Dai H., Cui W., Cai M., Ding Z., Ma Q., Yin J., Li Z., Liu S. (2022). Case Report: CD38-Directed CAR-T Cell Therapy: A Novel Immunotherapy Targeting CD38- Positive Blasts Overcomes TKI and Chemotherapy Resistance of Myeloid Chronic Myeloid Leukemia in Blastic Phase. Front. Immunol..

[234] Peschke J.C., Bergmann R., Mehnert M., Gonzalez Soto K.E., Loureiro L.R., Mitwasi N., Kegler A., Altmann H., Wobus M., Neuber C. (2024). Corrigendum to ‘FLT3-Directed UniCAR T-Cell Therapy of Acute Myeloid Leukaemia. Br. J. Haematol..

[235] Wu Z., Zhang H., Wu M., Peng G., He Y., Wan N., Zeng Y. (2021). Targeting the NKG2D/NKG2D-L Axis in Acute Myeloid Leukemia. Biomed. Pharmacother..

[236] Sallman D.A., Kerre T., Havelange V., Poiré X., Lewalle P., Wang E.S., Brayer J.B., Davila M.L., Moors I., Machiels J.-P. (2023). CYAD-01, an Autologous NKG2D-Based CAR T-Cell Therapy, in Relapsed or Refractory Acute Myeloid Leukaemia and Myelodysplastic Syndromes or Multiple Myeloma (THINK): Haematological Cohorts of the Dose Escalation Segment of a Phase 1 Trial. Lancet Haematol..

[237] Pérez-Amill L., Armand-Ugón M., Val-Casals M., Martín-Herreros B., Álamo J.R., Peña S., Frigola G., Altuna A., Santos C., Guijarro F. (2025). A Novel Chimeric Antigen Receptor T-Cell Therapy Targeting CD84 for the Treatment of Acute Myeloid and T-Cell Lymphoblastic Leukemias. Leukemia.

[238] Rab S.O., Zwamel A.H., Bishoyi A.K., Ballal S., Singh A., Devi A., Sharma G.C., Bhakuni P.N., Rizaev J. (2025). Exploring SLAMF5/CD84 in Cancer: Advancing the Frontiers of Tumor Immunology. Cell Biol. Int..

[239] First Affiliated Hospital of Zhejiang University (2025). Clinical Study on the Safety and Efficacy of Autologous CD84 Chimeric Antigen Receptor T-Cell Therapy for Adult Relapsed/Refractory Acute Myeloid Leukemia.

[240] Gao X., Liu H., Li G., Zhou Y., Dong Y., Chen X., Fan W., Li E., Wang S., Luan Q. (2026). CD96 as a Therapeutic Target for CAR T-Cell Therapy in Acute Myeloid Leukemia. Mol. Ther..

[241] Cui H., Zhao C., Li T., Wang C., Wang Y., Shan L., Feng X., Qiu X., Chen X. (2026). Identification of Siglec-15 as a Novel Target for CAR-T Cell Therapy in Acute Myeloid Leukemia. Int. Immunopharmacol..

[242] Xiao Y., Liu L., Liu S., Wang L., Tian J., Shao Z., Shi J., Cui C. (2026). Preclinical Advances and Mechanistic Insights of CAR-T Therapy for Acute Myeloid Leukemia: From Target Iteration to Microenvironment Regulation. Ann. Med..

[243] Nicod C., da Rocha M.N., Warda W., Roussel X., Haderbache R., Seffar E., Trad R., Bouquet L., Goncalves M., Bosdure L. (2023). CAR-T Cells Targeting IL-1RAP Produced in a Closed Semiautomatic System Are Ready for the First Phase I Clinical Investigation in Humans. Curr. Res. Transl. Med..

[244] Sedloev D., Chen Q., Unglaub J.M., Schanda N., Hao Y., Besiridou E., Neuber B., Schmitt A., Raffel S., Liu Y. (2024). Proteasome Inhibition Enhances the Anti-Leukemic Efficacy of Chimeric Antigen Receptor (CAR) Expressing NK Cells against Acute Myeloid Leukemia. J. Hematol. Oncol..

[245] Novoa Arias M.S. (2025). Chimeric Antigen Receptor-Engineered Natural Killer (CAR-NK) Cell Therapy in Acute Myeloid Leukemia: A Systematic Review of the Literature. Cureus.

[246] Huang R., Wang X., Yan H., Tan X., Ma Y., Wang M., Han X., Liu J., Gao L., Gao L. (2025). Safety and Efficacy of CD33-Targeted CAR-NK Cell Therapy for Relapsed/Refractory AML: Preclinical Evaluation and Phase I Trial. Exp. Hematol. Oncol..

[247] Vilcot F., Brugère C.-A., Fuentealba J., Houot R. (2026). In Vivo CAR Therapies: Turning the Patient into Their Own CAR Factory. Hemasphere.

[248] Huang W., Chen D., Chou Y.-C., Chen T.-C., Wu C.-J. (2026). Abstract 145: CD8 Nanobody-Targeted Lipid Nanoparticles Encapsulating CD13 nanoCAR Circular RNAs for in Situ CAR-T Cell Eradication of Acute Myeloid Leukemia. Cancer Res..

[249] Cancer Types—Find Your Cancer Type. https://www.cancer.org/cancer/types.html.

[250] Correnti C.E., Laszlo G.S., de van der Schueren W.J., Godwin C.D., Bandaranayake A., Busch M.A., Gudgeon C.J., Bates O.M., Olson J.M., Mehlin C. (2018). Simultaneous Multiple Interaction T-Cell Engaging (SMITE) Bispecific Antibodies Overcome Bispecific T-Cell Engager (BiTE) Resistance Via CD28 Co-Stimulation. Leukemia.

[251] Dal Bo M., Gambirasi M., Vruzhaj I., Cecchin E., Pishdadian A., Toffoli G., Safa A. (2025). Targeting Aging Hallmarks with Monoclonal Antibodies: A New Era in Cancer Immunotherapy and Geriatric Medicine. Int. J. Mol. Sci..

[252] Yang Y., Fan L., Li M., Wang Z. (2025). Immune Senescence: A Key Player in Cancer Biology. Semin. Cancer Biol..

[253] Tao Z., Chyra Z., Kotulová J., Celichowski P., Mihályová J., Charvátová S., Hájek R. (2024). Impact of T Cell Characteristics on CAR-T Cell Therapy in Hematological Malignancies. Blood Cancer J..

[254] Shiravand Y., Khodadadi F., Kashani S.M.A., Hosseini-Fard S.R., Hosseini S., Sadeghirad H., Ladwa R., O’Byrne K., Kulasinghe A. (2022). Immune Checkpoint Inhibitors in Cancer Therapy. Curr. Oncol..

[255] Lau H.-C., Kwong Y.-L. (2026). Immune Checkpoint Blockade in Hematological Malignancies: Current Status and Future Directions. Cancers.

