# Supplementary material for: Advances in Oncohematology Immunotherapy: Monoclonal Antibodies and CAR T-Cell Therapies
Source: Cells. 2026 Aug 10;15(16):1439. doi: 10.3390/cells15161439 (PMC13510469; doi:10.3390/cells15161439)
Supplement: Supplementary file 1 [file cells-15-01439-s001.zip › cells-4404920-supplementary.pdf]

Table S1: Table summarizes the targets investigated in research on CAR-T cell targeting AML.

Clinical trial	Clinical trial identifiant	Phase	Indication	Study Start	Study Completion	Drug
NCT04789408	Study of KITE-222 in Participants With Relapsed/Refractory Acute Myeloid Leukemia	1	Relapsed/refractory acute myeloid leukemia	19/07/2021	18/05/2024	KITE-222, an autologous anti-CLL-1 CAR T-cell therapy
NCT04884984	Anti-CLL1 CAR T-cell Therapy in CLL1 Positive Relapsed/ Refractory Acute Myeloid Leukemia (AML)	1/2	CLL1-positive relapsed/refractory acute myeloid leukemia	07/01/2017	31/12/2024	Anti-CLL1 CAR-engineered T-cells
NCT06128044	CRISPR-Edited Allogeneic Anti-CLL-1 CAR-T Cell Therapy in Patients With Relapsed/ Refractory Acute Myeloid Leukemia (AMpLify)	1	Adult with relapsed/ refractory acute myeloid leukemia	08/02/2024	30/05/2025	CB-012, an allogeneic CAR T-cell therapy that targets C-type lectin-like molecule-1 (CLL-1)
NCT05023707	Anti-FLT3 CAR T-cell Therapy in FLT3 Positive Relapsed/ Refractory Acute Myeloid Leukemia	1/2	FLT3-positive relapsed/refractory acute myeloid leukemia	31/12/2021	30/06/2025	Anti-FLT3 CAR-engineered T-cells

NCT05445011	Anti-FLT3 CAR-T Cell (TAA05 Cell Injection) in the Treatment of Relapsed / Refractory Acute Myeloid Leukemia	1	Relapsed/refractory acute myeloid leukemia	14/06/2022	14/06/2027	Anti-FLT3 CAR T-cells
NCT06786533	Clinical Study of Anti-FLT3 CAR-T Cells for the Treatment of Relapsed/Refractory AML	1	Relapsed/Refractory acute myeloid leukemia	23/01/2025	01/01/2027	Anti-FLT3 CAR T-cells
NCT06760260	Evaluation of the Safety and Efficacy of Human CI-135 (FLT3) Targeted CAR-T Cells Injection for Subjects with Relapsed/Refractory Acute Myeloid Leukemia	1	Relapsed/refractory acute myeloid leukemia	26/01/2022	32/12/2026	Anti-FLT3 CAR T-cells
NCT07144020	Donor Derived CD117 CAR-T Cells in the Treatment of R/R Acute Myeloid Leukemia	Early phase 1	Relapsed/refractory acute myeloid leukemia	05/09/2025	05/09/2028	Anti-CD117 CAR T-cells
NCT04014881	Safety and Efficacy of Anti-CD123 CAR-T Therapy in Patients With Refractory/Relapsed	1	Relapsed/refractory CD123+ acute myeloid leukemia	06/07/2019	01/07/2022	Anti-CD123 CAR T-cells

	CD123+ Acute Myeloid Leukemia					
NCT03631576	CD123/CLL1 CAR-T Cells for R/R AML (STPHI_0001)	2/3	Relapsed/refractory acute myeloid leukemia	18/08/2018	10/08/2021	Anti-CD123/CLL1 CAR T-cells
NCT04010877	Multiple CAR-T Cell Therapy Targeting AML	1/2	Relapsed/refractory acute myeloid leukemia	01/08/2019	31/12/2023	CLL-1, CD33 and/or CD123-specific CAR gene-engineered T-cells
NCT04265963	CD123-Targeted CAR-T Cell Therapy for Relapsed/Refractory Acute Myeloid Leukemia	1/2	Relapsed/refractory acute myeloid leukemia	01/09/2019	01/07/2024	Anti-CD123 CAR T-cells
NCT07201727	CD123+CLL-1 CAR-T Sequential Infusion With CD7 CAR-T and Bridging to Allo-HSCT for Relapsed/Refractory Acute Myeloid Leukemia	Early phase 1	Relapsed/refractory acute myeloid leukemia	30/05/2025	01/07/2028	CD123+CLL-1 dual-target CAR T-cells<br>Anti-CD7 CAR T-cells
NCT05995041	Universal CAR-T Cells Targeting AML	1	Relapsed/refractory acute myeloid leukemia	31/10/2023	31/12/2026	Universal CAR T-cell products targeting CLL-1, CD33, CD38 and CD123

NCT03125577	Combination CAR-T Cell Therapy Targeting Hematological Malignancies	1/2	B cell malignancies	01/08/2025	31/12/2029	4SCAR19 and 4SCAR22<br>4SCAR19 and 4SCAR38<br>4SCAR19 and 4SCAR20<br>4SCAR19 and 4SCAR123<br>4SCAR19 and 4SCAR70<br>4SCAR19 and 4SCAR30
NCT03222674	Multi-CAR T Cell Therapy for Acute Myeloid Leukemia	1/2	Acute myeloid leukemia	15/07/2017	31/12/2020	Muc1/CLL1/CD33/CD38/CD56/CD123-specific gene-engineered T-cells
NCT04599543	IL3 CAR-T Cell Therapy for Patients With CD123 Positive Relapsed and/or Refractory Acute Myeloid Leukemia	Early Phase 1	Relapsed/refractory acute myeloid leukemia	15/11/2020	15/11/2026	Anti-IL3 CAR T-cell therapy
NCT06125652	Administration of Anti Tim-3/CD123 CAR-T Cell Therapy in Relapsed and Refractory Acute Myeloid Leukemia (rr/ AML)	1/2	Relapsed/refractory acute myeloid leukemia	04/11/2023	01/01/2027	Anti-Tim3/CD123 CAR T-cells
NCT04272125	Safety and Efficacy of CD123-Targeted CAR-T Therapy for Relapsed/ Refractory Acute Myeloid Leukemia	1/2	Relapsed/refractory acute myeloid leukemia	01/12/2019	01/07/2024	CD123-targeted CAR T-cells

NCT06765876	CART123 T Cells in Relapsed or Refractory CD123+ Hematologic Malignancies: a Dose Escalation Phase I Trial (UHKT-CAR123-01)	Early phase 1	Relapsed/refractory CD123+ hematologic malignancies	23/10/2024	31/12/2028	CART123, anti-CD123 CAR-modified autologous T-cells
NCT05949125	Phase 1 Study of Allo-RevCAR01-T-CD123 in Patients With Selected CD123 Positive Hematologic Malignancies	1	Selected hematologic malignancies positive for CD123	03/01/2024	01/01/2028	Allo-RevCAR01-T-CD123, consisting of genetically modified T-cells carrying reverse CARs (Allo RevCAR01 T) in combination with CD123 target module (R-TM123)
NCT04230265	Phase 1 Study of UniCAR02-T-CD123 in Patients With Selected CD123 Positive Hematologic Malignancies	1	Hematologic and lymphatic malignancies positive for CD123 marker	28/01/2020	01/09/2025	Genetically modified T-cells carrying universal CARs (UniCAR02-T) in combination with CD123 target module (TM123)
NCT03190278	Study Evaluating Safety and Efficacy of UCART123v1.2 in Patients With Relapsed/ Refractory Acute Myeloid Leukemia (AMELI-01)	1	Relapsed/refractory acute myeloid leukemia	19/06/2017	01/12/2025	UCART123v1.2, universal CAR T-cells targeting CD123

NCT03203369	Study to Evaluate the Safety and Clinical Activity of UCART123 in Patients With BPDCN (ABC123)	1	Relapsed/refractory blastic plasmacytoid dendritic cell neoplasm (BPDCN)	28/06/2017	27/06/2019	UCART123, universal CAR T-cells targeting CD123
NCT02159495	Genetically Modified T-cell Immunotherapy in Treating Patients With Relapsed/Refractory Acute Myeloid Leukemia and Persistent/Recurrent Blastic Plasmacytoid Dendritic Cell Neoplasm	1	CD123+ relapsed/refractory acute myeloid leukemia	2015-12-15	06/08/2026	CD123+ CAR T-cells
NCT02958397	A Clinical Research of CD33-Targeted CAR-T in Myeloid Malignancies	1/2	Myeloid malignancies	01/10/2016	01/10/2020	CD33-targeted CAR T-cells
NCT05445765	Anti-CD33 CAR-T Cells for the Treatment of Relapsed/Refractory CD33+ Acute Myeloid Leukemia	1	Relapsed/refractory, high risk hematologic malignancies	01/07/2022	30/06/2024	Anti-CD33 CAR T-cells
NCT06762132	A Clinical Study to Explore the Safety and Efficacy of CD33 CAR-T Cell in Relapsed/	Early phase 1	Relapsed/refractory acute myeloid leukemia	10/01/2025	30/10/2027	Anti-CD33 CAR T-Cell

	Refractory Acute Myeloid Leukemia					
NCT05473221	Evaluate the Safety and Efficacy of CD33 CAR-T in Patients With R/R AML	1	Refractory/relapsed acute myeloid leukemia	02/08/2022	02/08/2025	Anti-CD33 CAR T-cells
NCT05467254	Evaluate the Safety and Efficacy of CLL1+CD33 CAR-T in Patients With R/R AML	1	Refractory/relapsed acute myeloid leukemia	02/08/2022	02/08/2025	Anti-CLL1/CD33 CAR T-cells
NCT06420063	Sequential CAR-T Cells Targeting CD33/CD123 in Patients With Acute Myelocytic Leukemia AML (BAH244)	1/2	Refractory/relapsed acute myeloid leukemia	10/07/2024	28/12/2026	Anti-CD123/CD33 CAR T-cells
NCT06326021	Optimised CD33 (FL-33) CAR T Therapy for Refractory/Relapsed Acute Myeloid Leukaemia	1	Refractory/relapsed acute myeloid leukemia	02/04/2024	30/12/2026	Autologous anti-CD33 CAR T-cells
NCT05016063	Dual CD33-CLL1-CAR-T Cells in the Treatment of Relapsed/Refractory Acute Myeloid Leukemia	Early phase 1	Relapsed/refractory acute myeloid leukemia	01/09/2021	01/09/2023	Anti-CD33/CLL1 CAR T-cells

NCT05984199	Donor-Derived Anti-CD33 CAR T Cell Therapy (VCAR33) in Patients With Relapsed or Refractory AML After Allogeneic Hematopoietic Cell Transplant	1/2	Relapsed/refractory acute myeloid leukemia after allogeneic hematopoietic cell transplant	11/12/2023	28/05/2025	VCAR33, lentiviral-transduced CD33-directed CAR T-cells
NCT04835519	Phase I/II Study of Enhanced CD33 CAR T Cells in Subjects With Relapsed or Refractory Acute Myeloid Leukemia	1/2	Relapsed/refractory acute myeloid leukemia	08/04/2021	19/05/2023	Anti-CD33 CAR T-cells
NCT03126864	Study of Adoptive Cellular Therapy Using Autologous T Cells Transduced With Lentivirus to Express a CD33 Specific Chimeric Antigen Receptor in Patients With Relapsed or Refractory CD33-Positive Acute Myeloid Leukemia	1	Relapsed/refractory CD33+ acute myeloid leukemia	04/08/2017	10/10/2019	Anti-CD33 CAR T-cells

NCT05105152	PLAT-08: A Study Of SC-DARIC33 CAR T Cells In Pediatric And Young Adults With Relapsed Or Refractory CD33+ AML	1	Children and young adults with relapsed/refractory CD33+ acute myeloid leukemia	29/11/2021	31/01/2041	SC-DARIC33
NCT05672147	CD33-CAR T Cell Therapy for the Treatment of Recurrent or Refractory Acute Myeloid Leukemia	1	Recurrent/refractory acute myeloid leukemia	07/12/2023	03/09/2028	Anti-CD33 CAR T-cells
NCT03971799	Study of Anti-CD33 Chimeric Antigen Receptor-Expressing T Cells (CD33CART) in Children and Young Adults With Relapsed/ Refractory Acute Myeloid Leukemia	1/2	Children and young adults with relapsed/refractory acute myeloid leukemia	08/01/2020	01/12/2039	Anti-CD33 CAR T-cells (autologous vs allogeic)
NCT05239689	Clinical Study of CD38 CAR-T Cells in the Treatment of Hematological Malignancies	Early phase 1	CD38+ relapsed/ refractory acute myeloid leukemia	28/02/2022	05/12/2024	Anti-CD38 CAR T-cells
NCT06880354	A Clinical Study to Evaluate the Safety and Efficacy of CLL1 and CD38 Dual-Target	1	Relapsed/refractory acute myeloid leukemia	22/05/2025	31/03/2028	CLL1 and CD38 Dual-Target CAR T-cell: lentiviral gene-modified autologous CAR T-cell product that targets both CLL1 and CD38

	CAR-T Cell Injection in the Treatment of Relapsed or Refractory Acute Myeloid Leukemia					
NCT04351022	CD38-targeted Chimeric Antigen Receptor T Cell (CART) in Relapesd or Refractory Acute Myeloid Leukemia	1/2	CD38+ relapsed/refractory acute myeloid leukemia	01/07/2017	31/12/2023	CD38-Targeted CAR T-cells
NCT06786299	Clinical Study on the Safety and Efficacy of Autologous CD84 Chimeric Antigen Receptor T-Cell Therapy for Adult Relapsed/Refractory Acute Myeloid Leukemia.	Early Phase 1	Relapsed/refractory acute myeloid leukemia	2025-02-10	2026-12-31	Anti-CD84 CAR T-Cell therapy
NCT04599556	Clinical Trial for the Safety and Efficacy of Anti-CD7 CAR-T Cell Therapy for Patients With Relapsed or Refractory CD7 Positive Hematological Malignancy	Phase 1	Relapsed/Refractory CD7+ T-ALL/LBL, T-NHL and AML	01/04/2021	30/12/2025	Anti-CD7 CAR T-cells

NCT07153068	SupCD7 CART for Relapsed or Refractory CD7 Positive Hematologic Malignancies	1/2	Relapsed/refractory acute myeloid leukemia, relapsed/refractory T lymphoblastic leukemia/lymphoma (T-ALL/LBL)	28/06/2025	28/06/2030	supCD7 CAR T-cells
NCT07109518	uCD7 CART for Relapsed or Refractory CD7 Positive Hematologic Malignancies	01-janv	Relapsed/refractory acute myeloid leukemia, relapsed/refractory T lymphoblastic leukemia/lymphoma (T-ALL/LBL)	28/06/2025	28/06/2030	Universal anti-CD7 CAR T-cells
NCT07120607	Allogeneic CD7 CAR γδ T Cells Therapy Recurrent/Refractory Leukemia	1	Refractory/relapsed CD7+ hematological malignancies (T-ALL/LBL, AML)	18/08/2025	31/12/2027	Anti-CD7 CAR-γδ T-cell
NCT05454241	CD7 CAR-T for Patients With r/r CD7+ Hematologic Malignancies	2	Refractory/relapsed CD7+ hematological malignancies (T-ALL/LBL, AML)	07/09/2022	31/08/2024	Universal anti-CD7 CAR T-cells
NCT05907603	Research Development（RD）13-02 Chimeric Antigen Receptor(CAR) -T Cell Injection for Patients With r/r Cluster Of	Early phase 1	CD7+ relapsed/refractory T-ALL/LBL/AML	08/03/2023	31/08/2024	Anti-CD7 CAR T-cells

	Differentiation 7（CD7）+ T-Acute Lymphoblastic Leukemia(ALL)/T-Lymphoblastic Lymphoma(LBL) / Acute Myelogenous Leukemia(AML)					
NCT04033302	Multi-CAR T Cell Therapy Targeting CD7-positive Malignancies	1/2	CD7-positive malignancies (T-ALL, TCL, AML, NK lymphoma)	01/09/2019	31/12/2023	Anti-CD7 CAR T-cells
NCT05377827	Dose-Escalation and Dose-Expansion Study to Evaluate the Safety and Tolerability of Anti-CD7 Allogeneic CAR T-Cells (WU-CART-007) in Patients With CD7+ Hematologic Malignancies	1	CD7+ relapsed/refractory hematologic malignancies	10/10/2023	30/04/2026	WU-CART-007, a CD7-directed, genetically modified, allogeneic, fratricide-resistant CAR
NCT05995028	Universal 4SCAR7U Targeting CD7-positive Malignancies	1	CD7-positive hematological malignancies	31/10/2023	31/12/2026	Universal CD7-specific CAR gene-engineered T cells

NCT04762485	Humanized CD7 CAR T-cell Therapy for r/r CD7+ Acute Leukemia	1/2	CD7+ relapsed/refractory acute leukemia	01/06/2021	28/02/2024	Anti-CD7 CAR T-cells
NCT04938115	CD7 CAR-T cell	NA	Relapsed/refractory acute myeloid leukemia	10/05/2021	30/10/2023	Anti-CD7 CAR T-cells
NCT05445011	Anti-FLT3 CAR-T Cell (TAA05 Cell Injection) in the Treatment of Relapsed / Refractory Acute Myeloid Leukemia	1	Relapsed/refractory acute myeloid leukemia	14/06/2022	14/06/2027	Anti-FLT3 CAR-T Cell (TAA05 cell injection)
NCT06760260	Evaluation of the Safety and Efficacy of Human CI-135 (FLT3) Targeted CAR-T Cells Injection for Subjects with Relapsed/ Refractory Acute Myeloid Leukemia	Early phase 1	Relapsed/refractory Acute Myeloid Leukemia	26/01/2022	31/12/2026	Anti human CI-135 (FLT3) CAR-T Injection
NCT03896854	CART-19 T Cell in CD19 Positive Relapsed or Refractory Acute Myeloid Leukemia (AML)	1/2	CD19+ relapsed/refractory acute myeloid leukemia	18/10/2017	31/12/2025	Anti-CD19 CAR T-cells

NCT06649227	Study Investigating the Safety of CD19 CAR-T Cells in Relapsed/ Refractory AML Expressing CD19 (CARLA-M19)	1	Relapsed/Refractory acute myeloid leukemia expressing CD19	10/07/2025	10/07/2030	Anti-CD19 CAR T-cells
NCT04257175	CAR-T CD19 for Acute Myelogenous Leukemia With t 8:21 and CD19 Expression	2/3	Recurrent or irresponsible to previous treatment acute myeloid leukemia	12/02/2020	01/12/2024	Anti-CD19 CAR T-cells
NCT05488132	Administration of Anti-siglec-6 CAR-T Cell Therapy in Relapsed and Refractory Acute Myeloid Leukemia (rr/ AML)	1/2	Relapsed/refractory acute myeloid leukemia	01/04/2022	31/03/2025	Anti-Siglec-6 CAR T-cells
NCT04803929	Clinical Study of Anti-ILT3 CAR-T Therapy for R/R AML(M4/M5)	Early phase 1	Relapsed/refractory acute myeloid leukemia (M4/M5)	03/03/2021	01/03/2026	Autologous T Cells Modified With ILT3 CAR
NCT06326463	CAR T-cell Therapy Directed to CD70 for Pediatric Patients With Hematological Malignancies	1	Recurrent/refractory CD70+ hematological malignancies after lymphodepleting chemotherapy (AML, MDS, B-	16/10/2024	01/07/2031	Anti-CD70 CAR T-cells

			AAL, T-ALL, lymphoma)			
NCT06345027	Chimeric Antigen Receptor Treatment Targeting CD70 (SEVENTY) (CASEY)	1	Recurrent/refractory CD70+ hematological malignancies after lymphodepleting chemotherapy (AML, B-AAL, T-ALL)	01/02/2026	01/04/2042	Anti-CD70 CAR T-cells
NCT06492304	A Safety and Efficacy Study Evaluating CTX131 in Adult Subjects With Relapsed/ Refractory Hematologic Malignancies	1/2	Relapsed/refractory hematologic malignancies	13/08/2024	01/11/2030	CTX131, a CD70-directed CAR T-cell therapy
NCT07370064	Clinical Study of Anti-CLL1-CD33-NKG2D Bicephali CAR-T for Relapsed/ Refractory Acute Myeloid Leukemia (AML CAR-T)	1/2	Relapsed/refractory acute myeloid leukemia			Anti-CLL1-CD33-NKG2D bispecific CAR T-cells
NCT04658004	NKG2D CAR-T Cell Therapy for Patients With Relapsed and/or	Early phase 1	Relapsed/refractory acute myeloid leukemia	15/01/2021	15/01/2027	Anti-NKG2D CAR T-cell therapy

	Refractory Acute Myeloid Leukemia
